# Worse Breast Cancer Prognosis of *BRCA1/BRCA2* Mutation Carriers: What's the Evidence? A Systematic Review with Meta-Analysis

**DOI:** 10.1371/journal.pone.0120189

**Published:** 2015-03-27

**Authors:** Alexandra J. van den Broek, Marjanka K. Schmidt, Laura J. van ‘t Veer, Rob A. E. M. Tollenaar, Flora E. van Leeuwen

**Affiliations:** 1 Division of Psychosocial Research and Epidemiology, Netherlands Cancer Institute, Amsterdam, Netherlands; 2 Division of Molecular Pathology, Netherlands Cancer Institute, Amsterdam, Netherlands; 3 Department of Surgery, Leiden University Medical Centre, Leiden, Netherlands; The Adamovic Cancer Research foundation; Adamovic Research AB, SWEDEN

## Abstract

**Objective:**

Conflicting conclusions have been published regarding breast cancer survival of *BRCA1/2* mutation carriers. Here we provide an evidence-based systematic literature review.

**Methods:**

Eligible publications were observational studies assessing the survival of breast cancer patients carrying a *BRCA1/2* mutation compared to non-carriers or the general breast cancer population. We performed meta-analyses and best-evidence syntheses for survival outcomes taking into account study quality assessed by selection bias, misclassification bias and confounding.

**Results:**

Sixty-six relevant studies were identified. Moderate evidence for a worse unadjusted recurrence-free survival for *BRCA1* mutation carriers was found. For *BRCA1* and *BRCA2* there was a tendency towards a worse breast cancer-specific and overall survival, however, results were heterogeneous and the evidence was judged to be indecisive. Surprisingly, only 8 studies considered adjuvant treatment as a confounder or effect modifier while only two studies took prophylactic surgery into account. Adjustment for tumour characteristics tended to shift the observed risk estimates towards a relatively more favourable survival.

**Conclusions:**

In contrast to currently held beliefs of some oncologists, current evidence does not support worse breast cancer survival of *BRCA1/2* mutation carriers in the adjuvant setting; differences if any are likely to be small. More well-designed studies are awaited.

## Introduction

*BRCA1/2*-associated breast cancers account for about 25–30% of familial breast cancers, and for about 3% of all breast cancers [1]. *BRCA1*-associated breast cancers differ from tumours not associated with *BRCA* mutations with respect to pathological features, e.g. they are more often estrogen receptor negative and high grade and have a higher frequency of somatic abnormalities in prognostically important genes such as *P53* [2,3]. The biological background of *BRCA1/2* [4] and different pathological aspects of *BRCA1*-associated tumours support the hypothesis that patients carrying a *BRCA1* and/or *BRCA2* mutation might have a worse breast cancer prognosis compared to non-carriers.

An impressive number of studies have already been conducted to address the association between *BRCA1* and/or *BRCA2* mutation carriership and breast cancer survival (Table 1 and Table 2). Study results were inconsistent, possibly due to differences in study design, study size, study populations and methodological rigor. Yet, accurate estimation of the effect of carriership, independent of tumour characteristics, on breast cancer survival is needed to optimize treatment choices and surveillance policies for *BRCA* mutation carriers with breast cancer.

**Table 1:** Characteristics and quality scores of studies included in the review (N = 66).

Author + year	Country	Study type	Types of patients included			‘Non-carrier group’ tested	Carriers/ ‘non-carriers’ matched?	Factors matched			Diagnose years of breast cancer (incl period)	N of carriers		N of ‘non-carriers’	Quality score			Selection + Misclass bias		All biases together	
			Carriers		‘Non-carrier group’			age	Year	Stage/grade		B1	B2		Selection bias	Misclass bias	Confoun-ding	Total Score	% max	Total Score	% max
Ansquer, 1998	France	Unselected cohort	age diagnosis < 36			Yes	NA				1/1990–12/1995	15	NA	108	151.5	84	120	235.5	*59*	355.5	*59*
Arun, 2011	United States	CGC based with int. ref.	From CGC, all received NST			Yes	NA				Not reported	57	23	237	108	84	46	192	*48*	238	*40*
Bayraktar, 2011	United States	CGC based with int. ref.	From CGC, with triple negative tumours			Yes	NA				1997–2010	94	20	101	108	84	200	192	*48*	392	*65*
Bonadona, 2007	France	Unselected cohort	age diagnosis < 46			Yes	NA				1/1995–1/1998	15	6	211	151.5	84	176	235.5	*59*	411.5	*69*
Brekelmans, 2007	The Netherlands	CGC based with ext. ref.	BRCA+, from CGC		FH-	No	Partly	x	x		> 1//1/1980	170	90	759	87	47	176	134	*34*	310	*52*
Brekelmans, 2007	The Netherlands	CGC based with int. ref.	From CGC			Yes	NA				> 1/1/1980	170	90	238	108	47	176	155	*39*	331	*55*
Budroni, 2009	Italy	Unselected cohort	-			Yes	NA				1997–2002	4	44	468	151.5	63	74	214.5	*54*	288.5	*48*
Carroll, 2011	Ireland	CGC based with ext. ref.	BRCA+, from CGC		FH-	No	Yes	x		x	1997–2007	16	20	108	64.5	26	59	90.5	*23*	149.5	*25*
Chappuis, 2000	Canada	Unselected cohort	A. Jewish			Yes	NA				1/1986–11/1995	24^b^	8^b^	170	300	100	176	400	*100*	576	*96*
Chappuis, 2005	Canada	Unselected cohort	A. Jewish			Yes	NA				1/1/1980–1/11/1995	30^b^	NA	248	300	100	176	400	*100*	576	*96*
Chiappetta, 2010	Italy	CGC based with int. ref.	From CGC			Yes	NA				1990–2002	31	23	62	108	63	23	171	*43*	194	*32*
Cortesi, 2010	Italy	CGC based with ext. ref.	BRCA+, from CGC		FH-	No	No				1988–2006	80	NA	4912	87	47	176	134	*34*	310	*52*
Cortesi, 2010	Italy	Unselected cohort	FH+			Yes	NA				1988–2006	80	NA	931	151.5	63	176	214.5	*54*	390.5	*65*
Cortesi, 2010	Italy	CGC based with ext. ref.	BRCA+, from CGC		From cancer registry	No	Yes	x		x	1988–2006	80	NA	320	64.5	47	176	111.5	*28*	287.5	*48*
Eccles, 2001	United Kingdom	CGC based with ext. ref.	BRCA+, from CGC		FH-	No	No				Unclear	75	NA	162	64.5	21	115	85.5	*21*	200.5	*33*
Eccles, 2001	United Kingdom	CGC based with int. ref.	from CGC			No	NA				Unclear	75	NA	67	108	21	115	129	*32*	244	*41*
Eerola, 2001	Finland	CGC based with ext. ref.	BRCA+, from CGC		From cancer registry	No	No				1953–1995	32	43	59517	87	26	59	113	*28*	172	*29*
Eerola, 2001	Finland	Unselected cohort	FH+			Yes	NA				1953–1995	32	43	284	151.5	63	59	214.5	*54*	273.5	*46*
Einbeigi, 2001	Sweden	CGC based with ext. ref.	BRCA+, from CGC		From cancer registry	No	Yes	x	x		Not reported	30	NA	120	85.5	26	0	111.5	*28*	111.5	*19*
Ellberg, 2010	Sweden	Unselected cohort	Only the CGC eligible Pts (4%) were tested			Partly	NA				Not reported	9	5	1663	235.5	63	59	298.5	*75*	357.5	*60*
El-Tamer, 2004	United States	Unselected cohort	A. jewish, age diagnosis < 65			Yes	NA				1/1989–1/1999	30^b^	21^b^	436	216	100	115	316	*79*	431	*72*
Foulkes, 1997	Canada	Unselected cohort	A. jewish, age diagnosis < 65			Yes	NA				1/1990–11/1995	12^b^	NA	100	151.5	100	120	251.5	*63*	371.5	*62*
Gaffney, 1998	United States	CGC based with ext. ref.	BRCA+, FH+		From cancer registry	No	No				1957–1994	30	20	18278	256.5	68	59	324.5	*81*	383.5	*64*
Gaffney, 1998	United States	CGC based with ext. ref.	BRCA+, FH+		From cancer registry	No	Yes	x	x	x	1957–1994	30	20	8409	256.5	68	23	324.5	*81*	347.5	*58*
Goffin, 2003	Canada	Unselected cohort	A. Jewish < 65			Yes	NA				01/1980–11/1995	30^b^	NA	248	216	100	176	316	*79*	492	*82*
Goffin, 2003	Canada	Unselected cohort	A. Jewish < 65			Yes	NA				1980–1995	28^b^	8^b^	215	300	100	120	400	*100*	520	*87*
Gonzalez-Angulo, 2011	United States	Unselected cohort	With triple negative tumours			Yes	NA				1997–2006	12	3	62	216	63	176	279	*70*	455	*76*
Goode, 2002	United Kingdom	Unselected cohort	From cancer registry			Partly (56%)	NA				> 01–1991	10	19	2400	235.5	79	120	314.5	*79*	434.5	*72*
Goodwin, 2012	United States + Canada	CGC based with int. ref.	Mostly from CGC			Partly	NA				1991–1998	94	72	1550	214.5	68	176	282.5	*71*	458.5	*76*
Hagen, 2009	Norway	CGC based with ext. ref.	BRCA+, from CGC		From cancer registry	No	Yes	x	x	x	1980–2001	167	NA	304	108	26	59	134	*34*	193	*32*
Hamann, 2000	Germany	CGC based with ext. ref.	BRCA+, from CGC		FH+ (BRCA- families)	Yes	No				1961–1994	36	NA	49	150	84	120	234	*59*	354	*59*
Heikkinen, 2009	Finland	CGC based with ext. ref.	BRCA+, from CGC		Partly FH+	Partly (25%)	No				1997–2004	67	68	2135	64·5	68	120	132·5	*33*	252·5	*42*
Huzarski, 2013	Poland	Unselected cohort	age diagnosis < 50, stage I-III			Yes	NA				1996–2006	233^b^	NA	3112	235.5	176	314.5	314.5	*79*	490.5	*82*
Jóhannsson, 1998	Sweden	CGC based with ext. ref.	BRCA+, from CGC		From cancer registry	No	No				1958–1995	40	NA	28281	151.5	26	46	177.5	*44*	223.5	*37*
Jóhannsson, 1998	Sweden	CGC based with ext. ref.	BRCA+, from CGC		From cancer registry	No	Yes	x		x	1958–1995	40	NA	112	172.5	26	176	198.5	*50*	374.5	*62*
Kirova, 2010	France	CGC based with ext. ref.	BRCA+, from CGC		FH-	No	Yes	x	x		1981–2000	19	8	54	87	47	46	134	*34*	180	*30*
Kirova, 2010	France	CGC based with int. ref.	From CGC			Yes	NA				1981–2000	19	8	107	108	63	120	171	*43*	291	*49*
Kirova, 2010	France	CGC based with ext. ref.	BRCA+, from CGC		FH-	No	Partly	x	x		1981–2000	19	8	271	87	47	120	134	*34*	254	*42*
Lee, 1999	United States	Unselected cohort	affected relatives of B+/B- (kin-cohort-analyse)			Yes	NA				Not reported	35	23	979	151.5	63	46	214.5	*54*	260.5	*43*
Lee, 2011	United States	CGC based with int. ref.	From CGC, with triple negative tumours			Yes	NA				1/1/1996–31/12/2004	46	NA	71	43.5	63	176	106.5	*27*	282.5	*47*
Loman, 2000	Sweden	CGC based with ext. ref.	BRCA+, from CGC		From cancer registry	No	Yes	x	x		1995–1999	NA	54	214	85.5	47	82	132.5	*33*	214.5	*36*
Moller, 2002	Northern europe	CGC based with int. ref.	From CGC, with N0 tumours			Yes	NA				Not reported	24	NA	108	172.5	84	84	256.5	*64*	340.5	*57*
Moller, 2007	Norway + UK	CGC based with int. ref.	From CGC (20% DCIS tumours)			Yes	NA				< 12/2005	71	22	282	214.5	63	120	277.5	*69*	397.5	*66*
Musolino, 2007	Italy	CGC based with int. ref.	age diagnoses < 40, from CGC			Yes	NA				6/1999–12/2005	10	5	41	108	37	139	145	*36*	284	*47*
Musolino, 2007	Italy	CGC based with ext. ref.	BRCA+, age diagnosis <40, from CGC		age diagnosis >45 and FH-	No	No				6/1999–12/2005	10	5	28	87	21	139	108	*27*	247	*41*
Nisman, 2010	Israel	CGC based with int. ref.	A. Jewish, from CGC			Yes	NA				5/2004–12/20078	7^b^	9^b^	66	108	63	120	171	*43*	291	*49*
Pierce, 2000	USA, Canada	CGC based with ext. ref.	BRCA+, from CGC		FH-	No	Yes	x	x		3/1980–12/1997	54	17	213	85.5	47	176	132.5	*33*	308.5	*51*
Pierce, 2006	USA + Israel	CGC based with ext. ref.	BRCA+, from CGC		FH-	No	Yes	x	x		by 04/2001	123	37	445	108	47	176	155	*39*	331	*55*
Plakhins, 2011	Latvia	CGC based with int. ref.	from CGC (selection irrespective of FH)			Yes	Yes	x	x	x	Not reported	93^b^	NA	103	108	63	144	171	*43*	315	*53*
Plakhins, 2013	Latvia	CGC based with ext. ref. ^a^	BRCA+, from CGC	BRCA-, from CGC		Yes	Yes			x	2002–2008	71b	NA	93	172.5	84	97	256.5	*64*	353.5	*59*
Rennert, 2007	Israel	Unselected cohort	A. Jewish			Yes	NA				1/1987–1/1989	76^b^	52^b^	1189	300	100	138	400	*100*	538	*90*
Rijnsburger, 2010	The Netherlands	CGC based with int. ref.	from CGC (part of a screening study)			Yes	NA				11/1999–3/2006	42		34	256.5	84	46	340.5	*85*	386.5	*64*
Robson, 1998	United States	Unselected cohort	A. Jewish, age diagnosis < 42			Yes	NA				1/1992–12/1995	28^b^		58	151.5	84	200	235.5	*59*	435.5	*73*
Robson, 1999	United States	Unselected cohort	A. Jewish			Yes	NA				1/1980–12/1990	21^b^	7^b^	277	216	100	138	316	*79*	454	*76*
Robson, 2004	United States	Unselected cohort	A. jewish			Yes	NA				1/1980–11/1995	43^b^	14^b^	440	216	100	176	316	*79*	492	*82*
Seynaeve, 2004	The Netherlands	CGC based with ext. ref.	BRCA+, from CGC		FH-	No	Partly	x	x		1980–1995	21	2	174	108	68	176	176	*44*	352	*59*
Soumittra, 2009	India	CGC based with int. ref.	From CGC			Yes	NA				Not reported	12		48	108	37	23	145	*36*	168	*28*
Stoppa-Lyonnet, 2000	France	CGC based with int. ref.	From CGC			Yes	NA				1/1991–7/1998	19	NA	91	108	84	120	192	*48*	312	*52*
Tryggvadottir, 2013	Iceland	Unselected cohort	-			Yes	NA				1955–2004	NA	215^b^	2752	235.5	100	120	335.5	*84*	455.5	*76*
Verhoog, 1998	The Netherlands	CGC based with ext. ref.	BRCA+, from CGC		FH-	No	Yes	x	x		1969–1995	49	NA	120	87	47	120	134	*34*	254	*42*
Verhoog, 1999	The Netherlands	CGC based with ext. ref.	BRCA+, from CGC		From cancer registry	No	Yes	x	x		1960–1996	NA	28	112	87	68	120	155	*39*	275	*46*
Veronesi, 2005	Italy	CGC based with int. ref.	From CGC			Yes	NA				>1997	9	30	86	108	37	61	145	*36*	206	*34*
Vinodkumar, 2007	India	Unselected cohort	FH+			Yes	NA				Not reported	11	NA	18	151.5	42	59	193.5	*48*	252.5	*42*
Wagner, 1998	Austria	CGC based with ext. ref.	BRCA+, from CGC		FH-	No	Yes	x	x	x	>1970 (carriers) >1981(non-c)	34	NA	34	87	47	97	134	*34*	231	*39*
Wagner, 1998	Austria	CGC based with ext. ref.	BRCA+, from CGC		FH-	No	Yes	x			>1970 (carriers) >1981(non-c)	23	NA	68	87	47	97	134	*34*	231	*39*
Xu, 2012	China	Unselected cohort	Ancestry unclear (only A. Jewish mutations tested)			Yes	NA				1/1999–12/2005	52^b^	28^b^	280	87	84	61	171	*43*	232	*39*

*CGC = Clinical Genetic Centre; CGC based with ext*. *ref*. = *CGC based study with external reference group; CGC based with int*. *ref*. = *CGC based study with internal reference group; Unselected cohort = Unselected cohort study; FH = family history; NST = Neo-adjuvant systemic therapy; NA = not applicable;*

^*a*^ *Both carriers and non-carriers were identified in the CGC but because only a selection of the CGC population was included and matching was performed the study was defined as an” CGC based with ext*. *ref” type of study;*

^*b*^ *Only a selection of founder mutations was included*.

**Table 2:** Results of studies included in the review (N = 66).

Author + year	Mutation		Outcome				Unadjusted Risk estimates										Adjusted Risk estimates		
							5-year survival (%)				10-year survival (%)				Unadjusted HR	Survival difference in words^a^	Adjusted HR	Survival difference in words^a^	Adjustments for confounders
	B1	B2	OS	BCSS	RFS	MFS	‘non-carriers’	carriers	Diff-erence	F	‘non-carriers’	carriers	Diff-erence	F					
Ansquer, 1998	x		X				84	70	-14*	x									
	x				x											Worse			
Arun, 2011	x		X				90.5	86.8	-3.7										
	x				x		73.5	72.1	-1.4										
		x	X				90.5	100	9.5										
		x			x		73.5	92.9	19.4										
	x	x	X				82	85	3										
	x	x			x		65	71	6										
Bayraktar, 2011	x	x	X				85	93	8		74	74	0	x	0.52 (0.23–1.19)		0.51 (0.23–1.17)		Age at diagnosis (>40 vs < = 40) and Clinical stage (1–3)
	x	x			x		74	81	7		55	57	2	x	0.70 (0.40–1.23)		0.67 (0.38–1.19)		
Bonadona, 2007	x		X				89.6	93.3	3.7						0.67 (0.16–2.77)		0.29 (0.04–2.26)		Unclear, but probably: age at diagnosis, axilarry node status, grade, ER-status, PR-status, tumour size
	x			X			89.6	93.3	3.7						0.67 (0.16–2.77)		0.29 (0.04–2.26)		
	x				x		90	100	10	x									
	x					x	78.2	93.3	15.1						0.47 (0.12–1.94)		0.24 (0.03–1.82)		
	x	x	X				89.6	95	5.4						0.50 (0.12–2.07)				
	x	x		x			89.6	95	5.4						0.50 (0.12–2.07)				
	x	x			x		100	88.8	-11.2										
	x	x				x	78.2	94.7	16.5						0.37 (0.09–1.51)				
Brekelmans, 2007	x		X				75	69	-6		55	50	-5		1.01 (0.75–1.37)^c^		1.3 (0.91–1.85)		Age at diagnosis, stage, adjuvant treatment, ER-status, morphology, histological grade, B(s)O, occurrence of contralateral breast cancer
	x			x			78	73	-5		59	62	3		0.89 (0.63–1.25) ^c^		1.21 (0.83–1.76)		
	x				x		88	88	0		79	84	5		0.92 (0.56–1.51) ^c^		0.84 (0.41–1.75)		
	x					x	64	68	4		50	60	10		0.71 (0.52–0.96)* ^c^		1.25 (0.78–1.92)		
		x	X				75	75	0		55	61	6				1.07 (0.66–1.74)		
		x		x			78	80	2		59	68	9				0.84 (0.48–1.47)		
		x			x		88	83	-5		79	83	4				0.85 (0.26–2.77)		
		x				x	64	73	9		50	61	11				0.75 (0.44–1.29)		
Brekelmans, 2007	x		X				83	69	-14		66	50	-16						
	x			X			87	73	-14		70	62	-8						
	x				x		88	88	0		85	84	-1						
	x					x	73	68	-5		61	60	-1						
		x	X				83	75	-8		66	61	-5						
		x		x			87	80	-7		70	68	-2						
		x			x		88	83	-5		85	83	-2						
		x				x	73	73	0		61	61	0						
Budroni, 2009		x	x				91	81	-10						0.70 (0.46–1.37) ^d^		0·80 (0·48–1·62)		Tumour stage
Carroll, 2011	x	x	x				92	97.5	5.5										
Chappuis, 2000	x^b^	x^b^	x													Worse*		Worse	Age at diagnosis, tumour size, nuclear grade, LN-status, ER-status, p27kip expression
	x^b^	x^b^				x	82	58	-24*						2.7 (1.4–5.2) *		2.1 (1.0–4.3)*		
Chappuis, 2005	x^b^			x			82†	74†	-8	x	71†	61†	-10	x	1.9 (0.99–3.6)		0.8 (0.4–1.6)		Age at diagnosis, tumour size, nuclear grade, LN-status, ER-status, Cyclin E expression, p27kip expression
Chiappetta, 2010	x		x				94	72	-22*		83	68	-15*	x					
		x	x				94	92	-2		83	79	-4	x					
Cortesi, 2010	x		x				82	94	12*	x	73	77	4*				0.29 (0.13–0.62)*		Stage, ER-status, PR-status, grade, age at diagnosis, chemotherapy
	x				x		86	86	0	x	75	70	-5						
Cortesi, 2010	x		x				88	94	6	x	77	77	0						
	x				x		83	86	3	x	70	70	0						
Cortesi, 2010	x		x				90	96	6*	x	73	85	12*						
Eccles, 2001	x		x				82	81	-1	x	73	75	2	x					
	x				x		67	64	-3	x	56	55	-1	x					
Eccles, 2001	x		x				92	81	-11	x	81	75	-6	x					
	x				x		64	64	0	x	44	55	10	x					
Eerola, 2001	x		x				78	67	-11								1.3 (0.63–2.7)		Stage, age at diagnosis, calendar year of diagnosis, follow-up year, family history
		x	x				78	77	-1								0.78 (0.39–1.57)		
Eerola, 2001	x		x				86	67	-19										
		x	x				86	77	-9										
Einbeigi, 2001	x		x				80	85	5	x	65	70	5	x					
Ellberg, 2010	x	x	x												1.90 (0.99–3.65)^d^			Worse*	Age at diagnosis, tumour size, number LN+, occurrence of distant metastasis
El-Tamer, 2004	x^b^		x				91.3	90.7	-0.6		81	79.4	-1.6						
	x^b^			x			91.6	90.7	-0.9		84.6	79.4	-5.2						
	x^b^				x		92	72	-20*	x	91	72	-19*	x					
	x^b^					x										Equal			
		x^b^	x				91.3	94.7	3.4		81	94.7	13.7						
		x^b^		x			91.6	94.7	3.1		84.6	94.7	10.1						
		x^b^			x		92	83	-9	x									
		x^b^				x										Equal			
Foulkes, 1997	x^b^				x		95	80	-15										
Gaffney, 1998	x		x				69	75	6		50	65	15	x					
		x	x				69	73	4		50	50	0	x					
	x	x	x				69	75	5	x	50	55	5	x					
Gaffney, 1998	x		x				75	75	0	x	55	65	10	x					
		x	x				70	73	3	x	60	50	-10	x					
	x	x	x				70	75	5	x									
Goffin, 2003	x^b^		x				85	72	-13	x	75	57	-18	x	1.9 (0.99–3.6)		1.4 (0.7–2.9)		Tumour size, grade, LN-status, P53-expression
	x^b^					x	82†	62†	-20*	x					1.6 (0.9–2.9)		1.2 (0.7–2.4)		
Goffin, 2003	x^b^	x^b^		x											1.8 (0.96–3.2)		1.1 (0.6–2)		Tumour size, nuclear grade, ER-status, LN-status, P53 expression, glomeruloid microvascular proliferation
Gonzalez-Angulo, 2011	x	x	x				52.8	73.3	20.5								0.45 (0.16–1.29)		Stage, nuclear grade
	x	x			x		51.7	86.2	34.5*								0.17 (0.04–0.71)*		
Goode, 2002	x		x				85	42	-43*	x					4.14 (1.32–13)*		1.99 (0.47–8.45)		Grade, tumour type
		x	x				85	70	-15	x	77	70	-1	x					
Goodwin, 2012	x		x				89	86	-3	x	75	68	-7	x	1·43 (0·92–2·23)		0·99 (0·62–1·59)		Age at diagnosis, T-stage, nodal stage, grade, ER/PgR status, year of diagnosis
	x					x	86	82	-4	x	76	76	0	x	1.19 (0.74–1.89)		0.83 (0.51–1.35)		
		x	x				90	88	-2*	x	76	69	-7*	x	1.82 (1.15–2.86)*		1.12 (0.70–1.79)		
		x				x	86	75	-11*	x	79	73	-6*	x	1.63 (1.02–2.6)*		1.00 (0.62–1.61)		
Hagen, 2009	x		x				85	90	5	x	74	76	2	x					
Hamann, 2000	x		x				87.1	83.9	-3.2		81.3	71.7	-9.6						
	x				x		86.9	53.3	-33.6*		76	53.3	-22.7*						
Heikkinen, 2009	x			x			93	83	-10*	x	84	76	-8*	x	1.67 (0.99–2.82)			Equal	Grade, PR-status, HER2, T-status, N-status, M-status
		x		x			93	87	-6*	x	84	63.7	-20.3*	x	2.34 (1.5–3.66)*		2.06 (1.03–4.15)*		
Huzarski, 2013	x^b^		x				89	88	-1	x	82.2	80.9	-1.3		1.13 (0.83–1.57		1.81 (1.26–2.61)*		year of birth, age at diagnosis, ER status, PR status, HER2 status, Size, Nodal status, Oophorectomy (time-varying), tamoxifen, chemotherapy
Jóhannsson, 1998	x		x				68	68	0	x	45	57	12	x	1.5 (0.9–2.4) ^d^				
Jóhannsson, 1998	x		x				80	62	-18	x	62	56	-6	x	1.5 (0.6–3.7)				
Kirova, 2010	x	x			x		90	86	-4	x	70	65	-5	x					
Kirova, 2010	x	x	x				98	92	-6	x	82	76	-6	x					
	x	x			x		82	86	4	x	77	65	-12	x					
Kirova, 2010	x	x	x				92	92	0	x	90	76	-14	x					
	x	x			x		89	86	-3*	x	79	65	-14*	x	1.8 (1–3.3)*				
Lee, 1999	x		x				78	79	1										
		x	x				78	65	-13										
	x	x	x				78	74	-4		61	61	0	x	1.04 (0.7–1.55) ^d^				
Lee, 2011	x		x				73.9	82.1	8.2						0.58 (0.25–1.25)		0.73		Age at diagnosis, stage
	x			x			73.9	82.1	8.2						0.58 (0.25–1.25)		0.73		
	x				x		80.2	89.6	9.4										
	x					x	69.9	75.6	5.7						0.79 (0.38–1.58)		0.9		
Loman, 2000		x	x				84	72	-12	x	70	58	-12	x	1.6 (0.98–2.7)				Stage
		x		x			90	76	-14*	x	79	59	-20*	x	2 (1.2–3.4)*		1.6 (0.85–3.1)		
Moller, 2002	x				x		96	75	-21									Equal	Grade, ER-status
Moller, 2007	x		x				92	73	-19*		86	52	-34*	x					
		x	x				92	96	4		86	96	10	x					
Musolino, 2007	x	x	x				93	93	0	x	77	82	5	x					
	x	x			x		86	78	-8	x	81	78	-3	x					
Musolino, 2007	x	x	x				100	93	-7	x	100	82	-18	x					
	x	x			x		94	78	-16	x	81	78	-3	x					
Nisman, 2010	x^b^	x^b^			x		77.8	89.7	11.9									Equal	Stage, serum TK1 activity, presence of necrosis, vascular invasion, tumour grade, ER-status, PR-status
Pierce, 2000	x	x	x				91	86	-5						1.18				
	x	x		x			91	92	1						0.71				
	x	x			x		80	78	-2						1.36				
Pierce, 2006	x	x			x		95	95	0	x	91	88	-3		1.37		1.37 (0.77–2.42)		Age at diagnosis, stage, margins, tamoxifen, chemotherapy
Plakhins, 2011	x^b^		x				Only survival % of separate mutations. Therefore, not taken into account here.										1.1 (0.81–1.48)		Tumour size (<5cm vs >5cm), axillary node status (neg vs pos), age at diagnosis (<50 vs >50)
Plakhins, 2013	x^b^		x				82.02	84.47	2.45		72.36	73.9	1.54						
	x^b^			x							79.34	80.15	0.81						
Rennert, 2007	x^b^		x								51	49	-2		1.09 (0.79–1.51)		1.13 (0.78–1.66)		Age at diagnosis, tumour size, LN-status, M-status
	x^b^			x							67	67	0		1.08 (0.72–1.63)		0.76 (0.45–1.3)		
		x^b^	x								51	48	-3		1.07 (0.73–1.58)		1.2 (0.77–1.86)		
		x^b^		x							67	56	-11		1.42 (0.92–2.19)		1.31 (0.8–2.15)		
Rijnsburger, 2010	x	x	x				100	92.7	-7.3										
	x	x				x	100	83.9	-16.1										
Robson, 1998	x^b^	x^b^	x													Equal			Stage, axillary node status
	x^b^	x^b^			x		69	65	-4									Equal	
Robson, 1999	x^b^		x								80.6	63.3	-17*						LN-status / tumour stage, age at diagnosis (only for BCSS)
	x^b^			x							87.2	67.3	-19.9*						
	x^b^					x					83.9	58.3	-25.6*				1.7 (0.66–4.36)		
	x^b^	x^b^	x				83	82	-1*		80.6	66	-14.6*						
	x^b^	x^b^		x			95.9	85.3	-10.6*		87.2	71.9	-15.3*				2.08 (0.79–5.44)		
	x^b^	x^b^			x		95.5	85.1	-10.4		93.1	78	-15.1		1.79 (0.64–5.03)				
	x^b^	x^b^				x	90.5	74.1	-16.4*		84.3	66.3	-18.1*				1.45 (0.6–3.49)		
Robson, 2004	x^b^			x			92	80	-12*	x	86	62	-24*				2.39 (1.2–4.75) *		Age at diagnosis, tumour size, axillary node status
		x^b^		x							86	84.5	-1.5					Equal	
	x^b^	x^b^		x							86	67	-19*						
	x^b^	x^b^			x		96	92	-4	x	92	88	-4						
Seynaeve, 2004	x		x														1.76 (0.72–4.3)		Age at diagnosis, tumour size
Soumittra, 2009	x	x	x				78	75	-3	x									
	x	x			x		72	75	3	x									
Stoppa-Lyonnet, 2000	x		x				85	49	-36*						5.1*		3.5 (1.3–9.7) *		Nodal status, ER-status (only for MFS)
	x			x												Worse*		Worse*	
	x				x		79	54	-25										
	x					x	84	18	-66*						3.5*		2.6 (1–6.5) *		
Tryggvadottir, 2013		x^b^		x			85	80	-5*	x	72	53	-19*		1.61 (1.32–1.96)^d^		0.98 (0.64–1.48)		year of birth, year of diagnosis, tumour size, nodal status, grade, ER status, diploidy
Verhoog, 1998	x		x				69	63	-6		46	53	6	x	1.04 (0.63–1.71)		1.21 (0.72–2.04)		Tumour stage
	x			x			71	64	-7										
	x				x		51	49	-2						1 (0.65–1.55)		1.09 (0.7–1.7)		
Verhoog, 1999		x	x				75	74	-1						0.75 (0.37–1.51)		0.59 (0.27–1.29)		Stage
		x		x			76	77	1										
		x			x		52	52	0						0.92 (0.52–1.62)		0.84 (0.44–1.63)		
Veronesi, 2005	x	x	x				90	100	10	x	90	88	-2				1.1 (0.3–4.9)		Age at diagnosis, grade
	x	x			x		81	74	-5	x	60	51	-9				0.9 (0.2–5.3)		
Vinodkumar, 2007	x		x				No survival % because KM-figure of bad quality.								3.7*				
Wagner, 1998		x	x													Equal			
		x			x											Equal			
Wagner, 1998	x		x													Equal			
	x				x											Equal			
Xu, 2011	x^b^			x			85	87	2							Equal			
		x^b^		x			85	91	6										

*OS = overall survival; BCSS = breast cancer-specific survival; RFS = recurrence-free survival; MFS = metastasis-free survival; F = 5/10-year survival (%) was estimated from the Kaplan-meier figure published in the paper;*

*Significant result;

^*a*^ *When no risk estimates were reported but analyses were clearly done and the difference in survival was mentioned in the article*, *then the difference is described here;*

^*b*^ *Only a selection of founder mutations was included;*

^*c*^ *Risk estimate from other publication on the same study population (Brekelmans (2007)*: *extra risk estimates from Brekelmans (2006); Chappuis (2005)*: *extra risk estimates from Foulkes (2004); Goffin (2003)*: *extra risk estimates from Chappuis (2000));*

^*d*^ *Adjusted for age and/or calendar year of diagnosis*.

We performed a systematic review of all studies published reporting overall survival and/or breast cancer-specific survival and/or metastasis-free survival and/or recurrence-free survival related to *BRCA* mutation carriership. We systematically reviewed important differences in design between the studies and assessed their methodological rigor using a specially developed scoring-system aiming to give the best evidence regarding the prognosis of *BRCA1*- and *BRCA2*-associated tumours. We explored whether these differences could explain the discrepancies in outcomes between the studies. Because clinico-pathological features of the tumour are important prognostic factors and *BRCA1*-associated breast cancers are known to differ in this respect from tumours not associated with *BRCA* mutations, we paid special attention to a possible role for these factors as confounders or mediators in the association between *BRCA1* and *BRCA2* mutation carriership and breast cancer survival.

## Materials and Methods

### Search strategy and selection of relevant literature

**Fig 1:**
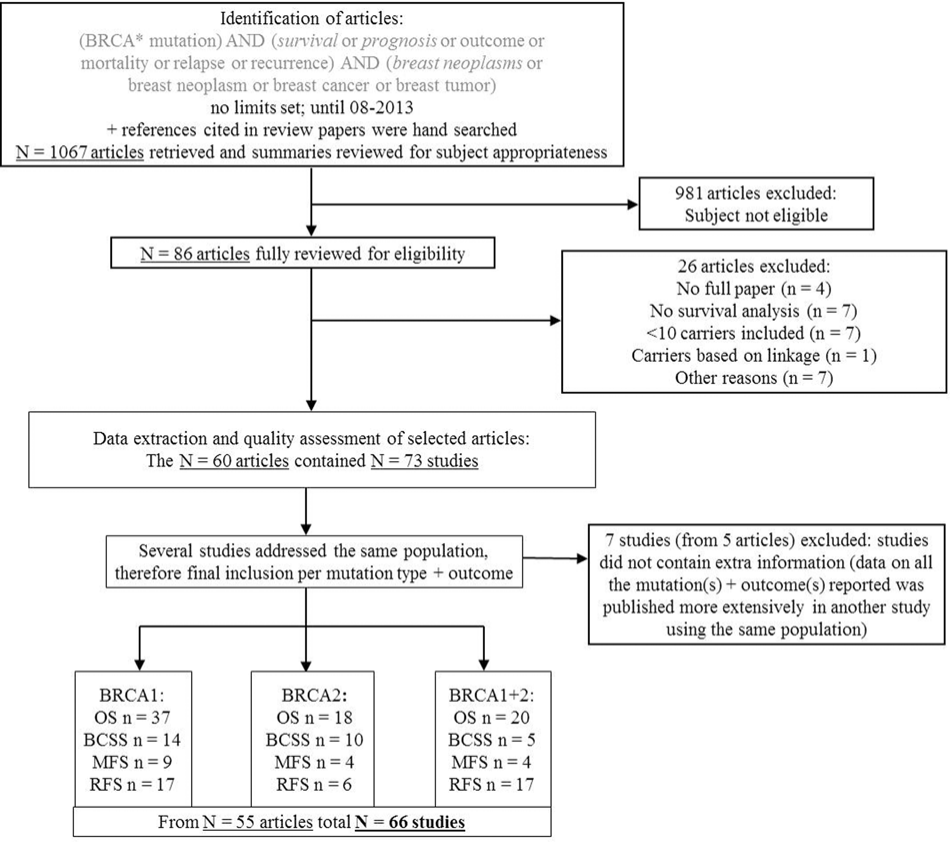
Flow diagram of the inclusion process of papers and studies in the review. OS = Overall survival; BCSS = Breast cancer-specific survival; MFS = Metastasis-free survival; RFS = Recurrence-free survival.

Studies were identified through a systematic search in Pubmed until August 2013 with no language restrictions using the following terms as free text terms and available MeSH terms, shown in *italics*; ‘(BRCA* mutation) AND (*survival* or *prognosis* or outcome or mortality or relapse or recurrence) AND (*breast neoplasms* or breast neoplasm or breast cancer or breast tumour)’; no limits were set (Fig. 1). References cited in relevant review papers were hand-searched for additional papers.

One reviewer (AJvdB) browsed the title and abstract of the papers for their eligibility for the topic of research; i.e. the association between *BRCA1* and/or *BRCA2* mutation carriership and breast cancer survival. After this first selection, two reviewers (AJvdB and MKS) independently selected papers based on the following criteria: studies should be original reports and *BRCA1/2* mutation status should be known; we accepted studies in which less than 50% of the carrier group was identified by linkage (identification of individuals with a high probability of having a *BRCA* mutation by determination of disease patterns in high-risk families, possibly combined by identifying genetic markers that are co-inherited with the disease [5]) instead of by testing. In addition, studies should have included at least ten carriers of a *BRCA1* and/or *BRCA2* mutation, and outcomes reported should include overall survival and/or breast cancer-specific survival and/or metastasis-free survival and/or recurrence-free survival. To allow comparison between as many studies as possible, we focussed on 5- and 10-year survival estimates. When multiple studies using the same study population had been published, the study with the largest number of subjects and longest follow-up time was included. If studies used the same study population but reported different mutations and/or outcomes, each mutation type and outcome combination was included separately (Fig. 1). Disagreement on the inclusion of one paper was solved by consensus.

### Quality scoring system

Because no specific quality assessment scoring system was available for this research topic, we developed a scoring system (S1 Supporting Information, part A) including general methodological aspects as well as specific aspects of studies examining the association between *BRCA1/2* mutation carriership and breast cancer survival, following the method of Monninkhof and colleagues [6]. The potential forms of bias were categorized into three main types: selection bias, misclassification bias and confounding/accounting for mediating variables, contributing at most 300 points, 100 points and 200 points, respectively, to the quality scoring, representing the relative weights of 3:1:2 (additional information: S1 Supporting Information, part B). For each paper a quality score from 0 (potential for having extensive bias) to maximum 600 (less bias potential) could be assigned. When considering unadjusted survival outcomes, the scores for confounding/accounting for mediating variables (= 200 points) were excluded and a maximum score of 400 could be attained. Survival outcomes without adjustment or with adjustment for age or year at diagnosis alone in the analysis were considered unadjusted outcomes; survival outcomes adjusted for tumour characteristics and/or treatment in the analysis were considered adjusted outcomes (one exception to this were outcomes from studies where matching on tumour characteristics was performed (n = 7 [7–13]); these outcomes were included as unadjusted since in five of these studies [8,10–13] only absolute survival differences were reported).

Two reviewers (AJvdB and MKS) independently assessed study quality for each included paper. Scores were compared thereafter and disagreements were solved by consensus or consultation of a third reviewer (FEvL).

### Study classification

All studies were categorized according to quality into two groups; studies achieving at least 50% of the maximum score (high quality (HQ) studies) and studies achieving less than 50%. This arbitrary cut-off was chosen upfront with the rationale to prevent studies with a high potential for bias to contribute to the evidence. However, sensitivity analyses were performed including all studies.

Furthermore, studies were classified into three types based on the method of patient inclusion: studies that included *BRCA1/2* mutation carriers mostly from a clinical genetic centre (CGC), and compared them with an external comparison group of non-carriers, or so called ‘non-carriers’ who were in fact untested patients assumed to be largely non-carriers, from the population or hospital (further referred to as ‘CGC based studies with external reference group’); studies that included both tested carriers and confirmed non-carriers from the CGC (‘CGC based studies with internal reference group’); and studies that tested a group of breast cancer patients from the hospital or general population, unselected for family history, for *BRCA1/2* mutation carriership (‘Unselected cohort studies’).

### Data representation and analyses

All data were taken from the papers; no attempt was made to request individual data from the researchers. All analyses were performed separately for the different *BRCA* mutations, stratified for all different survival outcomes. Significance testing was not used in the analyses, except in the standard meta-analyses on studies which reported hazard ratios.

A best-evidence synthesis tool (S2 Supporting Information; developed by Monninkhof and colleagues [6], adapted by the authors for this review) was used to score the evidence, taking into account the study quality and consistency of the results. Here only the HQ studies, with at least 50% of the attainable quality score, were considered. According to our criteria, at least four HQ studies were needed to generate sufficient evidence. Specific classification of the evidence is shown in S2 Supporting Information. For the best-evidence synthesis, a better survival for *BRCA1/2* carriers compared to ‘non-carriers’ was arbitrarily defined as an absolute survival difference ≥10% or a risk estimate ≤0.88; a worse survival as an absolute survival difference ≥10% or a risk estimate ≥1.14; no association as an absolute survival difference <10% and a risk estimate between 0.88 and 1.14. These cut-offs were chosen arbitrarily considering a difference of 10% to certainly be of clinical relevance, and with the rationale that the methods used were not sensitive enough to detect smaller differences. In the sensitivity analysis also other cut-offs were used.

The best-evidence synthesis was performed irrespective of statistical significance (S2 Supporting Information). Sensitivity analyses were performed using all studies (irrespective of study quality), using only ‘unselected cohort studies’, using only significant results (*P* < 0.05), and using different cut-offs of the definition of better and worse survival for carriers compared to ‘non-carriers’ without consideration of statistical significance of individual studies (S9 Supporting Information).

To estimate the average effect-size in the best-evidence synthesis, meta-analyses were performed using the HQ studies; this was only done for the mutation and outcome combinations where sufficient evidence, i.e. >4 HQ studies, was available. For the absolute survival differences, pooled estimates were calculated using weighting based on the number of included *BRCA1* or *BRCA2* mutation carriers per study (weight per study (%) = (n of carriers in that specific study / total n of carriers of all studies which are used to form the pooled estimate)*100). In most papers 95% confidence intervals, standard errors or standard deviation of absolute survival differences were not reported hence these could not be taken into account. Statistical heterogeneity was based on subjective indications using the forest plots. For the hazard ratios (HR), pooled estimates were calculated and statistical heterogeneity was assessed using Random effect analyses, which is designed to estimate the mean effect size from a range of studies while accounting for heterogeneity across the studies [14].

To examine whether the heterogeneity between the results could be explained by different aspects of the study quality, risk estimates and quality scores per bias of all studies were graphically displayed. Funnel plots were used to investigate possible publication bias [15]. Statistical analyses were performed using STATA-11.2.

## Results

Until August 2013, 1067 papers were identified in the Pubmed database, of which 66 studies from 55 papers matched the inclusion criteria and contributed data (Fig. 1).

The main characteristics and results of the 66 included studies [7–13,16–63] are shown in Table 1 and Table 2 respectively. All studies were published after 1997; the numbers of included carriers ranged from 10 to 233. Of these 66 studies, 12 studies [22,23,28,30–32,39,45,49,51–53] were performed in an Ashkenazi Jewish study population and tested only the three founder mutations.

Most studies (n = 25) compared *BRCA1/2* mutation carriers with an external ‘non-carrier’ group: ‘CGC based studies with external reference group’; 18 were ‘CGC based studies with internal reference group’ and 23 were ‘Unselected cohort studies’ (Table 1).

**Fig 2:**
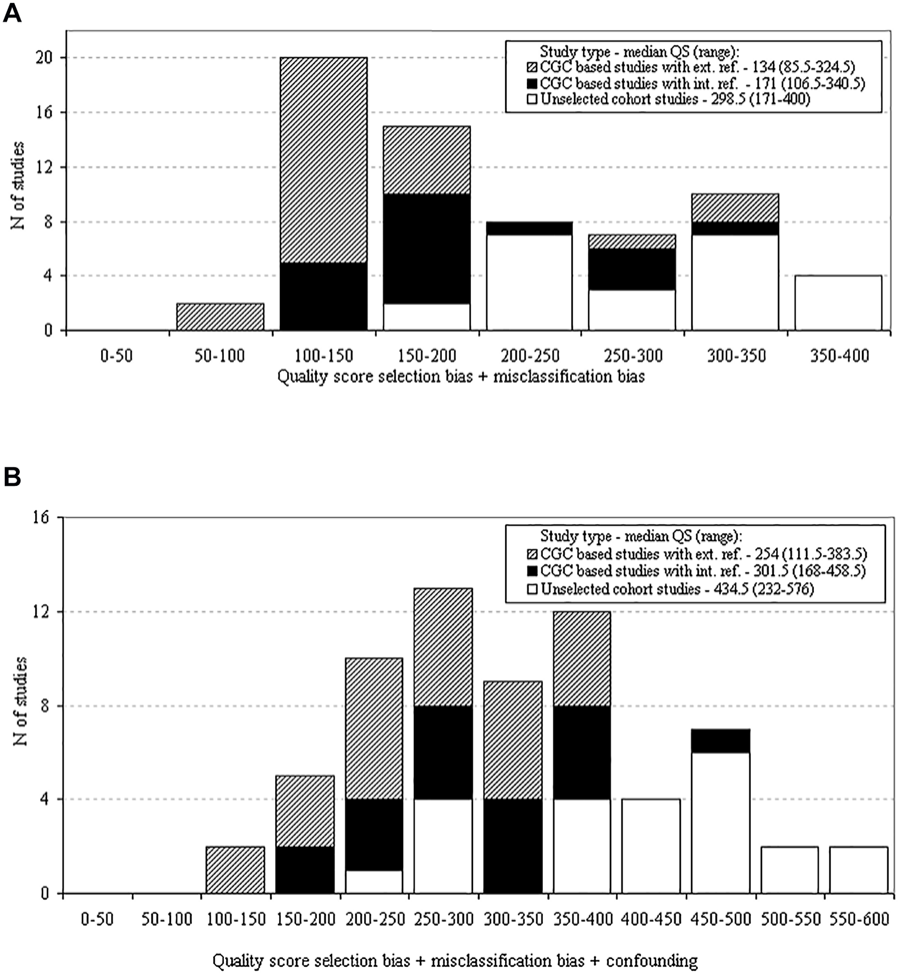
Quality distribution based on selection bias, misclassification bias and confounding/accounting for mediating variables in all included studies (n = 66). The scores for selection bias and misclassification bias were taken into account for the analysis of the univariate outcomes (panel A). The scores for selection bias, misclassification bias and confounding accounting for mediating variables were taken into account for the analysis of multivariate outcomes (panel B). *CGC based studies with ext*. *ref*. = *CGC based studies with external reference group; CGC based studies with int*. *ref*. = *CGC based studies with internal reference group*.

When considering unadjusted outcomes and only taking into account selection and misclassification bias in the analysis, the quality scores of the included studies ranged from 85.5 (21% of maximum) to 400 (100% of maximum); 29 studies (44%) were considered HQ with scores >200 (Fig. 2A and Table 1). When taking into account all three bias categories for the analyses of adjusted survival outcomes, the quality scores ranged from 111.5 (18.6% of maximum) to 576 (86% of maximum); 36 studies (55%) were considered HQ with scores >300 (Fig. 2B and Table 1). For both unadjusted and adjusted outcomes the ‘Unselected cohort studies’ had the highest scores (*P* <0.001 and 0.001, respectively; Fig. 2A and B).

S3 Supporting Information shows the number of studies reporting risk estimates for the specific outcomes per mutation type. The mutation types and outcomes reported per study varied greatly; only for 15 risk estimates out of 48, more than four HQ studies were available.

### *BRCA1* and *BRCA2* mutation carriership and survival

In the following paragraphs we provided summaries of the results of the different survival outcomes for *BRCA1* and *BRCA2* carriers. Extensive descriptions of the reported results are available in the Supporting information as indicated.

#### *BRCA1* mutation carriership and overall survival.

**Fig 3:**
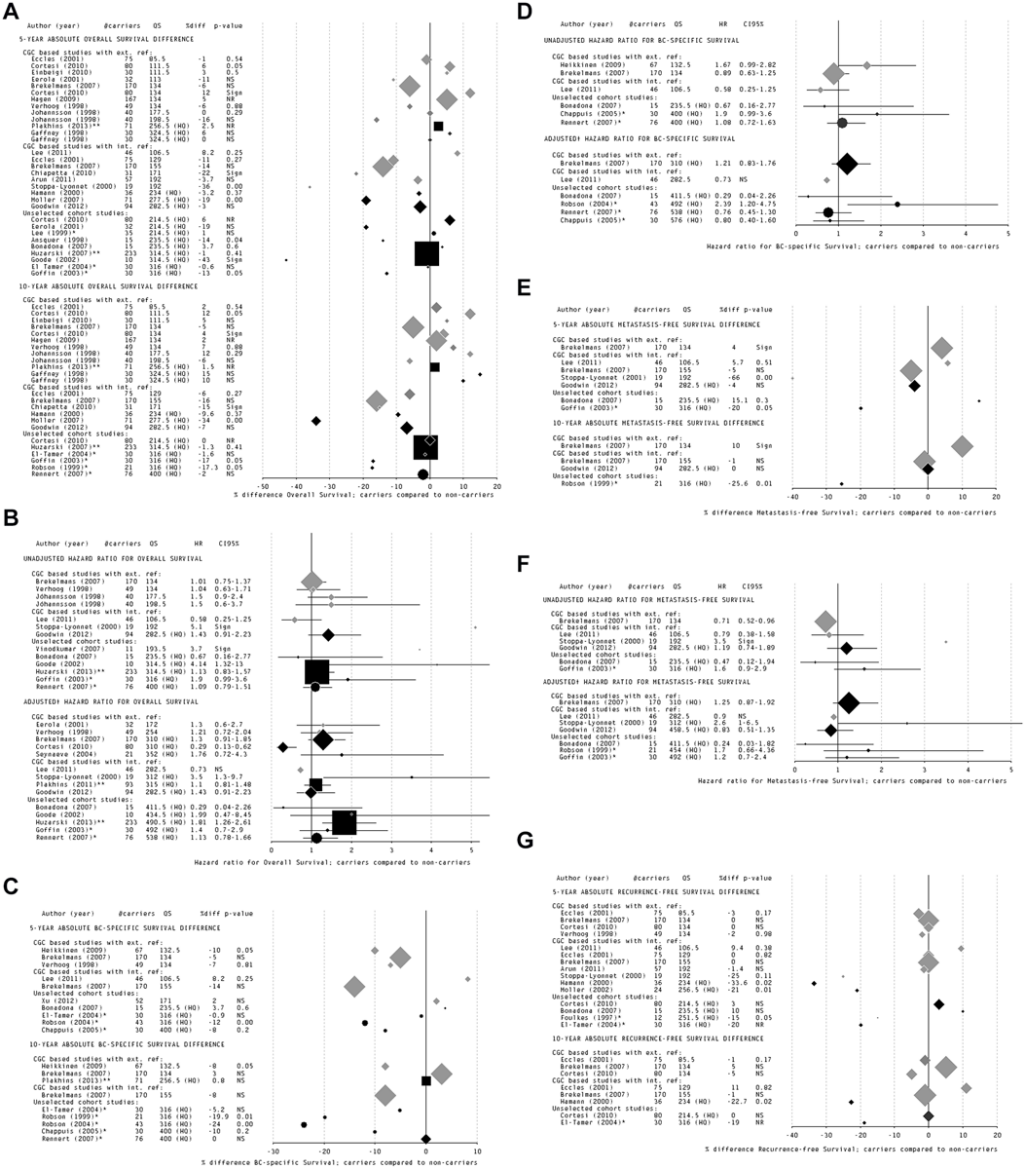
Forest plots of studies reporting survival estimates for *BRCA1* mutation carriers compared to ‘non-carriers’, classified per study type and sorted by quality score. Separate forest plots are shown of studies reporting overall survival (panels A and B), breast cancer-specific survival (panels C and D), metastasis-free survival (panels E and F) and recurrence-free survival (panel G) of *BRCA1* mutation carriers compared to ‘non-carriers’. Additionally, the results for each type of survival outcome are stratified per reported risk estimate: the 5-year and 10-year absolute overall survival difference (panels A, C, E, G) and the adjusted and unadjusted hazard ratios for overall survival (panels B, D, F). Size of the bullet represents the number of included carriers; black bullet = HQ study; round bullet (●) and * = A. *Jewish study population*, only founder mutations tested; square bullet (■) and ** = specific study population (but not A. *Jewish)*, *in which only founder mutations were tested;— = 95% Confidence interval (only for hazard ratios); CGC based studies with ext*. *ref*. = *CGC based studies with external reference group; CGC based studies with int*. *ref*. = *CGC based studies with internal reference group; Sign = statistically significant (P < 0*.*05); NS = not statistically significant; NR = not reported; †Adjusted for clinico-pathological characteristics and/or treatment*.

The forest plots of absolute survival differences in Fig. 3A and HRs in Fig. 3B showed inconsistent results for both the HQ studies as well the other studies (S4 Supporting Information, part A). Nevertheless, all unadjusted pooled estimates showed a worse survival for *BRCA1* mutation carriers, though effects were small: pooled 10-year absolute survival difference 4.9%; pooled HR 1.17 (95% CI 0.93–1.40) (Table 3 and S6 Supporting Information, panel A). Also the pooled estimate of the adjusted HR of 1.14 (95% CI 0.73–1.55) indicated a small survival disadvantage for *BRCA1* mutation carriers, but the heterogeneity test showed a large inconsistency between the results reported (Table 3 and S6 Supporting Information, panel B). Using the best-evidence synthesis, we concluded that there is still indecisive evidence for an association between *BRCA1* mutation carriership and unadjusted/adjusted overall survival of breast cancer patients (Table 4).

**Table 3:** Pooled estimates and heterogeneity analysis for separate risk estimates.

						Heterogeneity analysis	
Type of survival		Type of outcome	N of HQ studies	Pooled estimate	95% CI	Chi square statistic^c^	p-value^c^
***BRCA1* mutation carriers compared to ‘non-carriers’**							
Overall	Unadjusted	5-year absolute survival difference (%)^*a*^	15	-3.3	NA	NA	NA
		10-year absolute survival difference (%)^*a*^	12	-4.9	NA	NA	NA
		Hazard ratio^*b*^	6	1.17	0.93–1.40	3.59	0.61
	Adjusted	Hazard ratio^*b*^	11	1.14	0.73–1.55	42.79	<0.001
BC-specific	Unadjusted	5-year absolute survival difference (%)^*a*^	4	-6.2	NA	NA	NA
		10-year absolute survival difference (%)^*a*^	6	-6.8	NA	NA	NA
		Hazard ratio^*b*^	3	1.12	0.71–1.53	1.86	0.40
	Adjusted	Hazard ratio^*b*^	5	0.92	0.58–1.26	6.18	0.19
Metastasis-free	Unadjusted	5-year absolute survival difference (%)^*a*^	3	-5.4	NA	NA	NA
		10-year absolute survival difference (%)^*a*^	2	-4.7	NA	NA	NA
		Hazard ratio^*b*^	3	1.09	0.54–1.65	2.90	0.24
	Adjusted	Hazard ratio^*b*^	6	0.99	0.63–1.43	6.30	0.28
Recurrence-free	Unadjusted	5-year absolute survival difference (%)^*a*^	6	-10.7	NA	NA	NA
		10-year absolute survival difference (%)^*a*^	3	-9.5	NA	NA	NA
		Hazard ratio^*b*^	No HQ studies available				
***BRCA2* mutation carriers compared to ‘non-carriers’**							
Overall	Unadjusted	5-year absolute survival difference (%)^*a*^	9	-4.4	NA	NA	NA
		10-year absolute survival difference (%)^*a*^	7	-2	NA	NA	NA
		Hazard ratio^*b*^	3	1.09	0.58–1.59	5.22	0.07
BC-specific	Unadjusted	5-year absolute survival difference (%)^*a*^	2	-4.3	NA	NA	NA
		10-year absolute survival difference (%)^*a*^	4	-14.8	NA	NA	NA
		Hazard ratio^*b*^	2	1.57	1.29–1.86	0.27	0.60

The risk estimates which are shown are from outcomes for which more than four high quality studies were available and evidence could be formed using the best-evidence synthesis (Table 4 and Table 5). Only high quality (HQ) studies are considered.

^*a*^ *No heterogeneity analysis performed*. Pooling weighted on the number of included BRCA1 or BRCA2 mutation carriers ((weight per study (%) = (n of carriers in that specific study / total n of carriers of all studies which are used to form the pooled estimate)*100));

^*b*^ *Random effect (DerSimonian and Laird) analyses performed;*

^*c*^ *Results of the heterogeneity test of the random effect (DerSimonian and Laird) analyses*.

**Table 4:** Best-evidence synthesis: a summary of the available evidence for the relation between *BRCA1* mutation carriership and breast cancer prognosis.

Type of survival	Unadjusted/ adjusted^a^	Studies reporting a worse survival^b^ % (n / total n)		Studies reporting a better survival^c^ % (n / total n)		Evidence^d^ (based on all studies)	Evidence^d^ (based on HQ studies)
		Low quality	High quality	Low quality	High quality		
Overall	Unadjusted	47 (8/17)	**41 (7/17)**	18 (3/17)	**18 (3/17)**	Indecisive	**Indecisive**
	Adjusted	67 (2/3)	**55 (6/11)**	33 (1/3)	**18 (2/11)**	Indecisive	**Indecisive**
BC-specific	Unadjusted	33 (2/6)	**43 (3/7)**	17 (1/6)	**14 (1/7)**	Nil	**Indecisive**
	Adjusted	0 (0/1)	**40 (2/5)**	100 (1/1)	**60 (3/5)**	Nil	**Indecisive**
Metastasis-free	Unadjusted	25 (1/4)	**75 (3/4)**	50 (2/4)	**25 (1/4)**	Indecisive	**Indecisive**
	Adjusted	0 (0/1)	**67 (4/6)**	0 (0/1)	**33 (2/6)**	Indecisive	**Indecisive**
Recurrence-free	Unadjusted	11 (1/9)	**67 (4/6)**	11 (1/9)	**17 (1/6)**	Nil	**Moderate**
	Adjusted	0 (0/1)	0 (0/1)	0 (0/1)	100 (1/1)	Indecisive*	Indecisive*

Studies are taken into account reporting the 5-year absolute survival and/or 10-year absolute survival and/or unadjusted hazard ratio (for univariate outcomes) or reporting a multivariate hazard ratio (for multivariate outcomes).

^*a*^ *Adjusted survival is based on risk estimates adjusted for clinico-pathological characteristics and/or treatment;*

^*b*^ *Worse survival for univariate (unadjusted) outcomes*: *unadjusted HR > = 1*.*14 or 5-year absolute survival difference > = 10% or 10-year absolute survival difference > = 10% (when the 5 and 10 year survival differences go in opposite directions*, *we decided there was no difference in survival)*. *Worse survival for multivariate (adjusted) outcomes*: *adjusted HR > = 1*.*14;*

^*c*^ *Better survival for univariate (unadjusted) outcomes*: *unadjusted HR < = 0*.*88 or 5-year absolute survival difference > = 10% or 10-year absolute survival difference > = 10% (when the 5 and 10 year survival differences go in opposite directions*, *we decided there was no difference in survival)*. *Better survival for multivariate (adjusted) outcomes*: *adjusted HR < = 0*.*88;*

^*d*^ *See appendix p 3 (Best-evidence synthesis)*. *Strong evidence*: *more than 75% of the HQ studies reported a worse survival; moderate evidence*: *60–75% of the HQ studies reported a worse survival and less than 25% of the HQ studies reported a better survival / 50–60% of the HQ studies reported a worse survival and less than 10% of the HQ studies reported a better survival; nil evidence*: *more than 60% of the HQ studies reported a better survival or no association / more than 40% of the HQ studies reported a better survival;*

*indecisive e evidence*: all other options / less than four HQ studies available (*).

**Table 5:** Best-evidence synthesis: a summary of the available evidence for the relation between *BRCA2* mutation carriership and breast cancer prognosis.

Type of survival	Unadjusted/ adjusted^a^	Studies reporting a worse survival^b^ % (n / total n)		Studies reporting a better survival^c^ % (n / total n)		Evidence^d^ (based on all studies)	Evidence^d^ (based on HQ studies)
		Low quality	High quality	Low quality	High quality		
Overall	Unadjusted	14 (1/7)	**50 (5/10)**	14 (1/7)	**20 (2/10)**	Nil	**Indecisive**
	Adjusted	0 (0/3)	33 (1/3)	100 (3/3)	0 (0/3)	Nil	Indecisive*
BC-specific	Unadjusted	33 (2/6)	**50 (2/4)**	0 (0/6)	**25 (1/4)**	Indecisive	**Indecisive**
	Adjusted	100 (2/2)	33 (1/3)	0 (0/2)	33 (1/3)	Indecisive	Indecisive*
Metastasis-free	Unadjusted	0 (0/2)	100 (1/1)	50 (1/2)	0 (0/1)	Indecisive*	Indecisive*
	Adjusted	NA	0 (0/2)	NA	50 (1/2)	Indecisive*	Indecisive*
Recurrence-free	Unadjusted	0 (0/4)	0 (0/1)	25 (1/4)	0 (0/1)	Nil	Indecisive*
	Adjusted	0 (0/1)	0 (0/1)	100 (1/1)	100 (1/1)	Indecisive*	Indecisive*

Studies are taken into account reporting the 5-year absolute survival and/or 10-year absolute survival and/or unadjusted hazard ratio (for univariate outcomes) or reporting a multivariate hazard ratio (for multivariate outcomes).

^*a*^ *Adjusted survival is based on risk estimates adjusted for clinico-pathological characteristics and/or treatment;*

^*b*^ *Worse survival for univariate (unadjusted) outcomes*: *unadjusted HR > = 1*.*14 or 5-year absolute survival difference > = 10% or 10-year absolute survival difference > = 10% (when the 5 and 10 year survival differences go in opposite directions*, *we decided there was no difference in survival)*. *Worse survival for multivariate (adjusted) outcomes*: *adjusted HR > = 1*.*14;*

^*c*^ *Better survival for univariate (unadjusted) outcomes*: *unadjusted HR < = 0*.*88 or 5-year absolute survival difference > = 10% or 10-year absolute survival difference > = 10% (when the 5 and 10 year survival differences go in opposite directions*, *we decided there was no difference in survival)*. *Better survival for multivariate (adjusted) outcomes*: *adjusted HR < = 0*.*88;*

^*d*^ *See appendix p 3 (Best-evidence synthesis)*. *Strong evidence*: *more than 75% of the HQ studies reported a worse survival; moderate evidence*: *60–75% of the HQ studies reported a worse survival and less than 25% of the HQ studies reported a better survival / 50–60% of the HQ studies reported a worse survival and less than 10% of the HQ studies reported a better survival; nil evidence*: *more than 60% of the HQ studies reported a better survival or no association / more than 40% of the HQ studies reported a better survival;*

*indecisive e evidence*: all other options / less than four HQ studies available (*).

#### *BRCA1* mutation carriership and breast cancer-specific survival

The forest plots in Fig. 3C (absolute survival differences) and Fig. 3D (HRs) seemed to point to a worse unadjusted breast cancer-specific survival for *BRCA1* compared to ‘non-carriers’, especially when looking at the HQ studies, although these effects were generally small (S4 Supporting Information, part B). The pooled breast cancer-specific survival estimates were a 10-year absolute worse difference of 6.8% and a HR of 1.12 (95% CI 0.71–1.53); in contrast, the adjusted HR showed a slightly better breast cancer-specific survival for *BRCA1* mutation carriers (0.92, 95% CI 0.58–1.36). None of the pooled estimates were statistically significant (Table 3 and S6 Supporting Information, panels C and D). Using the best-evidence synthesis, we concluded there is indecisive evidence for an association between *BRCA1* mutation carriership and unadjusted/adjusted breast cancer-specific survival (Table 4).

#### *BRCA1* mutation carriership and metastasis-free survival

The forest plots of absolute survival differences in Fig. 3E and HRs in Fig. 3F showed inconsistent results for both the HQ and other studies (S4 Supporting Information, part C). The pooled estimates showed a small unadjusted metastasis-free survival difference for *BRCA1* compared to the ‘non-carriers’: around 5% worse survival and a pooled HR of 1.09 (95% CI 0.54–1.65); while the pooled adjusted HR was 0.99 (95% CI 0.63–1.43) (Table 3 and S6 Supporting Information, panels E and F). Due to the inconsistency in the results, the best-evidence synthesis showed there is indecisive evidence for a conclusion about the association between *BRCA1* carriership and metastasis-free survival (Table 4).

#### *BRCA1* mutation carriership and recurrence-free survival

Most of the studies, certainly when considering the HQ studies, reported a worse unadjusted absolute recurrence-free survival for *BRCA1* mutation carriers compared to ‘non-carriers’ (forest plot: Fig. 3G; S4 Supporting Information, part D). This worse survival was supported by pooling of the study results: 10% absolute survival difference between the *BRCA1* and ‘non-carriers’ (Table 3). The best-evidence synthesis also showed there was moderate evidence for a worse unadjusted recurrence-free survival for *BRCA1* compared to ‘non-carriers’ (Table 4). Adjusted HRs for recurrence-free survival were only reported in two studies (S4 Supporting Information, part D) and no conclusions could be drawn.

#### *BRCA2* mutation carriership and overall survival

**Fig 4:**
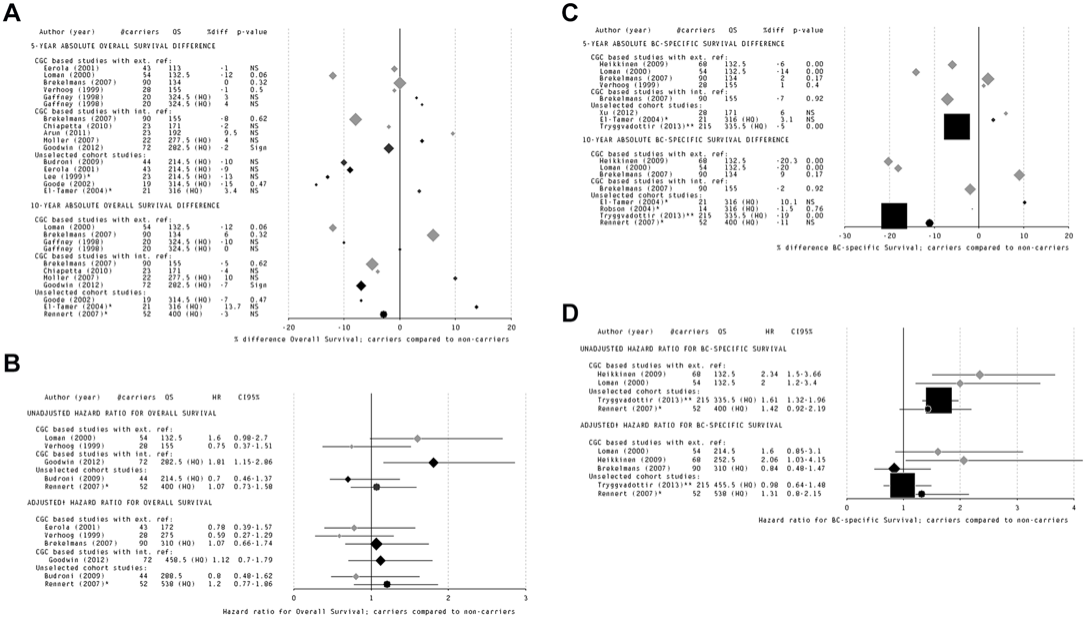
Forest plots of studies reporting survival estimates for *BRCA2* mutation carriers compared to ‘non-carriers’, classified per study type and sorted by quality score. Separate forest plots are shown of studies reporting overall survival (panels A and B), breast cancer-specific survival (panels C and D) of *BRCA2* mutation carriers compared to ‘non-carriers’. Additionally, the results for each type of survival outcome are stratified per reported risk estimate: the 5-year and 10-year absolute overall survival difference (panels A and C) and the adjusted and unadjusted hazard ratios for overall survival (panels B and D). Size of the bullet represents the number of included carriers; black bullet = HQ study; round bullet (●) and * = A. *Jewish study population*, only founder mutations tested; square bullet (■) and ** = specific study population (but not A. *Jewish)*, *in which only founder mutations were tested; — = 95% Confidence interval (only for hazard ratios); CGC based studies with ext*. *ref*. = *CGC based studies with external reference group; CGC based studies with int*. *ref*. = *CGC based studies with internal reference group; Sign = statistically significant (P < 0*.*05); NS = not statistically significant; NR = not reported; †Adjusted for clinico-pathological characteristics and/or treatment*.

Although the forest plots of absolute survival differences in Fig. 4A and HRs in Fig. 4B showed a tendency towards worse unadjusted overall survival for *BRCA2* mutation carriers compared to ‘non-carriers’, the absolute survival differences were small, mostly below 10%, and the results were inconsistent, certainly among the HQ studies (S5 Supporting Information, part A). The pooled estimates showed only a small overall survival difference between *BRCA2* carriers and ‘non-carriers’: 2% 10-year worse survival and a pooled HR of 1.09 (95% CI 0.58–1.59); with a suggestion for statistical heterogeneity between the results (*P* = 0.07; Table 3 and S6 Supporting Information, panel G). Using the best-evidence synthesis, there was indecisive evidence for an association between *BRCA2* mutation carriership and unadjusted overall survival of breast cancer patients. Although the HQ studies reporting an adjusted HR (n = 3) found worse adjusted overall survival for *BRCA2* compared to ‘non-carriers’ (Fig. 4B), with our criteria there was insufficient evidence for a conclusion (Table 5).

#### *BRCA2* mutation carriership and breast cancer-specific survival

Based on the forest plots of absolute survival differences in Fig. 4C and HRs in Fig. 4D there seemed to be more studies reporting a worse breast cancer-specific survival for *BRCA2* compared to ‘non-carriers’ than studies reporting a better breast cancer-specific survival (S5 Supporting Information, part B). This worse survival was also supported by the pooled analyses, showing a 10-year absolute survival difference between the *BRCA2* and ‘non-carriers’ of about 15% (Table 3). The pooled, significant, unadjusted HR was 1.57 (95% CI 1.29–1.86) (Table 3 and S6 Supporting Information, panel H). This survival difference seemed to be driven by one large study [62], and, when using the best-evidence synthesis, the evidence was still judged to be indecisive. For adjusted breast cancer-specific survival too few HQ studies were available (Fig. 4D) and no conclusion could be drawn using the best-evidence synthesis (Table 5).

#### *BRCA2* mutation carriership and metastasis-free survival

There were only three studies [20,35] that determined the association between *BRCA2* mutation carriership and metastasis-free survival; the studies reported conflicting results (S5 Supporting Information, part C). Also, there were not enough HQ studies available to provide conclusive evidence using the best-evidence synthesis for an association between *BRCA2* mutation carriership and unadjusted/adjusted metastasis-free survival of breast cancer patients (Table 5).

#### *BRCA2* mutation carriership and recurrence-free survival

The five studies [17,20,28,58] which determined the association between *BRCA2* mutation carriership and recurrence-free survival reported inconsistent results (S5 Supporting Information, part D). Hence using the best-evidence synthesis there were not enough HQ studies available to provide conclusive evidence about the association between *BRCA2* mutation carriership and recurrence-free survival of breast cancer patients (Table 5).

#### *BRCA1* and *BRCA2* mutation carriership combined and survival

Though the focus of our review was to determine the association between breast cancer prognosis and carriership of the *BRCA1* and *BRCA2* mutations separately, there were many studies combining both groups in their analyses (S7 Supporting Information). Using the best-evidence synthesis for *BRCA1* and *BRCA2* mutation carriers combined (S7 Supporting Information, part E), for most of the unadjusted survival outcomes with sufficient HQ studies available, there was indecisive evidence because of the large heterogeneity of the results. Only for the association between *BRCA1/2* carriership and unadjusted overall survival there was nil evidence, implying no association. For all the adjusted outcomes less than four HQ studies were available and therefore no evidence could be provided.

### Sensitivity analysis

When using the best-evidence synthesis on all studies, irrespective of study quality, evidence remained indecisive for most outcomes or changed to nil (Table 4 and Table 5). When using only the unselected cohort studies (mostly HQ) for the best-evidence synthesis, for most outcomes evidence remained indecisive; however, there was moderate evidence for a worse unadjusted and adjusted overall survival for *BRCA1* mutation carriers compared to non-carriers (S8 Supporting Information). In the sensitivity analyses with all studies and the ‘unselected cohort studies’ the moderate evidence for a worse recurrence-free survival for *BRCA1* mutation carriers changed to nil and indecisive respectively.

S9 Supporting Information shows a summary of all other sensitivity analyses performed for the best-evidence synthesis. When the absolute survival and HR cut-offs in the best-evidence synthesis were less stringent (than the 10% absolute difference or HRs ≤0.88 or ≥1.14), the evidence for a worse survival for *BRCA1* and/or *BRCA2* compared to ‘non-carriers’ became stronger for most of the outcomes, i.e. from indecisive to moderate evidence, or remained the same. With more stringent cut-offs, the evidence became weaker for most of the outcomes, i.e. from indecisive to nil evidence, or remained the same. Only for the association between *BRCA1* carriership and unadjusted (worse) recurrence-free survival the moderate evidence held in all the sensitivity analyses. In the sensitivity analysis where only the statistically significant associations were considered, the evidence changed for most outcomes; mostly from indecisive to nil evidence.

### Effects of confounders/mediating factors on the association between BRCA1 and BRCA2 mutation carriership and prognosis

It is already known that breast cancers in carriers of *BRCA1* mutations exhibit different pathological characteristics compared to tumours in non-carriers, leading to treatment differences [2,3]. Also in the studies included in this review, there were many differences reported in tumour characteristics between *BRCA1* and also *BRCA2* mutation carriers compared to ‘non-carriers’ (S10 Supporting Information, part A). Only 32 studies reported HRs adjusted for tumour characteristics and/or treatment (Table 2).

To examine the effect of adjustment for confounders on the prognosis of *BRCA1* and *BRCA2* mutation carriers, we compared pairs of an unadjusted HR (HRunadjusted) and adjusted HR (HRadjusted). In general, the associations between *BRCA1/2* carriership and survival became less strong after adjustment for confounders, especially when the unadjusted results showed a worse survival for the carriers (Table 6; S10 Supporting Information, part B).

**Table 6:** Table of studies reporting an unadjusted and adjusted hazard ratio.

Mutation	Out-come	Authors + year	Study type	% of max QS	HR^a^	95% CI	HR^b^	95% CI	Unadjusted survival carriers compared to ‘non-carriers’?^c^	Direction of the difference adjusted vs. unadjusted survival	HR adjusted for:						
											Grade	Stage	*size*	*N*	*M*	ER	Treatment
BRCA1	OS	Verhoog (1998)	CGC based with ext. ref.	42	**1.04**	0.63–1.71	**1.21**	0.72–2.04	Worse	↑		x					
		Brekelmans (2007)	CGC based with ext. ref.	52	**1.01**	0.75–1.37	**1.3**	0.91–1.85	Worse	↑	x	x				x	x
		Lee (2011)	CGC based with int. ref.	47	**0.64**	0.27–1.37	**0.73**	NR	Better	=		x					
		Stoppa-Lyonnet (2000)	CGC based with int. ref.	52	**5.1**	NR	**3.5**	1.3–9.7	Worse	↓				*x*			
		Goodwin (2012)	CGC based with int. ref.	76	**1.43**	0.91–2.23	**0.99**	0.62–1.59	Worse	↔	x		*x*	*x*		x	
		Bonadona (2007)	Unselected cohort	69	**0.67**	0.16–2.77	**0.29**	0.04–2.26	Better	↑	x		*x*	*x*		x	
		Goode (2002)	Unselected cohort	72	**4.14**	1.32–13	**1.99**	0.47–8.45	Worse	↓	x						
		Huzarski (2013)	Unselected cohort	82	**1.13**	0.83–1.57	**1.81**	1.26–2.61	Worse	↑	x			*x*	*x*	x	x
		Goffin (2003)	Unselected cohort	82	**1.9**	0.99–3.6	**1.4**	0.7–2.9	Worse	↓	x		*x*	*x*			
		Rennert (2007)	Unselected cohort	90	**1.09**	0.79–1.51	**1.13**	0.78–1.66	Worse	=			*x*	*x*	*x*		
	BCSS	Brekelmans (2007)	CGC based with ext. ref.	52	**0.89**	0.63–1.25	**1.21**	0.83–1.76	Better	↔	x	x				x	x
		Lee (2011)	CGC based with int. ref.	47	**0.58**	0.25–1.25	**0.73**	NR	Better	↓		x					
		Bonadona (2007)	Unselected cohort	69	**0.67**	0.16–2.77	**0.29**	0.04–2.26	Better	↑	x		*x*	*x*		x	
		Rennert (2007)	Unselected cohort	90	**1.08**	0.72–1.63	**0.76**	0.45–1.3	Worse	↔				*x*	*x*		
		Chappuis (2005)	Unselected cohort	96	**1.9**	0.99–3.6	**0.8**	0.4–1.6	Worse	↔	x		*x*	*x*		x	
	RFS	Verhoog (1998)	CGC based with ext. ref.	42	**1**	0.65–1.55	**1.09**	0.7–1.7	Equal	=		x					
		Brekelmans (2007)	CGC based with ext. ref.	52	**0.92**	0.56–1.51	**0.84**	0.41–1.75	Better	=	x	x				x	x
	MFS	Brekelmans (2007)	CGC based with ext. ref.	52	**0.71**	0.52–0.96	**1.25**	0.87–1.92	Better	↔	x	x				x	x
		Lee (2011)	CGC based with int. ref.	47	**0.79**	0.38–1.58	**0.9**	NR	Better	↓		x					
		Stoppa-Lyonnet (2000)	CGC based with int. ref.	52	**3.5**	NR	**2.6**	1–6.5	Worse	↓				*x*		x	
		Goodwin (2012)	CGC based with int. ref.	76	**1.19**	0.74–1.89	**0.83**	0.51–1.35	Worse	↔	x		*x*	*x*		x	
		Bonadona (2007)	Unselected cohort	69	**0.47**	0.12–1.94	**0.24**	0.03–1.82	Better	↑	x		*x*	*x*		x	
		Goffin (2003)	Unselected cohort	82	**1.6**	0.9–2.9	**1.2**	0.7–2.4	Worse	↓	x		*x*	*x*			
BRCA2	OS	Verhoog (1999)	CGC based with ext. ref.	46	**0.75**	0.37–1.51	**0.59**	0.27–1.59	Better	↑		x					
		Goodwin (2012)	CGC based with int. ref.	76	**1.81**	1.15–2.86	**1.12**	0.7–1.79	Worse	↓	x		*x*	*x*		x	
		Budroni (2009)	Unselected cohort	48	**0.7**	0.46–1.36	**0.8**	0.48–1.62	Better	=		x					
		Rennert (2007)	Unselected cohort	90	**1.07**	0.73–1.58	**1.2**	0.77–1.86	Worse	↑			*x*	*x*	*x*		
	BCSS	Loman (2000)	CGC based with ext. ref.	36	**2**	1.2–3.4	**1.6**	0.85–3.1	Worse	↓		x					
		Heikkinen (2009)	CGC based with ext. ref.	42	**2.34**	1.5–3.66	**2.06**	1.03–4.15	Worse	↓	x		*x*	*x*	*x*		
		Tryggvadottir (2013)	Unselected cohort	76	**1.61**	1.32–1.96	**0.98**	0.64–1.48	Worse	↔	x			*x*	*x*	x	
		Rennert (2007)	Unselected cohort	90	**1.42**	0.92–2.19	**1.31**	0.8–2.15	Worse	↓			*x*	*x*	*x*		
	RFS	Verhoog (1999)	CGC based with ext. ref.	46	**0.92**	0.52–1.62	**0.84**	0.44–1.63	Better	=		x					
	MFS	Goodwin (2012)	CGC based with int. ref.	76	**1.63**	1.02–2.6	**1**	0.62–1.61	Worse	↓	x		*x*	*x*		x	

The results are sorted on the mutation and survival outcome studied, and on the quality score of the study.

*OS = overall survival*; *BCSS = breast cancer-specific survival; RFS = recurrence-free survival; MFS = metastasis-free survival; CGC based with ext*. *ref*. = *CGC based study with external reference group; CGC based with int*. *ref*. = *CGC based study with internal reference group; Unselected cohort = Unselected cohort study;*

^*a*^ *Unadjusted Hazard ratio;*

^*b*^ *Adjusted Hazard ratio;*

^*c*^ *Definition of a better survival = HR < 1*.*00; definition of a worse survival = HR > 1*.*00;*

= *no difference (difference < 0*.*1) between the effects;*

*↑ effect in the same direction but stronger (difference > 0*.*1);*

*↓ effect in the same direction but weaker (difference > 0*.*1);*

*↔ effects in the opposite direction*.

Only in four studies [11,20,47,63] adjuvant treatment was considered as a confounder in the analyses (Table 6) and in six studies [11,31,35,49,53,63] analyses were stratified on chemotherapy (data not shown). In most studies a tendency towards a worse survival for *BRCA1* mutation carriers compared to ‘non-carriers’ was shown in the subgroup of patients not treated with adjuvant chemotherapy, and no difference in survival in those treated with chemotherapy. One study by Rennert and colleagues [49] reported a significant interaction between *BRCA1* status and chemotherapy (*P =* 0.02). Goodwin and colleagues [35] showed a worse outcome for *BRCA2* carriers compared to ‘non-carriers’ not treated with chemotherapy (HR 3.6, 95% CI 1.5–9.0). Only two studies [20,63] took prophylactic surgery into account as an (time-varying) confounder in the analyses.

### Exploring heterogeneity between the studies

Based on the forest plots of all above results (Fig. 3 and Fig. 4), there were indications for substantial heterogeneity between the studies. Using graphic analysis we determined the influence of the different types of bias on the heterogeneity (S11 Supporting Information) using the 5-year absolute difference and the adjusted HR for overall survival for *BRCA1* mutation carriers compared to ‘non-carriers’ since for these data most studies were available.

Studies with less misclassification bias appeared to more often report a worse survival for *BRCA1* mutation carriers compared to ‘non-carriers’, with stronger effects (S11 Supporting Information, panel C). This might be explained by a larger contrast between carriers and the ‘non-carrier group’ when all non-carriers are tested, a feature incorporated in the misclassification score. Within the item of selection bias the proportion of incident cases, but not study type, seemed to reduce the heterogeneity of the results (S11 Supporting Information, panels A and B). Unfortunately, duration and completeness of follow-up time were often not reported, so we could not assess the effect of these variables effect on the heterogeneity of the results. To see whether the extent of confounding in the studies explained the heterogeneity of the adjusted risk estimates, we graphically compared the adjusted HR to the percentage score of ‘confounding/accounting for mediating variables’ bias in the studies. From this graph a clear relation between the heterogeneity of the results and percentage of confounding was apparent, though due to the small number of studies it was difficult to draw firm conclusions (S11 Supporting Information, panel D).

### Exploring publication bias

S12 Supporting Information shows the funnel plot for studies reporting the 5-year overall survival for *BRCA1* mutation carriers compared to ‘non-carriers’. The funnel plot showed no clear evidence of publication bias.

## Discussion

Our review shows that, in contrast to currently held beliefs of many oncologists and despite 66 published studies, it is not yet possible to draw evidence-based conclusions about the association between *BRCA1* and/or *BRCA2* mutation carriership and breast cancer prognosis. We only found sufficient evidence for a 10% worse unadjusted recurrence-free survival for *BRCA1* mutation carriers. For all the other outcomes the evidence was judged to be indecisive. Although two less extensive reviews about *BRCA1* and *BRCA2* carriership and breast cancer-specific survival have been published [64,65], this review is the first to use a systematic approach and standardized analysis, taking into account the methodological rigor of all the available studies, to arrive at the best evidence.

Despite the lack of evidence for a worse survival for *BRCA1* and *BRCA2* mutation carriers, we do see a tendency towards a survival disadvantage for all outcomes. E.g., although the best-evidence synthesis judged the evidence indecisive due to inconsistent findings and small effects, the pooled estimate shows a worse 10-year absolute breast cancer-specific survival difference of 14.8% for *BRCA2* carriers (Table 3, Fig. 3 and Fig. 4). Unfortunately, the large variation in the types of outcomes and the conflicting results reported between studies reduced the power for evidence-based conclusions for most of the outcomes. The most reported outcome was overall survival. However, we considered overall survival as the least relevant outcome because this is also affected by the increased ovarian cancer mortality in carriers; an issue that was rarely mentioned in the reviewed papers. The only outcome for which we found evidence that there was an association with *BRCA1* mutation carriership, i.e., unadjusted recurrence-free survival, is a heterogeneous survival measure with inconsistent definitions (often not even reported) across studies.

Considering that certain prognostically important clinico-pathological features are different for *BRCA1*-associated tumours (S10 Supporting Information, part A) [2,3], a crucial question is to which extent *BRCA1/2* mutation carriership and the specific tumour features associated with carriership can be considered to be independent when studying prognosis. The heterogeneity of the reported results did not allow a conclusion regarding the contribution of *BRCA1/2* status and tumour features to a worse survival (Fig. 3 and Fig. 4; Table 4 and Table 5). However, individual and pooled adjusted HRs compared to unadjusted HRs often resulted in a shift to a relatively more favourable survival for both *BRCA1* and *BRCA2* mutation carriers compared to ‘non-carriers’ (Table 3 and Table 6). Based on these results we can conclude that clinico-pathological characteristics of the tumour might indeed play a confounding or mediating role in the association between *BRCA1/2* mutation carriership and breast cancer survival, though more research should be performed to further elucidate this.

Primary breast cancer treatments may be different for *BRCA1* and *BRCA2* mutation carriers compared to non-carriers, mostly related to different pathological features of tumours in carriers (S10 Supporting Information, part A) [2,3]. Although the data are scarce, our review supports what was earlier suggested by others [66], i.e. that that the therapy response of tumours in *BRCA1/2* mutation carriers might be better compared to that in non-carriers. Future studies should provide insight into the potential confounding or mediating role of treatment when examining survival of *BRCA1/2* mutation carriers.

To explain the large heterogeneity between the results reported in the included studies, we examined whether this was related to the extent of selection bias (largely dependent on whether incident cases were included and the type of comparison group used), the extent of misclassification bias (largely dependent on whether non-carriers were tested) and the amount of confounding bias in the different studies. Surprisingly, the only two factors that seem to explain part of the heterogeneity were misclassification bias; when a study had not tested the comparison (‘non-carriers’) group, and the proportion of incident cases (S11 Supporting Information, panels C and D). The sensitivity analysis of the best-evidence synthesis including only ‘unselected cohort studies’ indeed showed that the results altered when including only these type of studies (S8 Supporting Information). Furthermore, the other sensitivity analyses of the best-evidence synthesis (S9 Supporting Information) highlighted that the potential associations we are reviewing in this paper appear to be very weak (absolute differences around 5%). Moreover, it showed the lack of power in the individual studies; the already limited evidence from the best-evidence synthesis disappeared in the sensitivity analysis which only considered statistically significant results. Other reasons for the large heterogeneity and generally weak associations observed might be population differences (i.e. different mutations), differences in completeness of follow-up (often not reported), differences in consideration of contralateral breast cancer and prophylactic surgeries (usually not reported). Publication bias is unlikely to play a large role, as shown in our funnel plot; because of the low prevalence of *BRCA1/2* mutations in populations, also studies with only a small number of carriers were published.

The evidence-based conclusions drawn in our review are based on a tool, the best-evidence synthesis, which makes it possible to perform a standardized analysis of the available literature (tool developed by Monninkhof and colleagues [6], adapted by the authors for this review). The cut-offs for a relevant survival difference were arbitrarily chosen, but were defined a priori and were based on previous knowledge regarding breast cancer survival. In addition, the quality scores given to specific study aspects were developed with an expert group. The best-evidence synthesis only used the HQ studies (at least 50% of attainable quality score awarded); when performing the best-evidence synthesis using all studies (Table 4 and Table 5) the results substantially changed, which indicates that HQ studies are indeed different from the other studies. This confirmed our idea that we took into account the most important sources of bias. Even so, it should be kept in mind that our scoring system is not a direct measure of validity and may not capture all methodological aspects adequately.

Two earlier published reviews also addressed the association between *BRCA1/2* carriership and breast cancer survival. Bordeleau and colleagues [64] included 25 studies and described the methodological problems of the studies per calendar period of publication. According to this review, the data provided reassurance that the overall prognosis of *BRCA*-associated breast cancer was similar to that of breast cancer not associated with *BRCA* mutations. For studies published in the 1990s they found several methodological limitations leading to inconclusive results. For more recently published studies they reported improved methodology but failure to demonstrate a significant overall survival difference. In our review we did not find a relation between the publication year and the quality of the studies (data not shown). The other review, published in 2010 by Lee and colleagues [65], included 17 studies and described methodological problems of these studies in the discussion section. They performed a meta-analysis on short-term (5-year) and long-term (10-year) overall and progression-free survival and based their final conclusions on the pooled estimate, although they stated that there was inconsistency in the results. Overall they concluded that *BRCA1* mutation carriership appears to decrease both short-term and long-term overall survival rates and short-term but not long-term progression-free survival. For *BRCA2* mutation carriers they observed no effect on either short-term or long-term survival. While these two reviews reached conflicting conclusions, they also differ from conclusions in our review, probably due to our more complete inclusion of papers and systematic way of analysing the results, as well as evaluation of the methodological aspects and the quality of the included studies.

On the basis of our systematic and evidence-based analysis of all studies published to date, we conclude that there is only moderate evidence for a worse recurrence-free survival for *BRCA1* mutation carriers, unadjusted for tumour characteristics. For all the other outcomes the evidence was judged to be indecisive, though if analysed in isolation, the ‘unselected cohort studies’ showed moderate evidence for a worse overall survival for *BRCA1* mutation carriers. Survival perspectives of *BRCA1/2* mutation carriers diagnosed with breast cancer are unclear and current evidence does not support differential treatment decisions (apart from the use of PARP inhibitors).

More high quality studies are needed that include a large number of incident breast cancer cases who are unselectively tested for *BRCA* mutations, with sufficient follow-up time, and information available on all patient and tumour characteristics, treatment and prophylactic surgeries. Our quality scoring system can help researchers when considering specific aspects of design and analysis which are important to reduce bias.

## Supporting Information

S1 Supporting Information Quality scoring system—observational studies of the association between *BRCA1/2* carriership and breast cancer survival. (PDF)

S2 Supporting Information Best-evidence synthesis: classification of the level of evidence of a worse breast cancer survival for *BRCA1/2* mutation carriers compared to ‘non-carriers’. (PDF)

S3 Supporting Information Numbers of studies reporting a specific risk estimate (per mutation type and outcome). (PDF)

S4 Supporting Information Results *BRCA1* mutation carriership. (PDF)

S5 Supporting Information Results *BRCA2* mutation carriership. (PDF)

S6 Supporting Information Forest plots of high quality (HQ) studies, based on the Random effect (DerSimonian and Laird) analyses. (PDF)

S7 Supporting Information Results *BRCA1* and *BRCA2* mutation carriership combined. (PDF)

S8 Supporting Information Sensitivity analysis, using only the ‘Unselected cohort studies’, of the best-evidence synthesis for BRCA1 (panel A) and BRCA2 (panel B) mutation carriership and breast cancer prognosis. (PDF)

S9 Supporting Information Summary of the sensitivity analysis of the best-evidence synthesis for *BRCA1* (panel A), *BRCA2* (panel B) and *BRCA1/2* (panel C) mutation carriership and breast cancer prognosis. (PDF)

S10 Supporting Information Confounding and/or mediating factors. (PDF)

S11 Supporting Information Figures showing the association between of the percentage of selection bias (panels A and B), misclassification bias (panel C) confounding/accounting for mediating variables (panel D) present in the study and the heterogeneity of results. (PDF)

S12 Supporting Information Funnel plot showing the number of *BRCA1* mutation carriers included in the study related to the results defined as the 5-year overall survival difference for *BRCA1* mutation carriers compared to ‘non-carriers’. (PDF)

S13 Supporting Information Prisma Checklist. (PDF)
