# Supplementary material for: Worse Breast Cancer Prognosis of BRCA1/BRCA2 Mutation Carriers: What's the Evidence? A Systematic Review with Meta-Analysis
Source: PLoS One. 2015 Mar 27;10(3):e0120189. doi: 10.1371/journal.pone.0120189 (PMC4376645; doi:10.1371/journal.pone.0120189)
Supplement: S1 Supporting Information — (PDF) [file pone.0120189.s001.pdf]

S1 Supporting Information. Quality scoring system - observational studies of the association between *BRCA1/2* carriership and breast cancer survival.

A. Quality scoring system

Criteria		Score	Comments
**Selection bias**			
1. Percentage incident cases?	> 90% incident cases	129.0	*Some studies include prevalent cases and/or fatal cases without information about the date of diagnosis.*
	10%-90% incident cases	64.5	
	< 10% incident cases	0.0	
2. Was the entry into the study dependent on the outcome of *BRCA1/2* mutation testing (in the clinical genetic setting)?	No, unselected cohort study	87.0	*Important when testing occurred before the inclusion in the study (testing bias); i.e. tested patients more health conscious.*
	Yes, CGC based study with internal reference group	43.5	
	Yes, CGC based study with external reference group	0.0	
If question 2 was answered with " No, unselected cohort study" *or* "Yes, CGC based study with internal reference group" *, both questions 3 and 4 can be answered "yes" automatically and there can be preceded with question 5. If question 2 was answered "Yes, CGC based study with external reference group", questions 3 and 4 should be assessed.*			
3. Did the exposed (*BRCA1/2* mutation carriers) and unexposed (reference group) originate from the same source population?	Yes	22.5	
	No or not reported	0.0	
4. Were the same inclusion / exclusion criteria applied for exposed (*BRCA1/2* mutation carriers) and unexposed (reference group)?	Yes	21.0	
	No or not reported	0.0	
5. Percentage complete follow-up?	> 90% complete	84.0	
	60-90% complete	42.0	
	< 60% complete or not reported	0.0	
	**Maximal Selection bias score:**	**300**	
**Misclassification bias**			
6. Which outcome was used? (also, is it clearly defined?)	Breast cancer specific survival (+ overall survival)	21.0	*Disease specific survival includes BC specific survival and/or recurrence free survival.*
	Overall survival only	0.0	
7. Was contralateral breast cancer included in the outcome measure of recurrence?	Not included or not applicable	26.0	
	Included	0.0	
8. Was the follow-up information gathered similarly between exposed (*BRCA1/2* mutation carriers) and unexposed (reference group)?	Collected similarly (same source)	21.0	
	Not collected similarly or not reported	0.0	
9. Were the outcomes collected blinded?	Yes	16.0	*Important when the BRCA1/2 carriership status was known for the researcher who gathered the follow-up information.*
	No or not reported	0.0	
10. Was the comparison group also tested for *BRCA1/2* mutation carriership?	Yes	16.0	*There may be carriers in the comparison group which may give conservative results.*
	No	0.0	
	**Maximal Misclassification bias score:**	**100**	
**Confounding and/or mediating variables**			
11. Was family history taken into account in the analyses?	Yes	24.0	*Taken into account: reported and described what was done (e.g. corrected for in the analysis / distribution shown).*
	No	0.0	
12. Was the treatment of the breast cancer taken into account in the analysis?	Yes	56.0	*Taken into account: reported and described what was done (e.g. corrected for in the analysis / distribution shown).*
	No	0.0	
13. Was the stage (TNM) of the tumour taken into account in the analysis?	Yes	36.0	*Taken into account: reported and described what was done (e.g. corrected for in the analysis / distribution shown).*
	No	0.0	
14. Was the grade and ER status of the tumour taken into account in the analysis?	Yes	38.0	*Taken into account: reported and described what was done (e.g. corrected for in the analysis / distribution shown).*
	No	0.0	
15. Is the survival analysis performed correctly?	Yes	46.0	*(e.g. not only an actuarial curve but also a multivariate Cox / Poisson model at least adjusted for age at diagnosis)*
	Mediating	23.0	
	No or not reported	0.0	
	**Maximal Confounding and/or mediating variables score:**	**200**	
	**Maximal total score:**	**600**	

B. Explanation Quality scoring system

The quality scoring system was developed to classify observational studies determining the prognosis of breast cancer patients carrying a *BRCA1* or *BRCA2* mutation into different categories based on their study quality and to explore whether this contributes to the discrepancies in outcome among the studies. The scoring system includes general methodological aspects as well as specific aspects of these studies and can be applied to all types of observational studies used to study this topic: studies selecting the *BRCA1/2* mutation carriers in a clinical setting, and studies selecting carriers from population-based cohorts.

The potential forms of bias were categorized according to three main groups of errors in these studies: selection bias, misclassification bias and confounding /accounting for mediating variables, each represented by five questions to capture this form of bias. For a correct quantitative distribution of the score, six experts (epidemiologists/methodologists, of whom five specialists in this field of research and one more general methodologist) were asked to assign weights to the individual questions; the median weight of the panel was used for the calculation of the final score for each question. In addition, the three groups of bias were also weighted relatively to each other (3:1:2). In this research field, the potential for misclassification bias is thought to be less important than the other forms of bias. Inaccuracy in measurement and classification is small, because of the precise tests for *BRCA1/2* mutations and robust outcomes studied (survival). On the other hand, selection bias is seen as most important; there are major systematic differences between the selections of the carriers (from the clinical genetic setting or unselected) and the non-carrier comparison groups in this type of studies. Also confounding/accounting for mediating variables are important to take into account in the quality assessment because of proposed confounding/mediating role of these factors in the relation between *BRCA1/2* mutation carriership and breast cancer survival (*BRCA1*-associated breast tumours are known to exhibit different clinico-pathological features compared to sporadic tumours (Supporting information S10, panel A) [1,2].
For every paper a minimum score of zero and a maximum score of six hundred could be assigned; selection bias contributing maximal 300 points, misclassification bias 100 points and confounding/accounting for mediating variables 200 points, to represent the relative weights of 3:1:2.

References

1. Phillips KA. Current perspectives on BRCA1- and BRCA2-associated breast cancers. Intern Med J. 2001;31: 349-356.
2. Honrado E, Osorio A, Palacios J, Benitez J. Pathology and gene expression of hereditary breast tumors associated with BRCA1, BRCA2 and CHEK2 gene mutations. Oncogene. 2006;25: 5837-5845.
