# Supplementary material for: Worse Breast Cancer Prognosis of BRCA1/BRCA2 Mutation Carriers: What's the Evidence? A Systematic Review with Meta-Analysis
Source: PLoS One. 2015 Mar 27;10(3):e0120189. doi: 10.1371/journal.pone.0120189 (PMC4376645; doi:10.1371/journal.pone.0120189)
Supplement: S2 Supporting Information — (PDF) [file pone.0120189.s002.pdf]

## S2 Supporting Information. Best-evidence synthesis: classification of the level of evidence of a worse breast cancer survival for *BRCA1/2* mutation carriers compared to 'non-carriers'.

| **Level of evidence** for a **worse** survival for BRCA carriers compared to 'non-carriers' | **Study results** | | |
|---|---|---|---|
| | % HQ studies reporting a **worse survival**[b] | % HQ studies reporting a **better survival**[c] | % HQ studies reporting **no association**[d] |
| **Strong evidence**[a] | > 75 % | . | |
| **Moderate evidence**[a] | 60 - 75 % | < 25 % | . |
| | 50 - 60 % | < 10 % | . |
| **Nil**[a] | . | > 60 % | |
| | . | > 40 % | . |
| **Indecisive evidence** | all other options / <4 HQ studies available | | |

[a]*At least four studies are necessary to define the evidence as strong, moderate or nil.*
[b]*A better survival for carriers compared to 'non-carriers' was defined as absolute survival differences of larger than -10% or risk estimates below 0.88 independent of statistical significance.*
[c]*A worse survival for carriers compared to non- carriers was defined as absolute survival differences of larger than 10% or risk estimates higher than 1.14 independent of statistical significance.*
[d]*No association was considered as absolute survival differences between -10 and 10% and risk estimates between 0.88 and 1.14 independent of statistical power.*
