# Supplementary material for: Worse Breast Cancer Prognosis of BRCA1/BRCA2 Mutation Carriers: What's the Evidence? A Systematic Review with Meta-Analysis
Source: PLoS One. 2015 Mar 27;10(3):e0120189. doi: 10.1371/journal.pone.0120189 (PMC4376645; doi:10.1371/journal.pone.0120189)
Supplement: S3 Supporting Information — (PDF) [file pone.0120189.s003.pdf]

S3 Supporting Information. Numbers of studies reporting a specific risk estimate (per mutation type and outcome).

		All studies					HQ studies[b]					
		Total[b]	Risk estimate				Total[c]		Risk estimate			
			5yr	10yr	HR	HR[a]	Unadjusted[d]	Adjusted[e]	5yr[d]	10yr[d]	HR[d]	HR[ae]
BRCA1	OS	37	31	24	13	14	17	22	15	12	6	11
	BCSS	14	10	9	6	6	7	10	4	6	3	5
	MFS	9	7	4	6	7	5	8	3	2	3	6
	RFS	17	15	8	2	2	7	11	6	3	0	1
BRCA2	OS	18	16	11	5	6	10	9	9	7	3	3
	BCSS	10	8	8	4	5	4	6	2	4	2	3
	MFS	4	3	3	1	2	2	4	1	1	1	2
	RFS	6	5	2	1	2	1	3	1	0	0	1
BRCA1/2	OS	20	17	9	5	3	10	11	7	3	3	2
	BCSS	5	3	2	3	2	4	5	2	2	2	2
	MFS	4	4	1	2	2	4	4	4	1	2	2
	RFS	17	17	10	5	4	5	8	5	2	1	3

When more than 4 HQ studies were available, this is indicated in grey. 5/10 yr = absolute 5/10 year survival;HR = hazard ratio; HR[a] = adjusted hazard ratio; OS = Overall survival; BCSS = Breast cancer-specific survival; MFS = Metastasis-free survival; RFS = Recurrence-free survival. [b]Studies achieving at least 50% of the maximum quality score; [c]Total number of studies with any risk estimate reported; [d]The scores for confounding/accounting for mediating variables were not taken into account when determining whether a study was high quality; [e]The scores for confounding/accounting for mediating variables were taken into account when determining whether a study was high quality.
