# Supplementary material for: Worse Breast Cancer Prognosis of BRCA1/BRCA2 Mutation Carriers: What's the Evidence? A Systematic Review with Meta-Analysis
Source: PLoS One. 2015 Mar 27;10(3):e0120189. doi: 10.1371/journal.pone.0120189 (PMC4376645; doi:10.1371/journal.pone.0120189)
Supplement: S4 Supporting Information — (PDF) [file pone.0120189.s004.pdf]

S4 Supporting Information. Results *BRCA1* mutation carriership.

Forest plots (the forest plots are also shown outside the Supporting information (Figs. 3A-G), but are repeated here for readability): size of the bullet represents the number of included carriers; black bullet = HQ study; round bullet (●) and * = A. Jewish study population, only founder mutations tested; square bullet (■) and ** = specific study population (but not A. Jewish), in which only founder mutations were tested; — = 95% Confidence interval (only for hazard ratios); CGC based studies with ext. ref. = CGC based studies with external reference group; CGC based studies with int. ref. = CGC based studies with internal reference group; Sign = statistically significant ($P < 0.05$); NS = not statistically significant; NR = not reported; †Adjusted for clinico-pathological characteristics and/or treatment.

A. *BRCA1* mutation carriership and overall survival (OS)

Absolute OS differences: *BRCA1* mutation carriers compared to 'non-carriers'

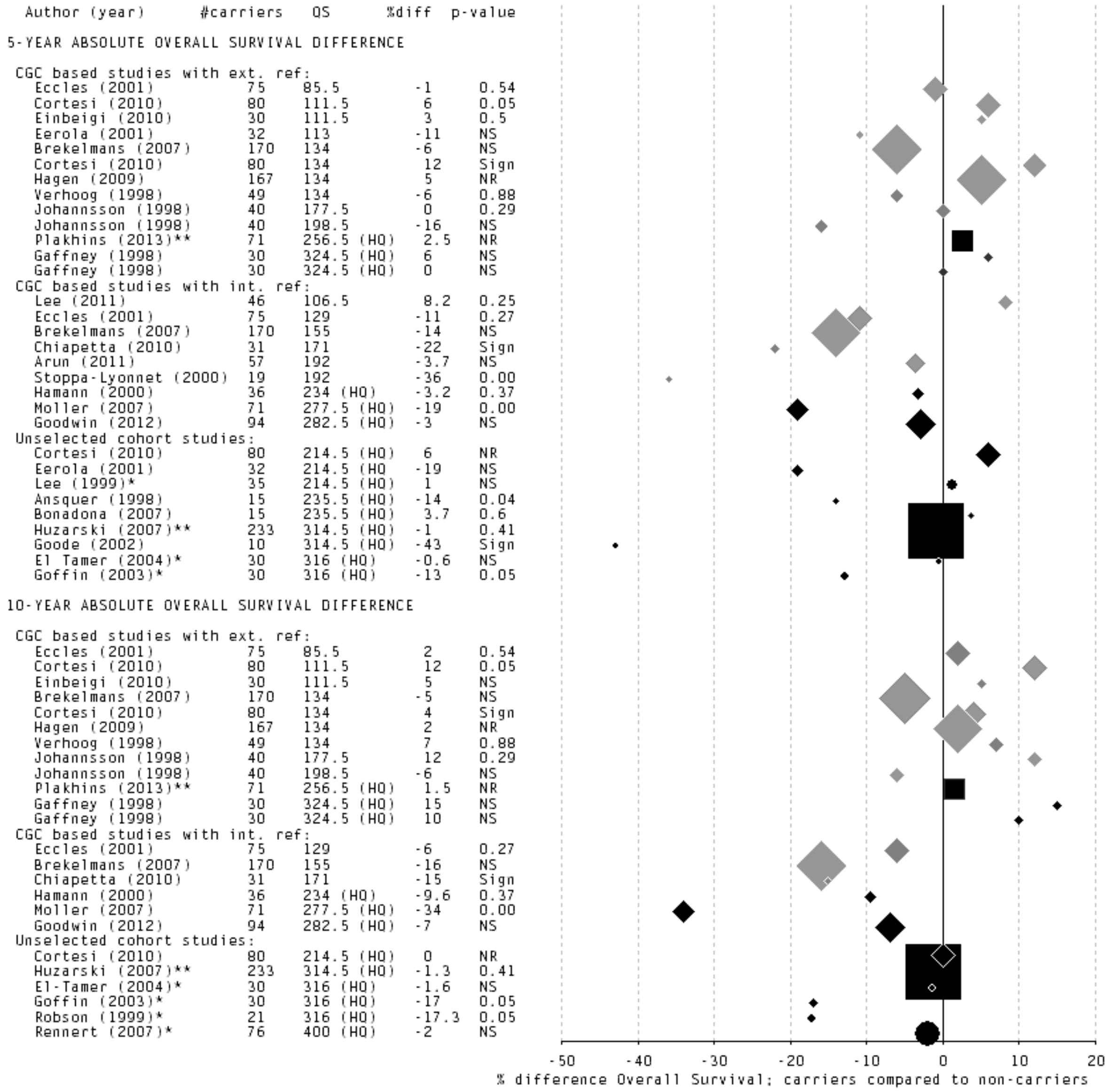

The forest plot above shows the absolute OS differences of *BRCA1* compared to 'non-carriers' reported by studies included in this review. Ten studies [1-8] (32%) reported a 1% to 12% better 5-year absolute OS for *BRCA1* mutation carriers; only two studies [6] reported a statistically significant difference. In contrast, 19 studies [9-24] (61%) reported a worse 5-year absolute OS for carriers, with differences ranging from 0.6% to 43%, including six [11,13-15,18,19] significant results. For 10-year absolute OS, ten studies [1,2,4,6,7,9,16,21] (42%) reported a 1.5% to 15% better OS for *BRCA1* mutation carriers, of which two substudies [6] reported a significant association. Thirteen studies [9,10,12,15-20,24-26] (54%) reported a worse 10-year OS for *BRCA1* mutation carriers, ranging from 1.3% to 34%, with four [15,18,19,25] significant results.

Hazard ratios for OS: *BRCA1* mutation carriers compared to 'non-carriers'

Author (year)	#carriers	OS	HR	CI95%
UNADJUSTED HAZARD RATIO FOR OVERALL SURVIVAL				
CGC based studies with ext. ref:				
Brekelmans (2007)	170	134	1.01	0.75-1.37
Verhoog (1998)	49	134	1.04	0.63-1.71
Jóhannsson (1998)	40	177.5	1.5	0.9-2.4
Jóhannsson (1998)	40	198.5	1.5	0.6-3.7
CGC based studies with int. ref:				
Lee (2011)	46	106.5	0.58	0.25-1.25
Stoppa-Lyonnet (2000)	19	192	5.1	Sign
Goodwin (2012)	94	282.5 (HQ)	1.43	0.91-2.23
Unselected cohort studies:				
Vinodkumar (2007)	11	193.5	3.7	Sign
Bonadona (2007)	15	235.5 (HQ)	0.67	0.16-2.77
Goode (2002)	10	314.5 (HQ)	4.14	1.32-13
Huzarski (2013)**	233	314.5 (HQ)	1.13	0.83-1.57
Goffin (2003)*	30	316 (HQ)	1.9	0.99-3.6
Rennert (2007)*	76	400 (HQ)	1.09	0.79-1.51
ADJUSTED† HAZARD RATIO FOR OVERALL SURVIVAL				
CGC based studies with ext. ref:				
Eerola (2001)	32	172	1.3	0.6-2.7
Verhoog (1998)	49	254	1.21	0.72-2.04
Brekelmans (2007)	170	310 (HQ)	1.3	0.91-1.85
Cortesi (2010)	80	310 (HQ)	0.29	0.13-0.62
Seynaeve (2004)	21	352 (HQ)	1.76	0.72-4.3
CGC based studies with int. ref:				
Lee (2011)	46	282.5	0.73	NS
Stoppa-Lyonnet (2000)	19	312 (HQ)	3.5	1.3-9.7
Plakhins (2011)**	93	315 (HQ)	1.1	0.81-1.48
Goodwin (2012)	94	282.5 (HQ)	1.43	0.91-2.23
Unselected cohort studies:				
Bonadona (2007)	15	411.5 (HQ)	0.29	0.04-2.26
Goode (2002)	10	434.5 (HQ)	1.99	0.47-8.45
Huzarski (2013)**	233	490.5 (HQ)	1.81	1.26-2.61
Goffin (2003)*	30	492 (HQ)	1.4	0.7-2.9
Rennert (2007)*	76	538 (HQ)	1.13	0.78-1.66

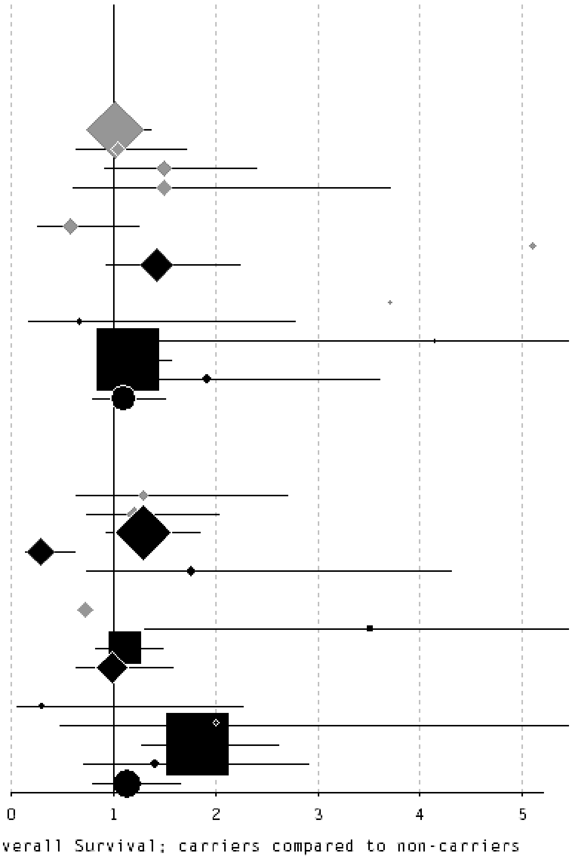

The forest plot above shows the univariate and multivariate hazard ratios for OS of *BRCA1* compared to 'non-carriers' reported by studies included in this review. Eleven [9,10,13-15,20,21,24,26,27] (85%) of the 13 studies [5,8-10,13-15,20,21,24,26,27] reporting an unadjusted hazard ratio (HR) observed a worse OS for *BRCA1* mutation carriers (HR>1), of which four [13-15,27] with statistically significant results. Also for the adjusted HRs for OS of *BRCA1* mutation carriers compared to 'non-carriers', most of the studies (ten [10,13-15,20-22,26,28,29] (71%) out of 14 studies [5,6,8,10,13-15,20-22,24,26,28,29]) reported a HR above 1, but only two results [10,13] reached significance.

B. BRCA1 mutation carriership and breast cancer-specific survival (BCSS)

Absolute BCSS differences: *BRCA1* mutation carriers compared to 'non-carriers'

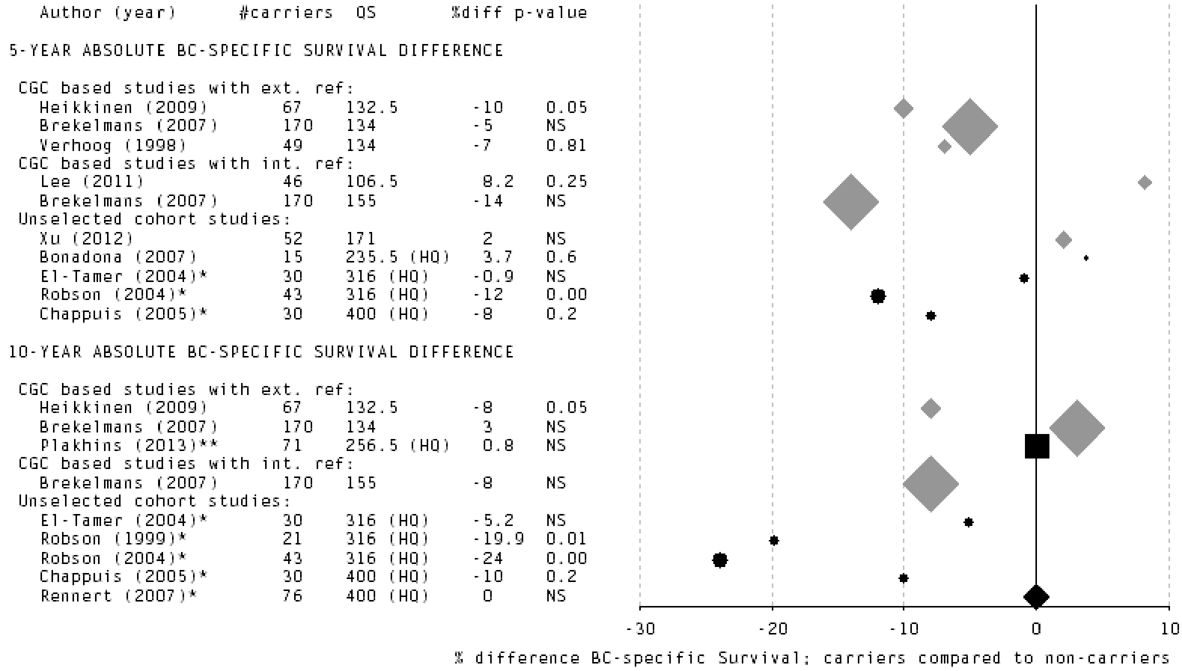

The forest plot above shows the absolute BCSS differences of *BRCA1* compared to 'non-carriers' reported by studies included in this review. Three studies [5,8,30] (30%) reported a non-significant 2% to 8.2% better 5-year absolute BCSS for *BRCA1* compared to non-carriers. On the other hand, seven studies [17,20,21,31-33] (70%) reported a worse 5-year absolute BCSS for carriers, ranging from 0.9% to 14%, including two [31,32] statistically significant results. Comparable results were found for 10-year absolute BCSS; two studies [2,20] (22%) reported a 0.8 and 3% better BCSS for *BRCA1* compared to 'non-carriers', while six other (sub)studies [17,20,25,31-33] (67%) reported a worse BCSS, with differences ranging from 5.2% to 24%, including three [25,31,32] statistically significant results.

Hazard ratios for BCSS: *BRCA1* mutation carriers compared to 'non-carriers'

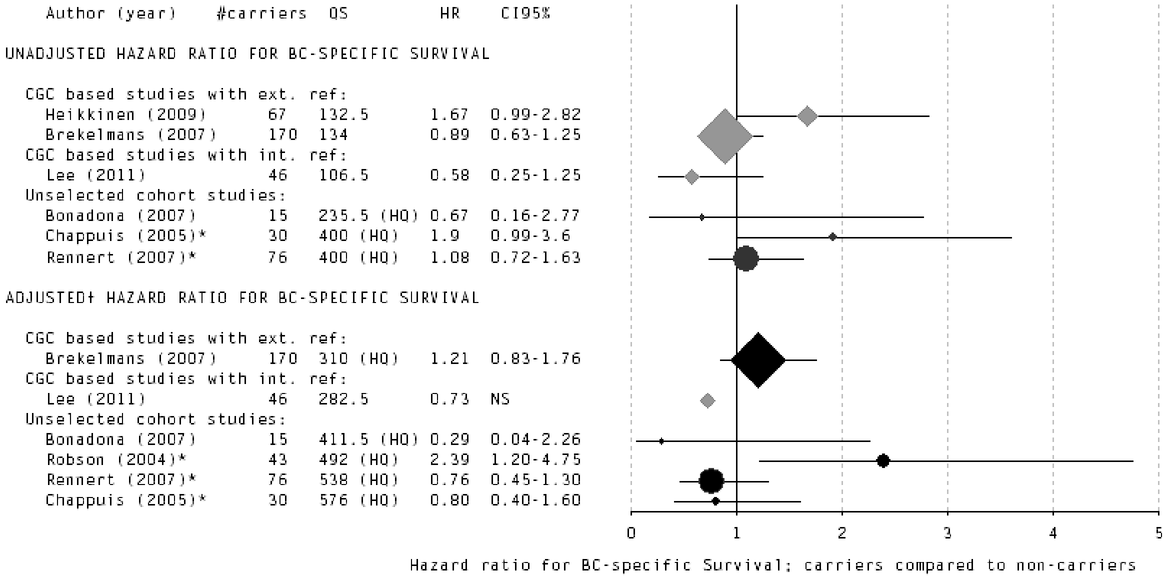

The forest plot above shows the univariate and multivariate hazard ratios for BCSS of *BRCA1* compared to 'non-carriers' reported by studies included in this review. Seven studies [5,8,20,26,31-33] reported an unadjusted and/or adjusted HR for BCSS. Three studies [5,8,20] (50%) reported a better and three studies [26,32,33] (50%) reported a worse unadjusted BCSS for *BRCA1* mutation carriers compared to 'non-carriers'. On the other hand, more of the studies reporting an adjusted HR observed a better adjusted BCSS for *BRCA1* mutation carriers (four

[5,8,26,33] (67%) out of six [5,8,20,26,31,33]); the one study [31] that found a statistically significant result showed a worse BCSS.

C. *BRCA1* mutation carriership and metastasis-free survival (MFS)

Absolute MFS differences: *BRCA1* mutation carriers compared to 'non-carriers'

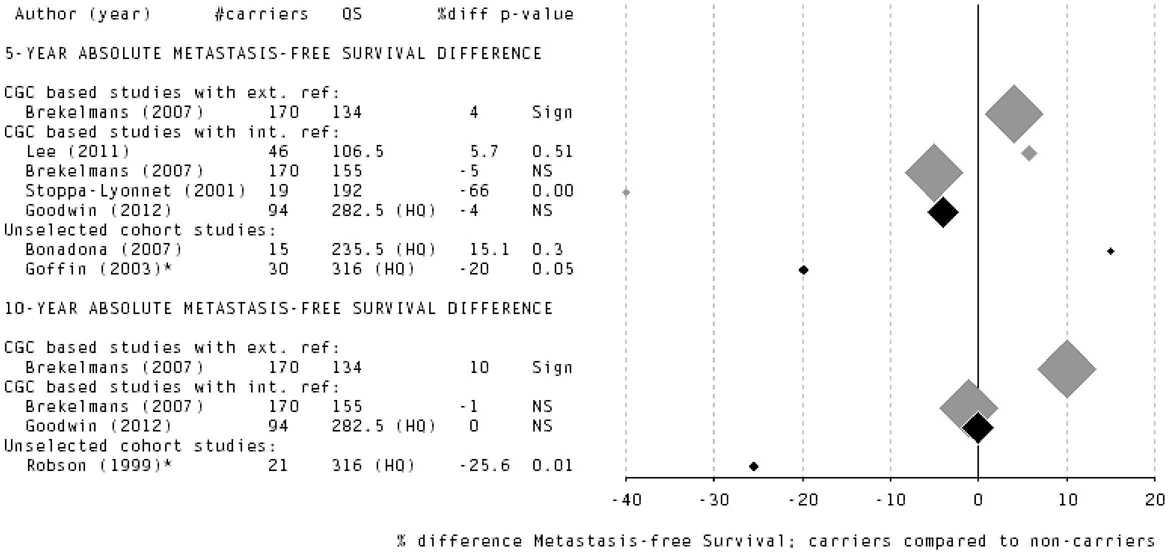

The forest plot above shows the absolute MFS differences of *BRCA1* compared to 'non-carriers' reported by studies included in this review. Three studies [5,8,20] (43%) reported a 4 to 15.1% better 5-year absolute MFS for *BRCA1* compared to 'non-carriers'. Four studies [13,15,20,24] (57%) reported a worse 5-year absolute MFS for *BRCA1* mutation carriers, of which two [13,15] were statistically significant with differences of 20% and 66%. Only four studies [20,24,25] reported the 10-year MFS for *BRCA1* mutation carriers compared to 'non-carriers'; two (sub)studies [20,25] (50%) reported a worse MFS, though one substudy [20] observed a difference of 1% only. The other (sub)studies [20,24] reported no difference in MFS or a 10% better MFS.

Hazard ratios for MFS: *BRCA1* mutation carriers compared to 'non-carriers'

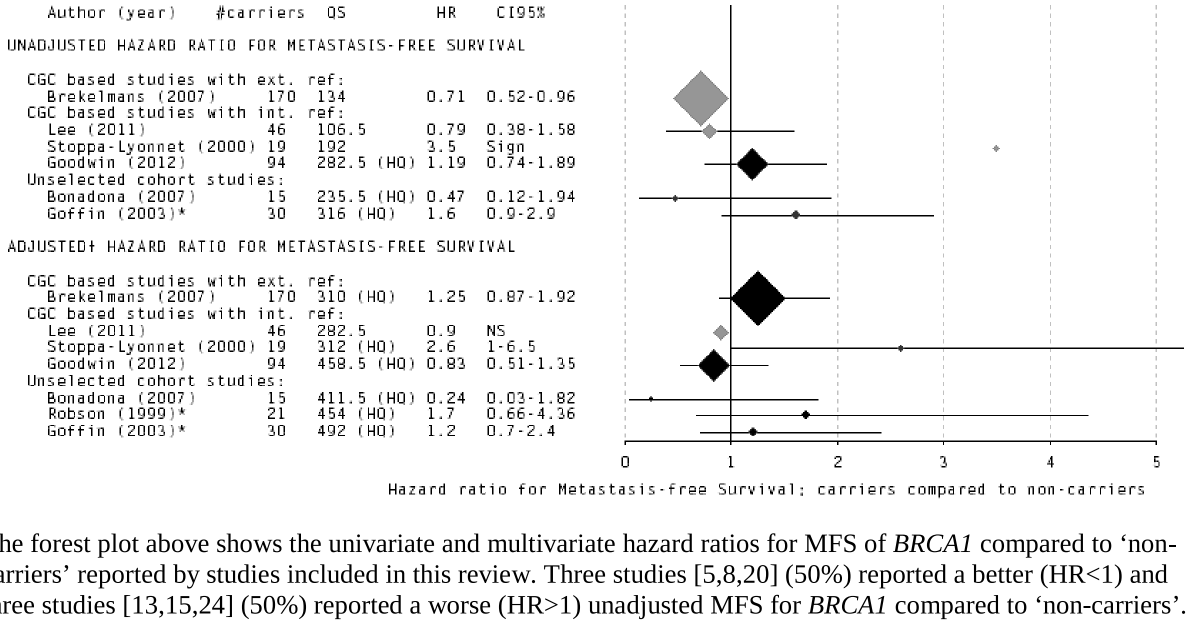

The forest plot above shows the univariate and multivariate hazard ratios for MFS of *BRCA1* compared to 'non-carriers' reported by studies included in this review. Three studies [5,8,20] (50%) reported a better (HR<1) and three studies [13,15,24] (50%) reported a worse (HR>1) unadjusted MFS for *BRCA1* compared to 'non-carriers'. All studies reporting an unadjusted HR, also reported an adjusted HR. Taking all the adjusted results together; there were four studies [13,15,20,25] (57%) reporting a worse adjusted MFS for *BRCA1* compared to 'non-carriers' and three studies [5,8,24] (43%) reporting a better adjusted MFS.

D. *BRCA1* mutation carriership and recurrence-free survival (RFS)

Absolute RFS differences: *BRCA1* mutation carriers compared to 'non-carriers'

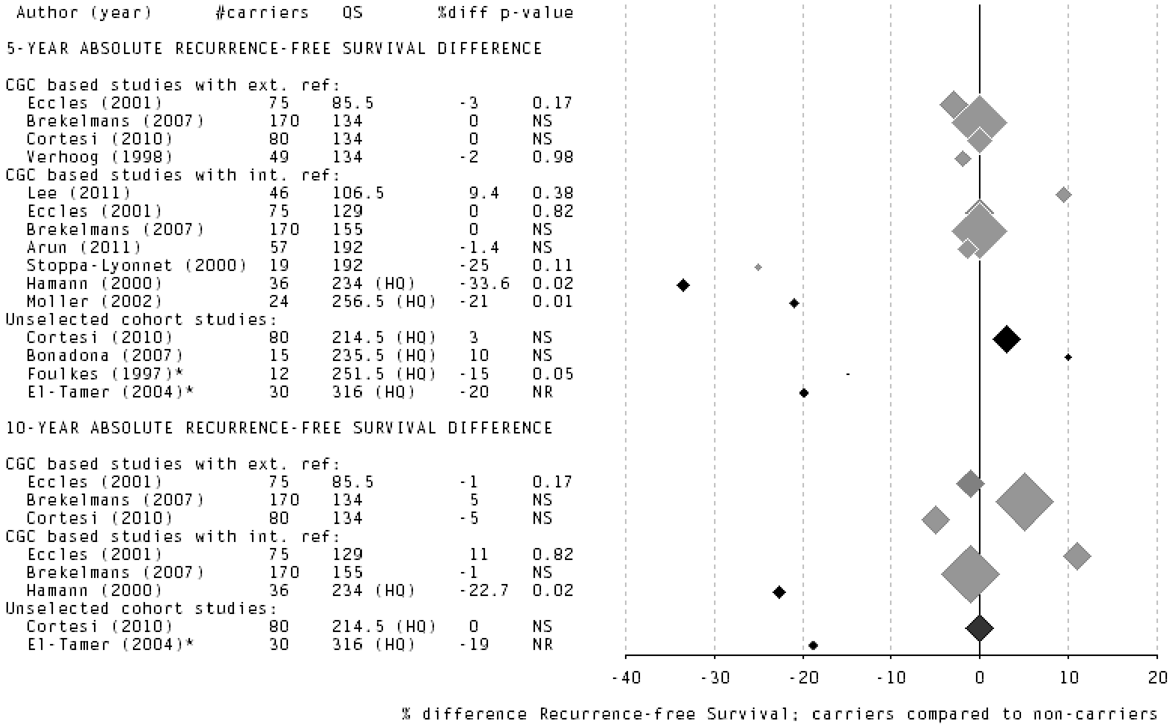

The forest plot above shows the absolute RFS differences of *BRCA1* compared to 'non-carriers' reported by studies included in this review. Three studies [5,6,8] (20%) reported a 3% to 10% better 5-year absolute RFS for *BRCA1* compared to non-carriers. In contrast, eight studies [12,13,16,17,21,23,34,35] (53%) reported a worse 5-year absolute RFS compared to 'non-carriers', with differences ranging from 1.4% to 33.6%; two of these studies [12,35] observed statistically significant results. Eight [6,12,16,17,20] of the 15 studies reporting a 5-year absolute RFS difference for *BRCA1* mutation carriers also reported the 10-year RFS difference. Five studies [6,12,16,17,20] (63%) reported a worse 10-year absolute RFS for *BRCA1* compared to 'non-carriers', with survival differences ranging from 1% to 22.7%. Only two studies [16,20] (25%) reported a better RFS of 5% and 10%.

Only two studies [20,21] reported HRs for RFS for *BRCA1* compared to 'non-carriers' (no figure shown). Brekelmans and colleagues [20] observed a non-significantly better unadjusted (HR 0.92, 95% CI 0.56-1.51) and adjusted (HR 0.84, 95% CI 0.41-1.75) RFS and Verhoog and colleagues [21] observed an equal unadjusted RFS (HR 1, 95% CI 0.65-1.55) and a worse adjusted RFS (HR 1.09, 95% CI 0.7-1.7) for *BRCA1* mutation carriers compared to 'non-carriers'.

References

1. Gaffney DK, Brohet RM, Lewis CM, Holden JA, Buys SS, et al. Response to radiation therapy and prognosis in breast cancer patients with BRCA1 and BRCA2 mutations. Radiother Oncol. 1998;47: 129-136.
2. Plakhins G, Irmejs A, Gardovskis A, Subatniece S, Liepniece-Karele I, et al. Underestimated survival predictions of the prognostic tools Adjuvant! Online and PREDICT in BRCA1-associated breast cancer patients. Fam Cancer. 2013.
3. Lee JS, Wacholder S, Struewing JP, McAdams M, Pee D, et al. Survival after breast cancer in Ashkenazi Jewish BRCA1 and BRCA2 mutation carriers. J Natl Cancer Inst. 1999;91: 259-263.
4. Einbeigi Z, Bergman A, Kindblom LG, Martinsson T, Meis-Kindblom JM, et al. A founder mutation of the BRCA1 gene in Western Sweden associated with a high incidence of breast and ovarian cancer. Eur J Cancer. 2001;37: 1904-1909.
5. Bonadona V, Dussart-Moser S, Voirin N, Sinilnikova OM, Mignotte H, et al. Prognosis of early-onset breast cancer based on BRCA1/2 mutation status in a French population-based cohort and review. Breast Cancer Res Treat. 2007;101: 233-245.
6. Cortesi L, Masini C, Cirilli C, Medici V, Marchi I, et al. Favourable ten-year overall survival in a Caucasian population with high probability of hereditary breast cancer. BMC Cancer. 2010;10: 90.
7. Hagen AI, Tretli S, Maehle L, Apold J, Veda N, et al. Survival in Norwegian BRCA1 mutation carriers with breast cancer. Hered Cancer Clin Pract. 2009;7: 7.
8. Lee LJ, Alexander B, Schnitt SJ, Comander A, Gallagher B, et al. Clinical outcome of triple negative breast cancer in BRCA1 mutation carriers and noncarriers. Cancer. 2011;117: 3093-3100.
9. Johannsson OT, Ranstam J, Borg A, Olsson H. Survival of BRCA1 breast and ovarian cancer patients: a population-based study from southern Sweden. J Clin Oncol. 1998;16: 397-404.
10. Huzarski T, Byrski T, Gronwald J, Gorski B, Domagala P, et al. Ten-Year Survival in Patients With BRCA1-Negative and BRCA1-Positive Breast Cancer. J Clin Oncol. 2013.
11. Ansquer Y, Gautier C, Fourquet A, Asselain B, Stoppa-Lyonnet D. Survival in early-onset BRCA1 breast-cancer patients. Institut Curie Breast Cancer Group. Lancet. 1998;352: 541.
12. Hamann U, Sinn HP. Survival and tumor characteristics of German hereditary breast cancer patients. Breast Cancer Res Treat. 2000;59: 185-192.
13. Stoppa-Lyonnet D, Ansquer Y, Dreyfus H, Gautier C, Gauthier-Villars M, et al. Familial invasive breast cancers: worse outcome related to BRCA1 mutations. J Clin Oncol. 2000;18: 4053-4059.
14. Goode EL, Dunning AM, Kuschel B, Healey CS, Day NE, et al. Effect of germ-line genetic variation on breast cancer survival in a population-based study. Cancer Res. 2002;62: 3052-3057.
15. Goffin JR, Chappuis PO, Begin LR, Wong N, Brunet JS, et al. Impact of germline BRCA1 mutations and overexpression of p53 on prognosis and response to treatment following breast carcinoma: 10-year follow up data. Cancer. 2003;97: 527-536.
16. Eccles D, Simmonds P, Goddard J, Coultas M, Hodgson S, et al. Familial breast cancer: an investigation into the outcome of treatment for early stage disease. Fam Cancer. 2001;1: 65-72.
17. El Tamer M, Russo D, Troxel A, Bernardino LP, Mazziotta R, et al. Survival and recurrence after breast cancer in BRCA1/2 mutation carriers. Ann Surg Oncol. 2004;11: 157-164.
18. Moller P, Evans DG, Reis MM, Gregory H, Anderson E, et al. Surveillance for familial breast cancer: Differences in outcome according to BRCA mutation status. Int J Cancer. 2007;121: 1017-1020.
19. Chiappetta G, Ottaiano A, Vuttariello E, Monaco M, Galdiero F, et al. HMGA1 protein expression in familial breast carcinoma patients. Eur J Cancer. 2010;46: 332-339.
20. Brekelmans CT, Tilanus-Linthorst MM, Seynaeve C, vd Ouweland A, Menke-Pluymers MB, et al. Tumour characteristics, survival and prognostic factors of hereditary breast cancer from BRCA2-, BRCA1- and non-BRCA1/2 families as compared to sporadic breast cancer cases. Eur J Cancer. 2007;43: 867-876.
21. Verhoog LC, Brekelmans CT, Seynaeve C, van den Bosch LM, Dahmen G, et al. Survival and tumour characteristics of breast-cancer patients with germline mutations of BRCA1. Lancet. 1998;351: 316-321.
22. Eerola H, Vahteristo P, Sarantaus L, Kyyronen P, Pyrhonen S, et al. Survival of breast cancer patients in BRCA1, BRCA2, and non-BRCA1/2 breast cancer families: a relative survival analysis from Finland. Int J Cancer. 2001;93: 368-372.
23. Arun B, Bayraktar S, Liu DD, Gutierrez Barrera AM, Atchley D, et al. Response to neoadjuvant systemic therapy for breast cancer in BRCA mutation carriers and noncarriers: a single-institution experience. J Clin Oncol. 2011;29: 3739-3746.
24. Goodwin PJ, Phillips KA, West DW, Ennis M, Hopper JL, et al. Breast cancer prognosis in BRCA1 and BRCA2 mutation carriers: an International Prospective Breast Cancer Family Registry population-based cohort study. J Clin Oncol. 2012;30: 19-26.
25. Robson M, Levin D, Federici M, Satagopan J, Bogolminy F, et al. Breast conservation therapy for invasive breast cancer in Ashkenazi women with BRCA gene founder mutations. J Natl Cancer Inst. 1999;91: 2112-2117.
26. Rennert G, Bisland-Naggan S, Barnett-Griness O, Bar-Joseph N, Zhang S, et al. Clinical outcomes of breast cancer in carriers of BRCA1 and BRCA2 mutations. N Engl J Med. 2007;357: 115-123.

27. Vinodkumar B, Syamala V, Abraham EK, Balakrishnan R, Ankathil R. Germline BRCA1 mutation and survival analysis in familial breast cancer patients in Kerala; South India. J Exp Clin Cancer Res. 2007;26: 329-336.
28. Seynaeve C, Verhoog LC, van de Bosch LM, van Geel AN, Menke-Pluymers M, et al. Ipsilateral breast tumour recurrence in hereditary breast cancer following breast-conserving therapy. Eur J Cancer. 2004;40: 1150-1158.
29. Plakhins G, Irmejs A, Gardovskis A, Subatniece S, Rozite S, et al. Genotype-phenotype correlations among BRCA1 4153delA and 5382insC mutation carriers from Latvia. BMC Med Genet. 2011;12: 147.
30. Xu J, Wang B, Zhang Y, Li R, Wang Y, et al. Clinical implications for BRCA gene mutation in breast cancer. Mol Biol Rep. 2012;39: 3097-3102.
31. Robson ME, Chappuis PO, Satagopan J, Wong N, Boyd J, et al. A combined analysis of outcome following breast cancer: differences in survival based on BRCA1/BRCA2 mutation status and administration of adjuvant treatment. Breast Cancer Res. 2004;6: R8-R17.
32. Heikkinen T, Karkkainen H, Aaltonen K, Milne RL, Heikkila P, et al. The breast cancer susceptibility mutation PALB2 1592delT is associated with an aggressive tumor phenotype. Clin Cancer Res. 2009;15: 3214-3222.
33. Chappuis PO, Donato E, Goffin JR, Wong N, Begin LR, et al. Cyclin E expression in breast cancer: predicting germline BRCA1 mutations, prognosis and response to treatment. Ann Oncol. 2005;16: 735-742.
34. Foulkes WD, Wong N, Brunet JS, Begin LR, Zhang JC, et al. Germ-line BRCA1 mutation is an adverse prognostic factor in Ashkenazi Jewish women with breast cancer. Clin Cancer Res. 1997;3: 2465-2469.
35. Moller P, Borg A, Evans DG, Haites N, Reis MM, et al. Survival in prospectively ascertained familial breast cancer: analysis of a series stratified by tumour characteristics, BRCA mutations and oophorectomy. Int J Cancer. 2002;101: 555-559.
