# Supplementary material for: Worse Breast Cancer Prognosis of BRCA1/BRCA2 Mutation Carriers: What's the Evidence? A Systematic Review with Meta-Analysis
Source: PLoS One. 2015 Mar 27;10(3):e0120189. doi: 10.1371/journal.pone.0120189 (PMC4376645; doi:10.1371/journal.pone.0120189)
Supplement: S5 Supporting Information — (PDF) [file pone.0120189.s005.pdf]

S5 Supporting Information. Results *BRCA2* mutation carriership.

Forest plots (the forest plots are also shown outside the Supporting information (Figs. 4A-D), but are repeated here for readability): size of the bullet represents the number of included carriers; black bullet = HQ study; round bullet (●) and * = A. Jewish study population, only founder mutations tested; square bullet (■) and ** = specific study population (but not A. Jewish), in which only founder mutations were tested; — = 95% Confidence interval (only for hazard ratios); CGC based studies with ext. ref. = CGC based studies with external reference group; CGC based studies with int. ref. = CGC based studies with internal reference group; Sign = statistically significant ($P < 0.05$); NS = not statistically significant; NR = not reported; †Adjusted for clinico-pathological characteristics and/or treatment.

A. *BRCA2* mutation carriership and overall survival (OS)

Absolute OS differences: *BRCA2* mutation carriers compared to 'non-carriers'

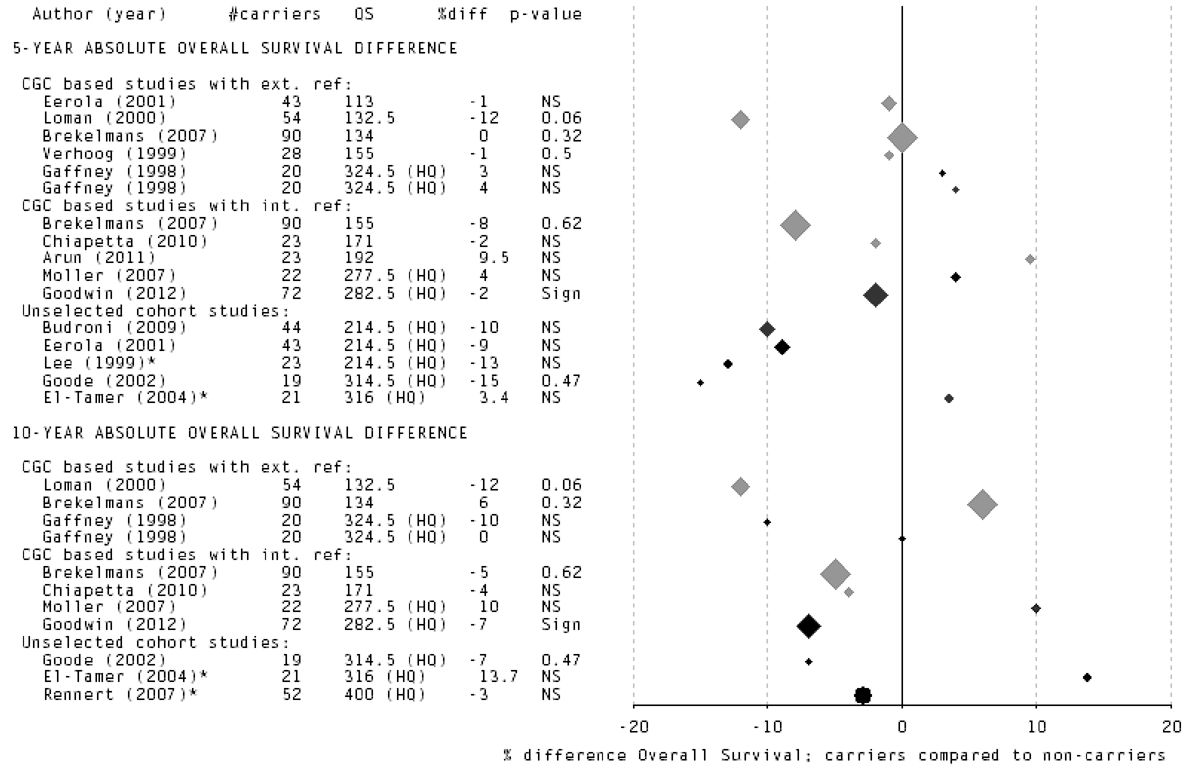

The forest plot above shows the absolute OS differences of *BRCA2* compared to 'non-carriers' reported by studies included in this review. Five studies [1-4] (31%) reported a better 5-year absolute OS for *BRCA2* compared to 'non-carriers', but four [1,2,4] of these studies reported survival differences smaller than 5%. On the other hand, ten studies [5-13] (63%) reported a worse 5-year absolute OS, with differences ranging from 1% to 15% including one [13] statistically significant difference of 2%. Three studies [2,3,12] (27%) reported a better 10-year absolute OS for *BRCA2* compared to 'non-carriers', with differences of 6% and 13.7 %. Comparable to the 5-year results, more studies (seven [1,7,9,11-14] (64%)) reported again a worse OS, with 10-year absolute OS differences ranging from 3% to 12%; including the same study [13] reporting a statistically significant difference of 7%.

Hazard ratios for OS: *BRCA2* mutation carriers compared to 'non-carriers'

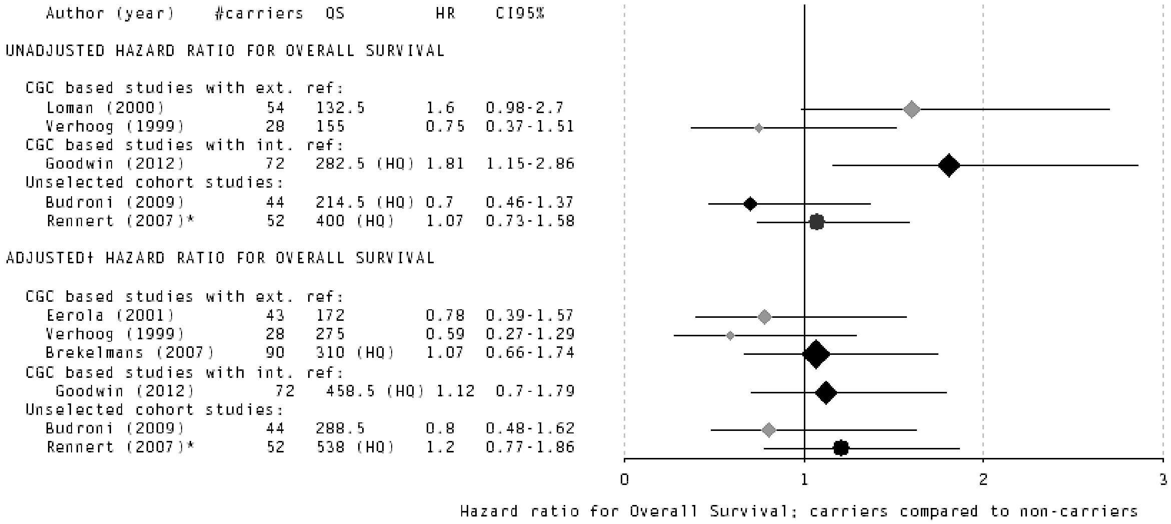

The forest plot above shows the seven studies [6-8,10,12-14] reporting adjusted and/or unadjusted HRs for OS for *BRCA2* compared to 'non-carriers'. Three [7,13,14] (60%) of the five studies [6,7,10,13,14] reporting an unadjusted HR found a worse OS for *BRCA2* mutation carriers; one study [14] only observed a slight increased HR (1.07), however, another study [13] observed a HR which was statistically significant. The other two studies [6,10] (40%) reported a non-significant better unadjusted OS for *BRCA2* mutation carriers. For the adjusted OS for *BRCA2* compared to 'non-carriers', three studies [6,8] (50%) reported a better adjusted OS, while three other studies [12,14] (50%) reported a worse OS. None of these results were statistically significant.

B. *BRCA2* mutation carriership and breast cancer-specific survival (BCSS)

Absolute BCSS differences: *BRCA2* mutation carriers compared to 'non-carriers'

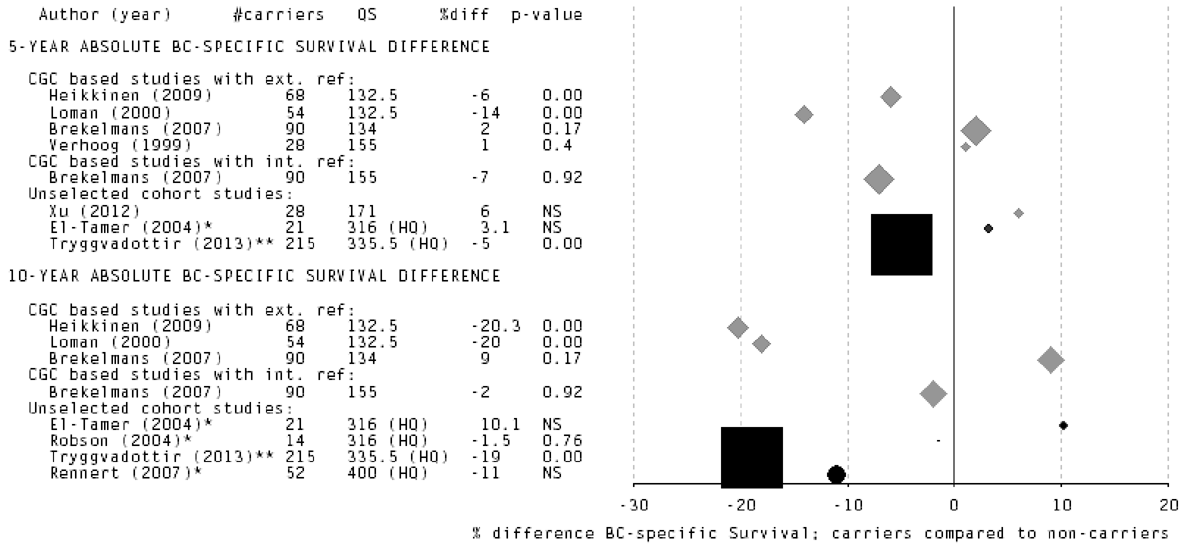

The forest plot above shows the absolute BCSS differences of *BRCA2* compared to 'non-carriers' reported by studies included in this review. Four studies [2,6,12,15] (50%) reported a better 5-year absolute BCSS for *BRCA2* compared to 'non-carriers', though all reported survival differences were less than 6%. The other four studies [7,12,16,17] (50%) reported a 5% to 14% worse 5-year BCSS for *BRCA2* mutation carriers, including three [7,16,17] statistically significant results. Only two studies [2,12] (25%) reported a better 10-year absolute BCSS for *BRCA2* compared to 'non-carriers' with absolute differences of 9% and 10.1%, while six studies [7,12,14,16-18] (75%) reported a worse 10-year BCSS for *BRCA2* mutation carriers, with differences ranging from 1.5% to 20.3 % including three [7,16,17] statistically significant results.

Hazard ratios for BCSS: *BRCA2* mutation carriers compared to 'non-carriers'

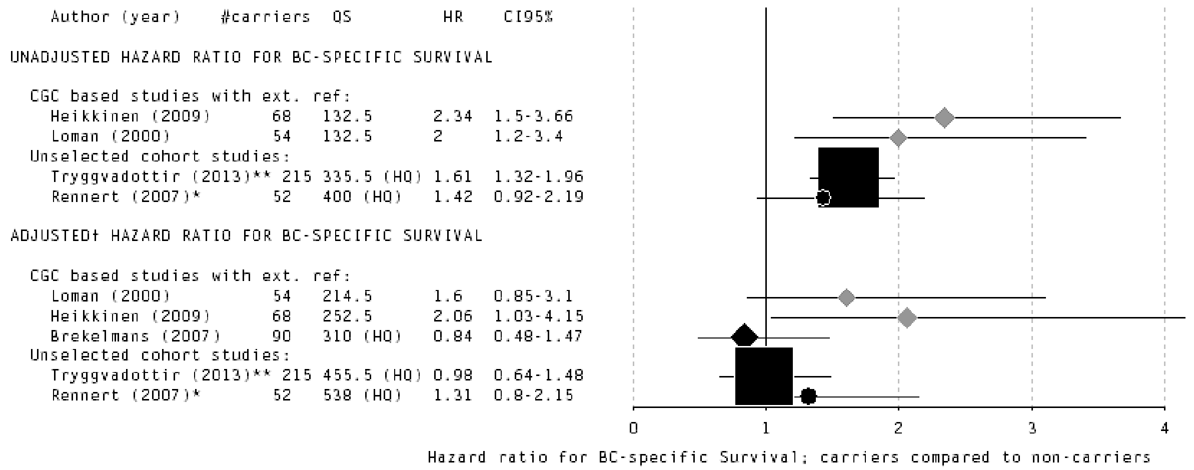

The forest plot above shows the univariate and multivariate hazard ratios for BCSS of *BRCA2* compared to 'non-carriers' reported by studies included in this review. For BCSS of *BRCA2* compared to 'non-carriers', only five studies [7,12,14,16,17] reported an HR; all four studies [7,14,16,17] reporting an unadjusted HR observed a worse BCSS for *BRCA2* mutation carriers, with HRs ranging from 1.42 to 2.34 and three [7,16,17] statistically significant results. On the other hand, three [7,14,17] (60%) of the five studies [7,12,14,16,17] reporting an adjusted HR found a worse adjusted BCSS for *BRCA2* compared to 'non-carriers'; also only one [17] of these studies reported a statistically significant result. Two studies [12,16] (40%) reported a better adjusted BCSS for *BRCA2* mutation carriers (HR 0.98 and 0.84).

C. *BRCA2* mutation carriership and metastasis-free survival (MFS)

There were only three studies [12,13] which reported the 5 and 10-year absolute MFS differences for *BRCA2* compared to 'non-carriers' (no figures shown). One substudy by Brekelmans and colleagues [12] did not find any difference, though the other substudy [12] reported a respectively 9% and 11% 5- and 10-year better absolute MFS for *BRCA2* mutation carriers. The HQ study by Goodwin and colleagues [13] reported a statistically significant 11% and 6% 5- and 10-year worse absolute MFS for *BRCA2* mutation carriers and also a statistically significant unadjusted HR of 1.63 (95% CI 1.02-2.6). In contrast, the adjusted HR which this study [13] reported was 1 (95% CI 0.62-1.61). The adjusted HR reported in the substudy of Brekelmans and colleagues [12] was 0.75 (95% CI 0.44-1.29).

D. *BRCA2* mutation carriership and recurrence-free survival (RFS)

Five studies [2,4,6,12] (one HQ study [2]) reported 5-year absolute RFS differences for *BRCA2* compared to 'non-carriers' (no figure shown); three studies [2,12] (60%) observed a 5% to 9% worse absolute RFS for *BRCA2* mutation carriers. The substudies of Brekelmans and colleagues [12] also reported 10-year absolute RFS differences, i.e. 4% (better survival) and 2% (worse survival) for *BRCA2* compared to 'non-carriers'. On the other hand, Arun and colleagues [4] observed a 19.4% better 5-year absolute RFS for *BRCA2* mutation carriers. Another study by Verhoog and colleagues [6] did not find any difference in 5-year absolute RFS between *BRCA2* and 'non-carriers', and additionally reported an unadjusted HR of 0.92 (95% CI 0.52-1.63). Adjustment of this HR made it stronger (HR 0.84, 95% CI 0.44-1.63). One substudy of Brekelmans and colleagues [12] also reported an adjusted better RFS for *BRCA2* mutation carriers (HR 0.85, 95% CI 0.26-2.77). None of the above results were significant.

References

1. Gaffney DK, Brohet RM, Lewis CM, Holden JA, Buys SS, et al. Response to radiation therapy and prognosis in breast cancer patients with BRCA1 and BRCA2 mutations. Radiother Oncol. 1998;47: 129-136.
2. El Tamer M, Russo D, Troxel A, Bernardino LP, Mazziotta R, et al. Survival and recurrence after breast cancer in BRCA1/2 mutation carriers. Ann Surg Oncol. 2004;11: 157-164.
3. Moller P, Evans DG, Reis MM, Gregory H, Anderson E, et al. Surveillance for familial breast cancer: Differences in outcome according to BRCA mutation status. Int J Cancer. 2007;121: 1017-1020.
4. Arun B, Bayraktar S, Liu DD, Gutierrez Barrera AM, Atchley D, et al. Response to neoadjuvant systemic therapy for breast cancer in BRCA mutation carriers and noncarriers: a single-institution experience. J Clin Oncol. 2011;29: 3739-3746.
5. Lee JS, Wacholder S, Struewing JP, McAdams M, Pee D, et al. Survival after breast cancer in Ashkenazi Jewish BRCA1 and BRCA2 mutation carriers. J Natl Cancer Inst. 1999;91: 259-263.
6. Verhoog LC, Brekelmans CT, Seynaeve C, Dahmen G, van Geel AN, et al. Survival in hereditary breast cancer associated with germline mutations of BRCA2. J Clin Oncol. 1999;17: 3396-3402.
7. Loman N, Johannsson O, Bendahl P, Dahl N, Einbeigi Z, et al. Prognosis and clinical presentation of BRCA2-associated breast cancer. Eur J Cancer. 2000;36: 1365-1373.
8. Eerola H, Vahteristo P, Sarantaus L, Kyyronen P, Pyrhonen S, et al. Survival of breast cancer patients in BRCA1, BRCA2, and non-BRCA1/2 breast cancer families: a relative survival analysis from Finland. Int J Cancer. 2001;93: 368-372.
9. Goode EL, Dunning AM, Kuschel B, Healey CS, Day NE, et al. Effect of germ-line genetic variation on breast cancer survival in a population-based study. Cancer Res. 2002;62: 3052-3057.
10. Budroni M, Cesaraccio R, Coviello V, Sechi O, Pirino D, et al. Role of BRCA2 mutation status on overall survival among breast cancer patients from Sardinia. BMC Cancer. 2009;9: 62.
11. Chiappetta G, Ottaiano A, Vuttariello E, Monaco M, Galdiero F, et al. HMGA1 protein expression in familial breast carcinoma patients. Eur J Cancer. 2010;46: 332-339.
12. Brekelmans CT, Tilanus-Linthorst MM, Seynaeve C, vd Ouweland A, Menke-Pluymers MB, et al. Tumour characteristics, survival and prognostic factors of hereditary breast cancer from BRCA2-, BRCA1- and non-BRCA1/2 families as compared to sporadic breast cancer cases. Eur J Cancer. 2007;43: 867-876.
13. Goodwin PJ, Phillips KA, West DW, Ennis M, Hopper JL, et al. Breast cancer prognosis in BRCA1 and BRCA2 mutation carriers: an International Prospective Breast Cancer Family Registry population-based cohort study. J Clin Oncol. 2012;30: 19-26.
14. Rennert G, Bisland-Naggan S, Barnett-Griness O, Bar-Joseph N, Zhang S, et al. Clinical outcomes of breast cancer in carriers of BRCA1 and BRCA2 mutations. N Engl J Med. 2007;357: 115-123.
15. Xu J, Wang B, Zhang Y, Li R, Wang Y, et al. Clinical implications for BRCA gene mutation in breast cancer. Mol Biol Rep. 2012;39: 3097-3102.
16. Tryggvadottir L, Olafsdottir EJ, Olafsdottir GH, Sigurdsson H, Johannsson OT, et al. Tumour diploidy and survival in breast cancer patients with BRCA2 mutations. Breast Cancer Res Treat. 2013;140: 375-384.
17. Heikkinen T, Karkkainen H, Aaltonen K, Milne RL, Heikkila P, et al. The breast cancer susceptibility mutation PALB2 1592delT is associated with an aggressive tumor phenotype. Clin Cancer Res. 2009;15: 3214-3222.
18. Robson ME, Chappuis PO, Satagopan J, Wong N, Boyd J, et al. A combined analysis of outcome following breast cancer: differences in survival based on BRCA1/BRCA2 mutation status and administration of adjuvant treatment. Breast Cancer Res. 2004;6: R8-R17.
