# Supplementary material for: Worse Breast Cancer Prognosis of BRCA1/BRCA2 Mutation Carriers: What's the Evidence? A Systematic Review with Meta-Analysis
Source: PLoS One. 2015 Mar 27;10(3):e0120189. doi: 10.1371/journal.pone.0120189 (PMC4376645; doi:10.1371/journal.pone.0120189)
Supplement: S6 Supporting Information — (PDF) [file pone.0120189.s006.pdf]

S6 Supporting Information. Forest plots of high quality (HQ) studies, based on the Random effect (DerSimonian and Laird) analyses.

Included in the forest plots are the HQ studies reporting hazard ratios for overall survival (panels A, B and G), breast cancer-specific survival (panels C and D and H) and metastasis-free survival (panels e and f) of *BRCA1* (panels A-F) or *BRCA2* (panel G and H) mutation carriers compared to ‘non-carriers’.
The results for each type of survival outcome are stratified per reported risk estimate: the unadjusted hazard ratios (panels A, C, E, G and H) and adjusted hazard ratios (panels B, D and F).
Shown are the results of the Random effect (DerSimonian and Laird) analyses [1].

A. Only HQ studies: Unadjusted hazard ratios for overall survival, *BRCA1* mutation carriers compared to ‘non-carriers’.

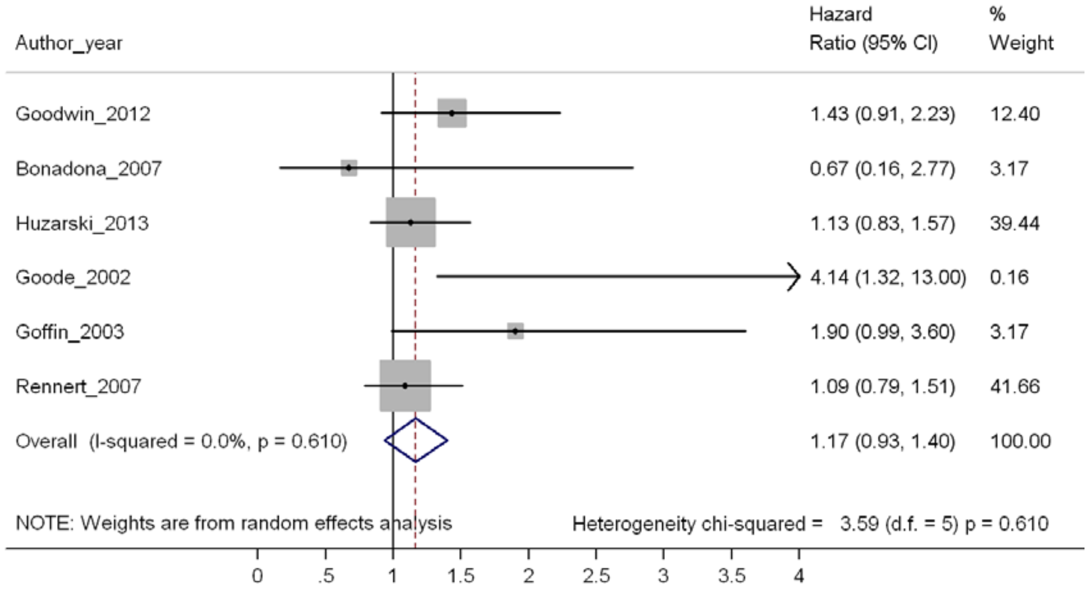

B. Only HQ studies: adjusted hazard ratios for overall survival, *BRCA1* mutation carriers compared to ‘non-carriers’.

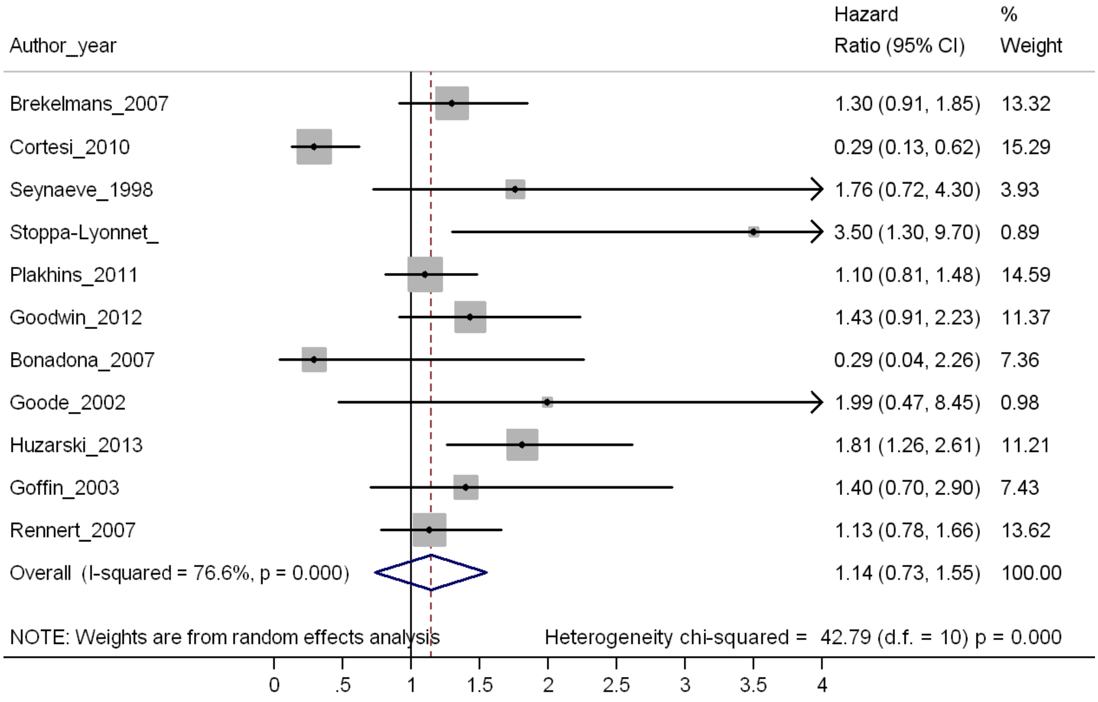

C. Only HQ studies: Unadjusted hazard ratios for breast cancer-specific survival, *BRCA1* mutation carriers compared to ‘non-carriers’.

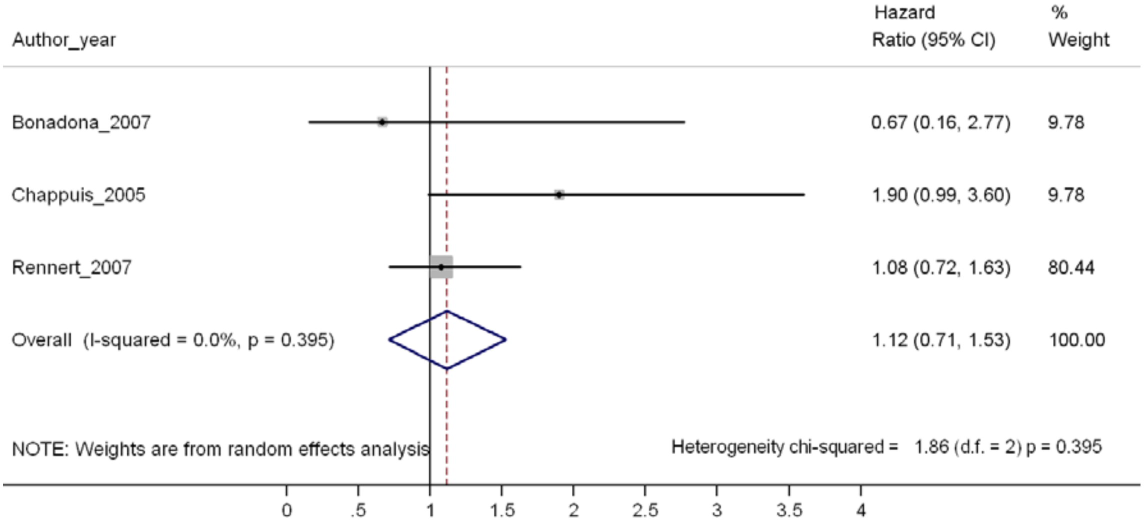

D. Only HQ studies: adjusted hazard ratios for breast cancer-specific survival, *BRCA1* mutation carriers compared to ‘non-carriers’.

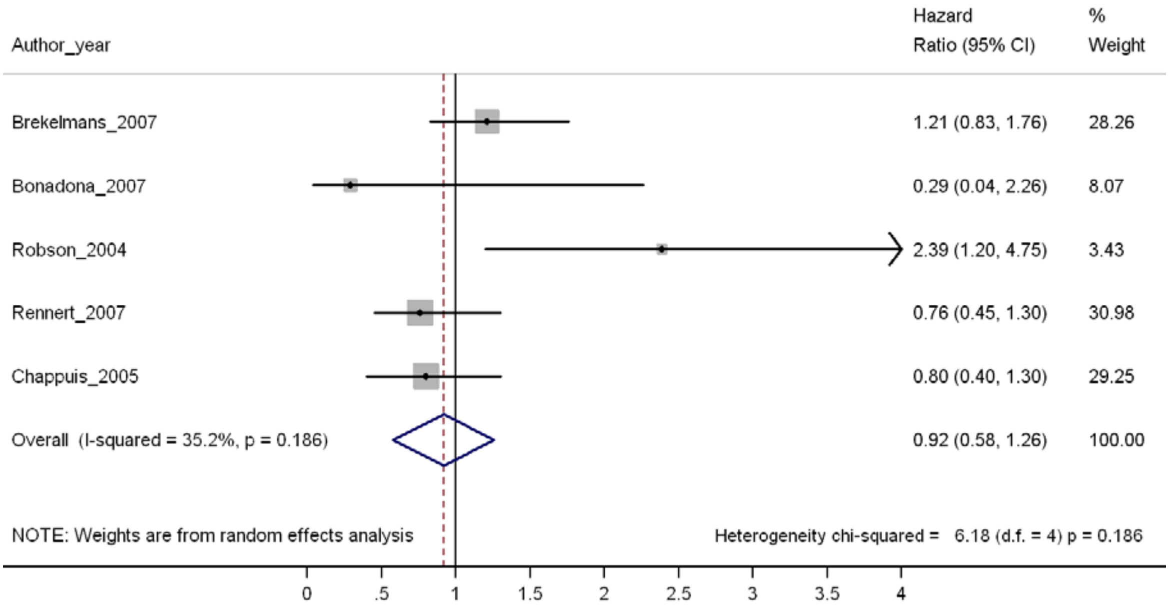

E. Only HQ studies: Unadjusted hazard ratios for metastasis-free survival, *BRCA1* mutation carriers compared to 'non-carriers'.

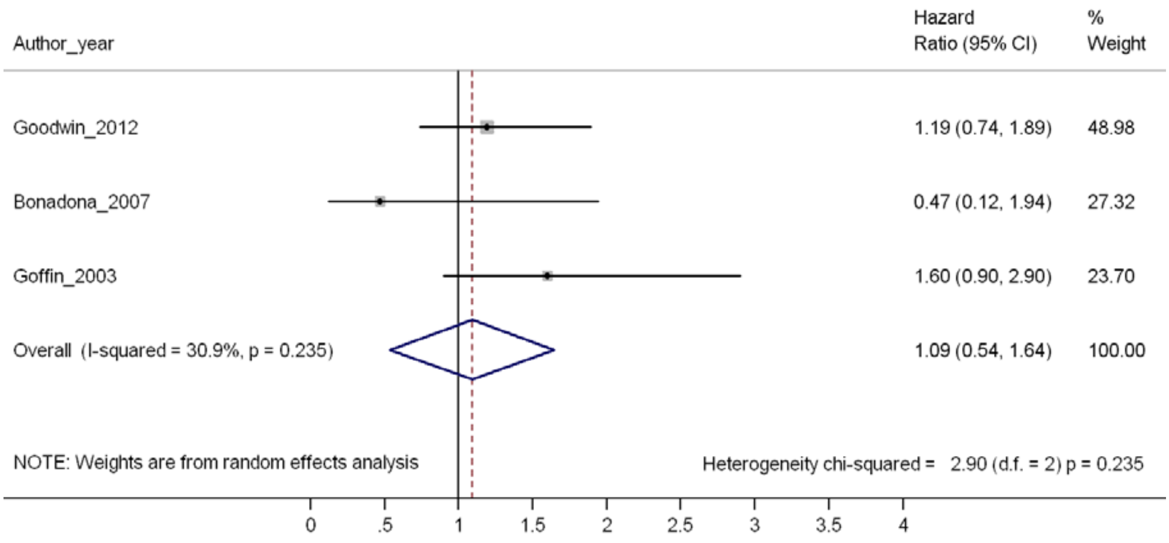

F. Only HQ studies: Adjusted hazard ratios for metastasis-free survival, *BRCA1* mutation carriers compared to 'non-carriers'.

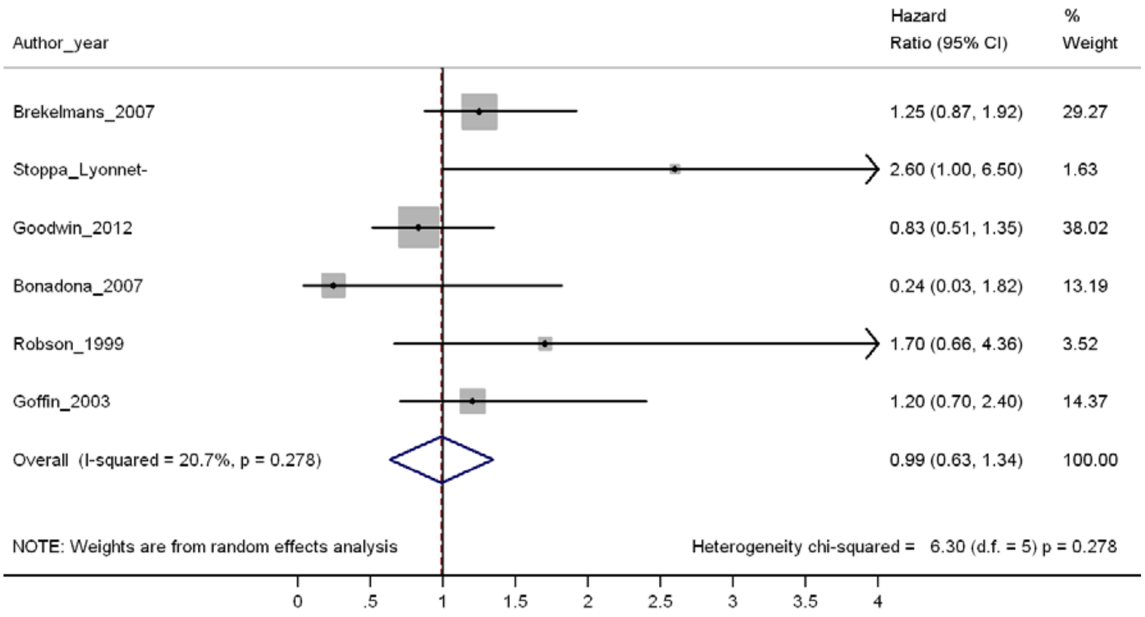

G. Only HQ studies: Unadjusted hazard ratios for overall survival, *BRCA2* mutation carriers compared to 'non-carriers'.

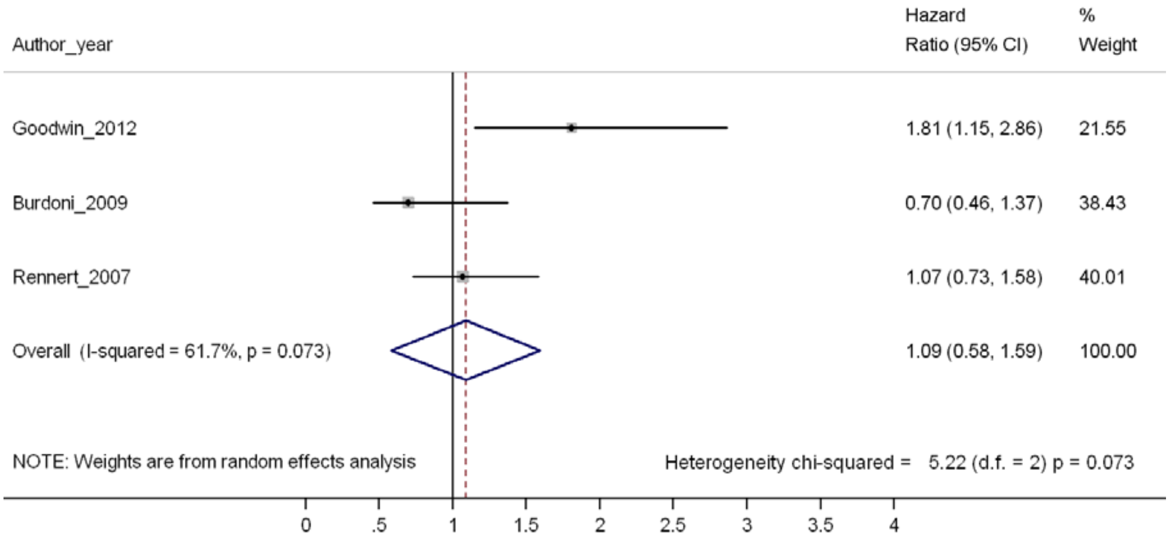

H. Only HQ studies: Unadjusted hazard ratios for breast cancer-specific survival, *BRCA2* mutation carriers compared to 'non-carriers'.

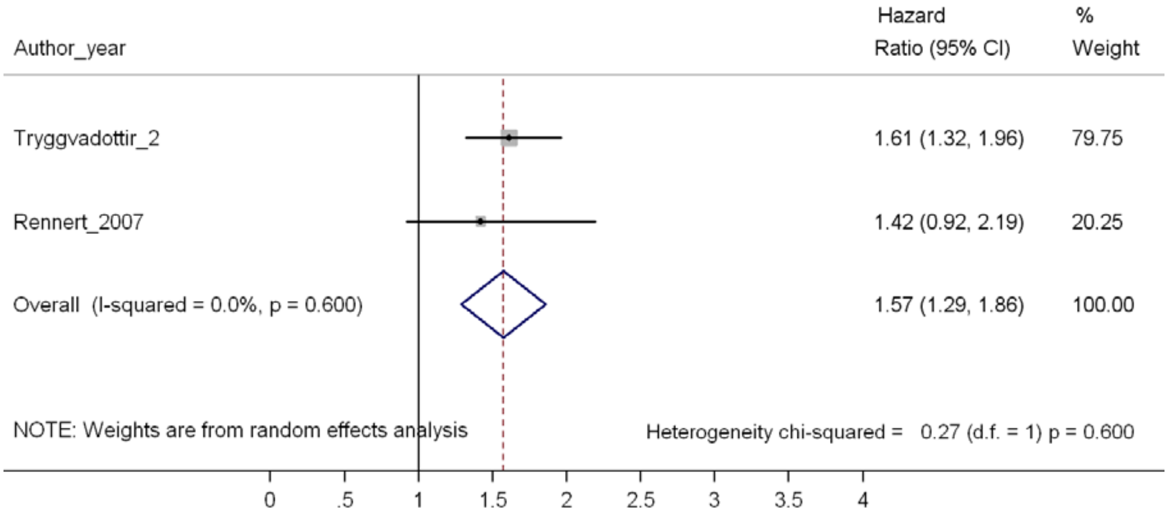

References

1. Dersimonian R, Laird N (1986) Meta-analysis in clinical trials. Control Clin Trials 7: 177-188.
