# Supplementary material for: Worse Breast Cancer Prognosis of BRCA1/BRCA2 Mutation Carriers: What's the Evidence? A Systematic Review with Meta-Analysis
Source: PLoS One. 2015 Mar 27;10(3):e0120189. doi: 10.1371/journal.pone.0120189 (PMC4376645; doi:10.1371/journal.pone.0120189)
Supplement: S7 Supporting Information — (PDF) [file pone.0120189.s007.pdf]

S7 Supporting Information. Results *BRCA1* and *BRCA2* mutation carriership combined.

Forest plots: size of the bullet represents the number of included carriers; black bullet = HQ study; round bullet (●) and * = A. Jewish study population, only founder mutations tested; square bullet (■) and ** = specific study population (but not A. Jewish), in which only founder mutations were tested; — = 95% Confidence interval (only for hazard ratios); CGC based studies with ext. ref. = CGC based studies with external reference group; CGC based studies with int. ref. = CGC based studies with internal reference group; Sign = statistically significant ($P < 0.05$); NS = not statistically significant; NR = not reported; †Adjusted for clinico-pathological characteristics and/or treatment.

A. *BRCA1/2* mutation carriership and overall survival (OS)

Absolute OS differences: *BRCA1/2* mutation carriers compared to 'non-carriers'

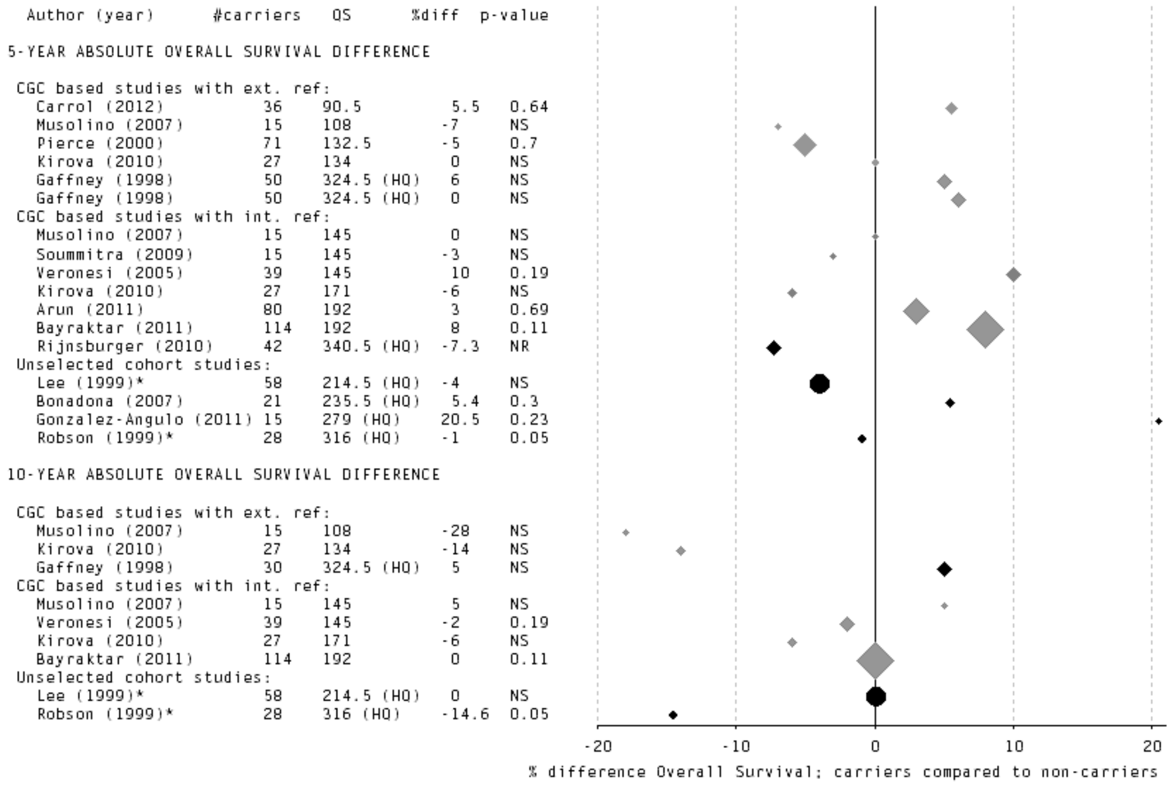

The forest plot above shows the absolute OS differences of *BRCA1/2* compared to 'non-carriers' reported by studies included in this review. Of the 17 studies [1-14] reporting the absolute 5-year OS difference for *BRCA1/2* compared to 'non-carriers', eight studies [1,5,6,11-14] (17%) reported a better OS (ranging from 3% to 20.5% difference) and 7 studies [2-4,7-10] (41%) reported a OS with survival differences ranging from 1% to 7.3%. Two studies [1,7] (22%) reported a 5% better 10-year OS. In contrast, five studies [3,5,7,8] (55%) reported a worse absolute 10-year survival for *BRCA1/2* compared to 'non-carriers' with differences ranging from 2% to 18%. Only one study [3], reporting both a worse 5-year and 10-year overall survival, observed statistically significant results.

Hazard ratios for OS: *BRCA1/2* mutation carriers compared to 'non-carriers'

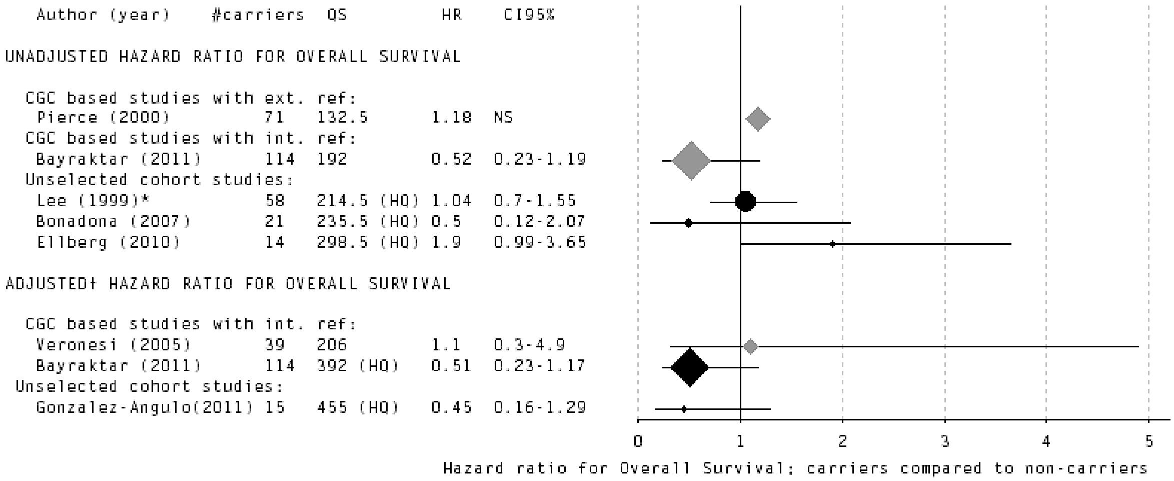

The forest plot above shows the seven studies [2,4-6,11,13,15] reporting the univariate and/or multivariate hazard ratios for OS of *BRCA1/2* compared to 'non-carriers'; the reported unadjusted hazard ratios ranged from 0.5 to 1.9 and the adjusted hazard ratios from 0.51 to 1.1. Only one study [13] reported both an unadjusted and adjusted hazard ratio (HR 0.52 and 0.51 respectively).

B. *BRCA1/2* mutation carriership and breast cancer-specific survival (BCSS)

Three studies [3,4,6] reported the absolute 5-year BCSS for *BRCA1/2* compared to 'non-carriers'; two studies [4,6] (= 1 HQ study [6]) reported a non-significant better BCSS survival for *BRCA1/2* mutation carriers with survival differences of 5.4% and 1%. The other HQ study [3] reported a statistically significant 10.6% worse BCSS for *BRCA1/2* mutation carriers. Additionally, this study reported a 15.3% worse absolute 10-year BCSS for *BRCA1/2* compared to non-carriers. Also another HQ study [16] reported a statistically significant 19% worse 10-year survival for *BRCA1/2* mutation carriers
Of the three studies [4,6,17] reporting an unadjusted hazard ratio for BCSS for *BRCA1/2* compared to 'non-carriers', two studies [4,6] reported hazard ratios under one (0.50, 95% CI 0.12-2.07 and 0.71 (no 95% CI reported) respectively) and one study [17] (= HQ study) reported a hazard ratio of 1.8 (95% CI 0.96-3.2). This study also reported an adjusted hazard ratio of 1.1 (95% CI 0.6-2). Furthermore, the other study [3] reporting an adjusted hazard ratio found a more clear worse BCSS for *BRCA1/2* mutation carriers (HR 2.08, 95% CI 0.79-5.44). None of these hazard ratios were statistically significant.

C. *BRCA1/2* mutation carriership and metastasis-free survival (MFS)

There were only four studies [3,6,9,18] (all HQ) looking at the relation between *BRCA1/2* mutation carriership and MFS. All studies reported the absolute 5-year MFS difference; one study [6] reported a 16.5% better MFS, while the other three studies [3,9,18] reported a 16.1% to 24% worse MFS for *BRCA1/2* compared to non-carriers. Two [3,18] of these differences were statistically significant. One of these studies [3] additionally reported a 18.1% worse absolute 10-year MFS for *BRCA1/2* mutation carrier, though, after adjustment for confounders the hazard ratio reported by the same study was not significant anymore (HR 1.45, 95% CI 0.6-3.49). Another study [18] additionally reported an unadjusted hazard ratio of 2.7 (95% CI 1.4-2.5), and also reported a statistically significant hazard ratio after adjustment (HR 2.1, 95% CI 1-4.3)). The study [6] reporting a better 5-year MFS, reported a non-significant unadjusted hazard ratio of 0.37 (95% CI 0.09-1.51).

D. *BRCA1/2* mutation carriership and recurrence-free survival (RFS)

Absolute RFS differences: *BRCA1/2* mutation carriers compared to 'non-carriers'

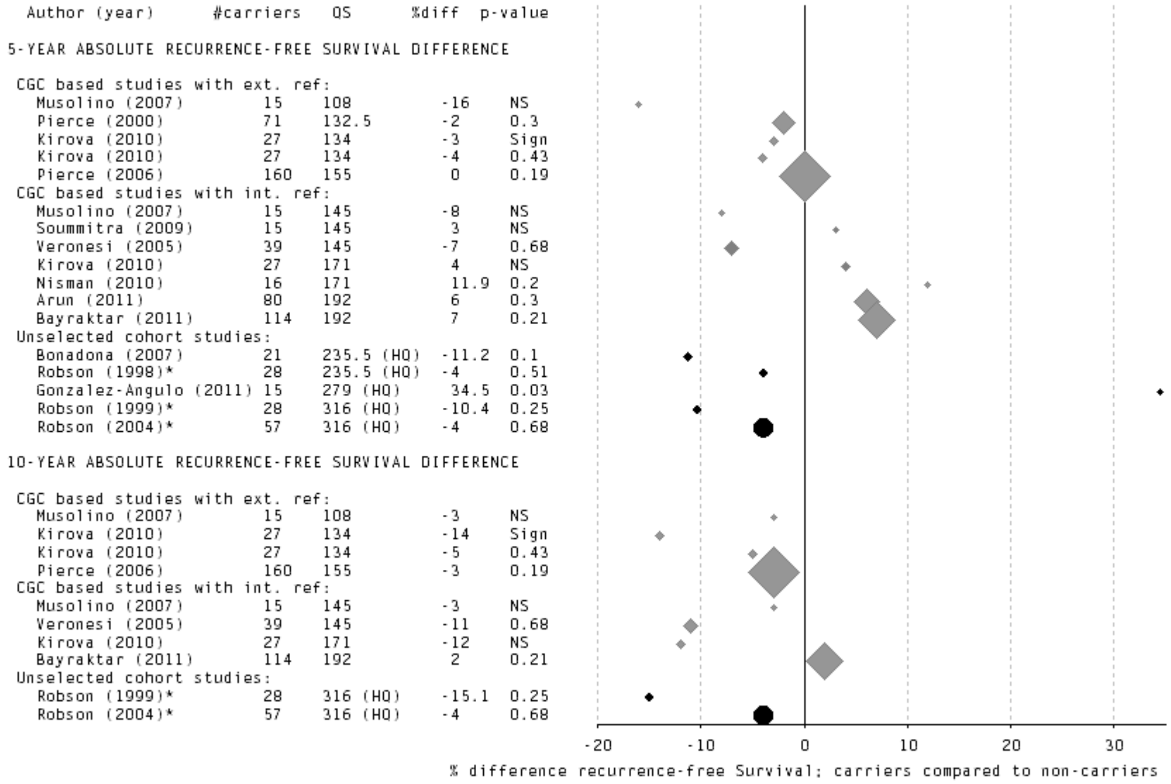

The forest plot above shows the absolute RFS differences of *BRCA1/2* compared to 'non-carriers' reported by studies included in this review. In contrast to metastasis-free survival, many studies [3-8,11,13,14,16,19-21] determined the association between *BRCA1/2* mutation carriership and RFS. There were 17 studies [3-8,11,13,14,16,19-21] reporting the 5-year absolute RFS difference for *BRCA1/2* compared to 'non-carriers'; most of the studies [3-8,16,22] (10 (59%)) reported a worse RFS for *BRCA1/2* mutation carriers, with survival differences ranging from 2% to 16% and one [8] statistically significant results. Though, there were also six studies [8,10,11,13,14,21] (35%) reporting a 3% to 34.5% better 5-year absolute RFS for *BRCA1/2* mutation carriers, with one study [11] reporting a statistically significant survival benefit of 34.5%. Interestingly, 10 [3,5,7,8,13,14,16,20] of the 17 [3-8,11,13,14,16,19-21] studies reporting the absolute 5-year RFS difference also reported a 10-year absolute RFS difference for *BRCA1/2* compared to 'non-carriers'; all these studies (90%) except one [13] reported a worse 10-year RFS for *BRCA1/2* mutation carriers with differences ranging from 3% to 15.1% including one [8] statistically significant survival difference of 14%.

Hazard ratios for RFS: *BRCA1/2* mutation carriers compared to 'non-carriers'

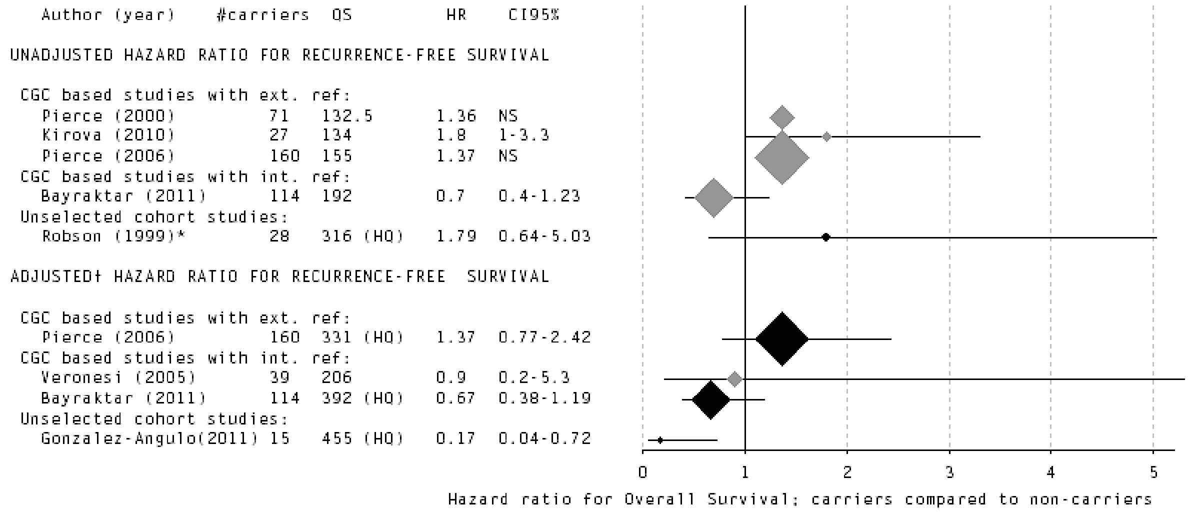

The forest plot above shows the univariate and multivariate hazard ratios for RFS of *BRCA1/2* compared to 'non-carriers' reported by studies included in this review. Five studies [3,4,8,13,20] reported an unadjusted hazard ratio; four [3,4,8,20] (80%) reported hazard ratios above 1 (ranging from 1.36 to 1.8), relating to a worse recurrence-free survival for *BRCA1/2* mu compared to 'non-carriers'. There were four studies [5,11,13,20] reporting an adjusted hazard ratio for RFS of *BRCA1/2* compared to 'non-carriers'; one study [20] (= HQ study) observed a hazard ratio of 1.37, while the other three studies [5,11,13] reported hazard ratios between 0.17 and 0.9, of which one [11] was statistically significant.

E. Best-evidence synthesis for the association between *BRCA1/2* mutation carriership and survival

Type of survival	Unadjusted/ adjusted[a]	Studies reporting a worse survival[b] % (n / total n)		Studies reporting a better survival[c] % (n / total n)		Evidence[d] (based on all studies)	Evidence[d] (based on HQ studies)
		Low quality	High quality	Low quality	High quality		
Overall	Unadjusted	30 (3/10)	**25 (2/8)**	14 (1/7)	**20 (2/10)**	Nil	**Nil**
	Adjusted	0 (0/1)	0 (0/2)	0 (0/1)	100 (2/2)	Indecisive*	Indecisive*
BC-specific	Unadjusted	0 (0/1)	**75 (3/4)**	100 (1/1)	**25 (1/4)**	Indecisive	**Indecisive**
	Adjusted	NA	50 (1/2)	NA	0 (0/2)	Indecisive*	Indecisive*
Metastasis-free	Unadjusted	NA	**75 (3/4)**	NA	**25 (1/4)**	Indecisive	**Indecisive**
	Adjusted	NA	100 (2/2)	NA	0 (0/2)	Indecisive*	Indecisive*
Recurrence-free	Unadjusted	50 (6/12)	**40 (2/5)**	17 (2/12)	**20 (1/5)**	Indecisive	**Indecisive**
	Adjusted	0 (0/1)	33 (1/3)	0 (0/1)	67 (2/3)	Nil	Indecisive*

[a]Adjusted survival is based on risk estimates adjusted for clinico-pathological characteristics and/or treatment; [b]Worse survival for univariate (unadjusted) outcomes: unadjusted HR >= 1.14 or 5-year absolute survival difference >= 10% or 10-year absolute survival difference >= 10% (when the 5 and 10 year survival differences go in opposite directions, we decided there was no difference in survival). Worse survival for multivariate (adjusted) outcomes: adjusted HR >= 1.14; [c]Better survival for univariate (unadjusted) outcomes: unadjusted HR <= 0.88 or 5-year absolute survival difference >= 10% or 10-year absolute survival difference >= 10% (when the 5 and 10 year survival differences go in opposite directions, we decided there was no difference in survival). Better survival for multivariate (adjusted) outcomes: adjusted HR <= 0.88; [d]See S2 Supporting Information (Best-evidence synthesis). Strong evidence: more than 75% of the HQ studies reported a worse survival; moderate evidence: 60-75% of the HQ studies reported a worse survival and less than 25% of the HQ studies reported a better survival / 50-60% of the HQ studies reported a worse survival and less than 10% of the HQ studies reported a better survival; nil evidence: more than 60% of the HQ studies reported a better survival or no association / more than 40% of the HQ studies reported a better survival; indecisive e evidence: all other options / less than four HQ studies available (*).

References

1. Gaffney DK, Brohet RM, Lewis CM, Holden JA, Buys SS, et al. Response to radiation therapy and prognosis in breast cancer patients with BRCA1 and BRCA2 mutations. Radiother Oncol. 1998;47: 129-136.
2. Lee JS, Wacholder S, Struewing JP, McAdams M, Pee D, et al. Survival after breast cancer in Ashkenazi Jewish BRCA1 and BRCA2 mutation carriers. J Natl Cancer Inst. 1999;91: 259-263.
3. Robson M, Levin D, Federici M, Satagopan J, Bogolminy F, et al. Breast conservation therapy for invasive breast cancer in Ashkenazi women with BRCA gene founder mutations. J Natl Cancer Inst. 1999;91: 2112-2117.
4. Pierce LJ, Strawderman M, Narod SA, Oliviotto I, Eisen A, et al. Effect of radiotherapy after breast-conserving treatment in women with breast cancer and germline BRCA1/2 mutations. J Clin Oncol. 2000;18: 3360-3369.
5. Veronesi A, de Giacomi C, Magri MD, Lombardi D, Zanetti M, et al. Familial breast cancer: characteristics and outcome of BRCA 1-2 positive and negative cases. BMC Cancer. 2005;5: 70.
6. Bonadona V, Dussart-Moser S, Voirin N, Sinilnikova OM, Mignotte H, et al. Prognosis of early-onset breast cancer based on BRCA1/2 mutation status in a French population-based cohort and review. Breast Cancer Res Treat. 2007;101: 233-245.
7. Musolino A, Bella MA, Bortesi B, Michiara M, Naldi N, et al. BRCA mutations, molecular markers, and clinical variables in early-onset breast cancer: a population-based study. Breast. 2007;16: 280-292.
8. Kirova YM, Savignoni A, Sigal-Zafrani B, de La Rochefordiere A, Salmon RJ, et al. Is the breast-conserving treatment with radiotherapy appropriate in BRCA1/2 mutation carriers? Long-term results and review of the literature. Breast Cancer Res Treat. 2010;120: 119-126.
9. Rijnsburger AJ, Obdeijn IM, Kaas R, Tilanus-Linthorst MM, Boetes C, et al. BRCA1-associated breast cancers present differently from BRCA2-associated and familial cases: long-term follow-up of the Dutch MRISC Screening Study. J Clin Oncol. 2010;28: 5265-5273.
10. Soumittra N, Meenakumari B, Parija T, Sridevi V, Nancy KN, et al. Molecular genetics analysis of hereditary breast and ovarian cancer patients in India. Hered Cancer Clin Pract. 2009;7: 13.
11. Gonzalez-Angulo AM, Timms KM, Liu S, Chen H, Litton JK, et al. Incidence and outcome of BRCA mutations in unselected patients with triple receptor-negative breast cancer. Clin Cancer Res. 2011;17: 1082-1089.
12. Carroll PA, Nolan C, Clarke R, Farrell M, Gleeson N, et al. Surgical management of an Irish cohort of BRCA-mutation carriers. Breast. 2011;20: 419-423.
13. Bayraktar S, Gutierrez-Barrera AM, Liu D, Tasbas T, Akar U, et al. Outcome of triple-negative breast cancer in patients with or without deleterious BRCA mutations. Breast Cancer Res Treat. 2011;130: 145-153.
14. Arun B, Bayraktar S, Liu DD, Gutierrez Barrera AM, Atchley D, et al. Response to neoadjuvant systemic therapy for breast cancer in BRCA mutation carriers and noncarriers: a single-institution experience. J Clin Oncol. 2011;29: 3739-3746.
15. Ellberg C, Jonsson G, Olsson H. Can a phenotype for recessive inheritance in breast cancer be defined? Fam Cancer. 2010;9: 525-530.
16. Robson ME, Chappuis PO, Satagopan J, Wong N, Boyd J, et al. A combined analysis of outcome following breast cancer: differences in survival based on BRCA1/BRCA2 mutation status and administration of adjuvant treatment. Breast Cancer Res. 2004;6: R8-R17.
17. Goffin JR, Straume O, Chappuis PO, Brunet JS, Begin LR, et al. Glomeruloid microvascular proliferation is associated with p53 expression, germline BRCA1 mutations and an adverse outcome following breast cancer. Br J Cancer. 2003;89: 1031-1034.
18. Chappuis PO, Kapusta L, Begin LR, Wong N, Brunet JS, et al. Germline BRCA1/2 mutations and p27(Kip1) protein levels independently predict outcome after breast cancer. J Clin Oncol. 2000;18: 4045-4052.
19. Verhoog LC, Brekelmans CT, Seynaeve C, van den Bosch LM, Dahmen G, et al. Survival and tumour characteristics of breast-cancer patients with germline mutations of BRCA1. Lancet. 1998;351: 316-321.
20. Pierce LJ, Levin AM, Rebbeck TR, Ben David MA, Friedman E, et al. Ten-year multi-institutional results of breast-conserving surgery and radiotherapy in BRCA1/2-associated stage I/II breast cancer. J Clin Oncol. 2006;24: 2437-2443.
21. Nisman B, Allweis T, Kaduri L, Maly B, Gronowitz S, et al. Serum thymidine kinase 1 activity in breast cancer. Cancer Biomark. 2010;7: 65-72.
22. Robson M, Gilewski T, Haas B, Levin D, Borgen P, et al. BRCA-associated breast cancer in young women. J Clin Oncol. 1998;16: 1642-1649.
