# Supplementary material for: Worse Breast Cancer Prognosis of BRCA1/BRCA2 Mutation Carriers: What's the Evidence? A Systematic Review with Meta-Analysis
Source: PLoS One. 2015 Mar 27;10(3):e0120189. doi: 10.1371/journal.pone.0120189 (PMC4376645; doi:10.1371/journal.pone.0120189)
Supplement: S8 Supporting Information — (PDF) [file pone.0120189.s008.pdf]

S8 Supporting Information. Sensitivity analysis, using only the ‘Unselected cohort studies’, of the best-evidence synthesis for *BRCA1* (panel A) and *BRCA2* (panel B) mutation carriership and breast cancer prognosis.

Sensitivity analyses were performed by using only the ‘Unselected cohort studies’.

A. Best-evidence synthesis for *BRCA1* mutation carriership in relation to survival; only ‘Unselected cohort studies’ used

Type of survival	Unadjusted/ adjusted[a]	Studies reporting a worse survival[b] % (n / total n)		Studies reporting a better survival[c] % (n / total n)		Evidence[d] (based on all studies)	Evidence[d] (based on HQ studies)
		Low quality	High quality	Low quality	High quality		
Overall	Unadjusted	**100 (1/1)**	**46 (5/11)**	**0 (0/1)**	**9 (1/11)**	**Moderate**	**Indecisive**
	Adjusted	NA	**60 (3/5)**	NA	**20 (1/5)**	**Moderate**	
BC-specific	Unadjusted	**0 (0/1)**	**50 (3/6)**	**0 (0/1)**	**17 (1/6)**	**Indecisive**	**Indecisive**
	Adjusted	NA	**25 (1/4)**	NA	**75 (3/4)**	**Nil**	
Metastasis-free	Unadjusted	NA	67 (2/3)	NA	33 (1/3)	Indecisive*	
	Adjusted	NA	67 (2/3)	NA	33 (1/3)	Indecisive*	
Recurrence-free	Unadjusted	NA	**50 (2/4)**	NA	**25 (1/4)**	**Indecisive**	
	Adjusted	NA	NA	NA	NA	Indecisive*	

B. Best-evidence synthesis for *BRCA2* mutation carriership in relation to survival; only ‘Unselected cohort studies’ used

Type of survival	Unadjusted/ adjusted[a]	Studies reporting a worse survival[b] % (n / total n)		Studies reporting a better survival[c] % (n / total n)		Evidence[d] (based on all studies)	Evidence[d] (based on HQ studies)
		Low quality	High quality	Low quality	High quality		
Overall	Unadjusted	NA	**50 (3/6)**	NA	**17 (1/6)**	**Indecisive**	
	Adjusted	0 (0/1)	100 (1/1)	100 (1/1)	0 (0/1)	Indecisive*	Indecisive*
BC-specific	Unadjusted	**0 (0/1)**	**50 (2/4)**	**0 (0/1)**	**25 (1/4)**	**Indecisive**	**Indecisive**
	Adjusted	NA	50 (1/2)	NA	0 (0/2)	Indecisive*	
Metastasis-free	Unadjusted	NA	NA	NA	NA	Indecisive*	
	Adjusted	NA	NA	NA	NA	Indecisive*	
Recurrence-free	Unadjusted	NA	0 (0/1)	NA	0 (0/1)	Indecisive*	
	Adjusted	NA	NA	NA	NA	Indecisive*	

[a]Adjusted survival is based on risk estimates adjusted for clinico-pathological characteristics and/or treatment; [b]Worse survival for univariate (unadjusted) outcomes: unadjusted HR >= 1.14 or 5-year absolute survival difference >= 10% or 10-year absolute survival difference >= 10% (when the 5 and 10 year survival differences go in opposite directions, we decided there was no difference in survival). Worse survival for multivariate (adjusted) outcomes: adjusted HR >= 1.14; [c]Better survival for univariate (unadjusted) outcomes: unadjusted HR <= 0.88 or 5-year absolute survival difference >= 10% or 10-year absolute survival difference >= 10% (when the 5 and 10 year survival differences go in opposite directions, we decided there was no difference in survival). Better survival for multivariate (adjusted) outcomes: adjusted HR <= 0.88; [d]See S2 Supporting Information (Best-evidence synthesis). Strong evidence: more than 75% of the HQ studies reported a worse survival; moderate evidence: 60-75% of the HQ studies reported a worse survival and less than 25% of the HQ studies reported a better survival / 50-60% of the HQ studies reported a worse survival and less than 10% of the HQ studies reported a better survival; nil evidence: more than 60% of the HQ studies reported a better survival or no association / more than 40% of the HQ studies reported a better survival; indecisive e evidence: all other options / less than four HQ studies available (*).
