# Supplementary material for: Worse Breast Cancer Prognosis of BRCA1/BRCA2 Mutation Carriers: What's the Evidence? A Systematic Review with Meta-Analysis
Source: PLoS One. 2015 Mar 27;10(3):e0120189. doi: 10.1371/journal.pone.0120189 (PMC4376645; doi:10.1371/journal.pone.0120189)
Supplement: S9 Supporting Information — (PDF) [file pone.0120189.s009.pdf]

S9 Supporting Information. Summary of the sensitivity analysis of the best-evidence synthesis for *BRCA1* (panel A), *BRCA2* (panel B) and *BRCA1/2* (panel C) mutation carriership and breast cancer prognosis.

Sensitivity analyses are performed by changing the cut-offs for an effect and using only the significant results (still only the HQ studies are used for the best evidence synthesis).

A. Sensitivity analysis of the best-evidence synthesis: *BRCA1* mutation carriership

		**Survival**	**Evidence|| based on HQ studies**			
				**Original cut-offs:**		
**Type of survival**	**Unadjusted/ adjusted[a]**	**Better:**	**>=5% / HR <=0.94**	**>=10% / HR <=0.88**	**>=15% / HR <=0.82**	**p < 0.05**
		**Worse:**	**>=5% / HR >=1.07**	**>=10% / HR >=1.14**	**>=15% / HR >=1.22**	**p < 0.05**
Overall	Unadjusted		**Indecisive**	**Indecisive**	**Nil**	**Nil**
	Adjusted		**Moderate**	**Indecisive**	**Indecisive**	**Nil**
BC-specific	Unadjusted		**Moderate**	**Indecisive**	**Indecisive**	**Nil**
	Adjusted		**Indecisive**	**Indecisive**	**Nil**	**Nil**
Metastasis-free	Unadjusted		**Indecisive**	**Indecisive**	**Indecisive**	**Indecisive**
	Adjusted		**Indecisive**	**Indecisive**	**Indecisive**	**Nil**
Recurrence-free	Unadjusted		**Moderate**	**Moderate**	**Moderate**	**Nil**
	Adjusted		Indecisive*	Indecisive*	Indecisive*	Indecisive*

B. Sensitivity analysis of the best-evidence synthesis: *BRCA2* mutation carriership

		**Survival**	**Evidence[b] based on HQ studies**			
				**Original cut-offs:**		
**Type of survival**	**Unadjusted/ adjusted[a]**	**Better:**	**>=5% / HR <=0.94**	**>=10% / HR <=0.88**	**>=15% / HR <=0.82**	**p < 0.05**
		**Worse:**	**>=5% / HR >=1.07**	**>=10% / HR >=1.14**	**>=15% / HR >=1.22**	**p < 0.05**
Overall	Unadjusted		**Moderate**	**Indecisive**	**Nil**	**Nil**
	Adjusted		Indecisive*	Indecisive*	Indecisive*	Indecisive*
BC-specific	Unadjusted		**Indecisive**	**Indecisive**	**Moderate**	**Nil**
	Adjusted		Indecisive*	Indecisive*	Indecisive*	Indecisive*
Metastasis-free	Unadjusted		Indecisive*	Indecisive*	Indecisive*	Indecisive*
	Adjusted		Indecisive*	Indecisive*	Indecisive*	Indecisive*
Recurrence-free	Unadjusted		Indecisive*	Indecisive*	Indecisive*	Indecisive*
	Adjusted		Indecisive*	Indecisive*	Indecisive*	Indecisive*

C. Sensitivity analysis of the best-evidence synthesis: *BRCA1/2* mutation carriership

		**Survival**	**Evidence[b] based on HQ studies**			
				**Original cut-offs:**		
**Type of survival**	**Unadjusted/ adjusted[a]**	**Better:**	**>=5% / HR <=0.94**	**>=10% / HR <=0.88**	**>=15% / HR <=0.82**	**p < 0.05**
		**Worse:**	**>=5% / HR >=1.07**	**>=10% / HR >=1.14**	**>=15% / HR >=1.22**	**p < 0.05**
Overall	Unadjusted		**Nil**	**Nil**	**Nil**	**Nil**
	Adjusted		Indecisive*	Indecisive*	Indecisive*	Indecisive*
BC-specific	Unadjusted		**Indecisive**	**Indecisive**	**Indecisive**	**Moderate**
	Adjusted		Indecisive*	Indecisive*	Indecisive*	Indecisive*
Metastasis-free	Unadjusted		**Indecisive**	**Indecisive**	**Indecisive**	**Indecisive**
	Adjusted		Indecisive*	Indecisive*	Indecisive*	Indecisive*
Recurrence-free	Unadjusted		**Indecisive**	**Indecisive**	**Nil**	**Nil**
	Adjusted		Indecisive*	Indecisive*	Indecisive*	Indecisive*

[a]Adjusted survival is based on risk estimates adjusted for clinico-pathological characteristics and/or treatment; [b]See S2 Supporting Information (Best-evidence synthesis). Strong evidence: more than 75% of the HQ studies reported a worse survival; moderate evidence: 60-75% of the HQ studies reported a worse survival and less than 25% of the HQ studies reported a better survival / 50-60% of the HQ studies reported a worse survival and less than 10% of the HQ studies reported a better survival; nil evidence: more than 60% of the HQ studies reported a better survival or no association / more than 40% of the HQ studies reported a better survival; indecisive e evidence: all other options / less than four HQ studies available (*).
