# Supplementary material for: Worse Breast Cancer Prognosis of BRCA1/BRCA2 Mutation Carriers: What's the Evidence? A Systematic Review with Meta-Analysis
Source: PLoS One. 2015 Mar 27;10(3):e0120189. doi: 10.1371/journal.pone.0120189 (PMC4376645; doi:10.1371/journal.pone.0120189)
Supplement: S10 Supporting Information — (PDF) [file pone.0120189.s010.pdf]

S10 Supporting Information. Confounding and/or mediating factors.

A. Differences in confounders and mediating factors between *BRCA1* and *BRCA2* mutation carriers compared to 'non-carriers' reported in studies included in this review.

Factors* →	Higher grade tumours		Higher stage tumours		LN+ tumours		Large size tumours		ER+ tumours		CT received		HT received	
	N	%	N	%	N	%	N	%	N	%	N	%	N	%
BRCA1														
Studies describing differences of the factor* for *BRCA1* carriers compared to 'non-carriers'[a]	19	*4.2*	12	*26.7*	22	*48.9*	19	*42.2*	19	*42.2*	11	*24.4*	12	*26.7*
Studies reporting a lower percentage of the factor* in *BRCA1* carriers compared to 'non-carriers'	0	*0*	1	*8.3*	6	*27.3*	2	*10.5*	19	*100*	0	*0*	10	*83.3*
Studies reporting a equal distribution of the factor* in *BRCA1* carriers compared to 'non-carriers'	3	*15.8*	10	*83.3*	12	*54.5*	13	*68.4*	0	*0*	5	*45.5*	2	*16.7*
Studies reporting a higher percentage of the factor* in *BRCA1* carriers compared to 'non-carriers'	16	*84.2*	1	*8.3*	4	*18.1*	4	*21.1*	0	*0*	6	*54.5*	0	*0*
BRCA2														
Studies describing differences of the factor* for *BRCA2* carriers compared to 'non-carriers'[b]	7	*29.2*	8	*33.3*	13	*54.2*	9	*37.5*	12	*50*	4	*16.7*	5	*20.8*
Studies reporting a lower percentage of the factor* in *BRCA2* carriers compared to 'non-carriers'	0	*0*	0	*0*	1	*7.7*	0	*0*	1	*8.3*	0	*0*	1	*20*
Studies reporting a equal distribution of the factor* in *BRCA2* carriers compared to 'non-carriers'	4	*57.1*	4	*50*	6	*46.2*	6	*66.7*	8	*66.7*	3	*75*	4	*80*
Studies reporting a higher percentage of the factor* in *BRCA2* carriers compared to 'non-carriers'	3	*42.9*	4	*50*	6	*46.2*	3	*33.3*	3	*25*	1	*25*	0	*0*

[a]Selection of studies included in this review and reporting any risk estimate for survival (independent of the type) of BRCA1 mutation carriers compared to 'non-carriers' (total n = 45 [1-38]); [b]Selection of studies included in this review and reporting any risk estimate for survival (independent of the type) of BRCA2 mutation carriers compared to 'non-carriers' (total n = 24 [5,6,9,12,14,16,19,21,22,26,27,29,30,32,35,36,38-42]). LN+ tumours = tumours with spread to the lymph nodes at diagnosis; ER+ tumours = tumours which have expression of the estrogen receptor; CT received = chemotherapy received as adjuvant treatment; HT received = hormonal therapy received as adjuvant treatment.

The differences in tumour characteristics of *BRCA1* and *BRCA2* mutation carriers compared to 'non-carriers' are described in about half of the studies included in this review (depending on the type of tumour characteristic, table above). When reported, mostly differences in grade and/or stage (defined as stage and/or lymph node status and/or size of the tumour) and/or estrogen receptor status were determined. Most of the studies [5,7,8,11,13,16-18,24-26,32,35,36,38] reporting differences in grade of tumours in *BRCA1* mutation carriers compared to 'non-carriers' [5,7,8,11,13,16-18,23-26,28,32,34-36,38] observed that the percentage of high grade tumours was larger in *BRCA1* mutation carriers compared to 'non-carriers' (84.2% of the studies; table above). For *BRCA2* mutation carriers, of the studies reporting differences in grade of tumours in *BRCA2* mutation carriers compared to 'non-carriers' [5,6,9,12,14,16,19,21,22,26,27,29,30,32,35,36,38-42], most [5,6,9,12,14,16,19,21,22,27,29,30,40-42] observed an equal distribution of high grade tumours in *BRCA2* mutation carriers and 'non-carriers' (57.1% of the studies), although none of the studies reported a lower percentage (table above). In contrast, most studies [4,14,25,27,34-38] reporting differences in stage of tumours observed an equal distribution of high stage tumours in *BRCA1* mutation carriers compared to 'non-carriers'. For *BRCA2* mutation carriers half [14,16,41] of the studies reported a higher percentage of high stage tumours in *BRCA2* mutation carriers compared to 'non-carriers', while none of the studies reported a lower percentage (table above). All studies reporting differences in estrogen receptor status of tumours in *BRCA1* mutation carriers compared to 'non-carriers' [3-5,7,13,17,18,21,22,24-27,32,35-38] observed a higher percentage of estrogen receptor negative tumours in *BRCA1* mutation carriers compared to 'non-carriers'. This was not observed for *BRCA2* mutation carriers (table above) [21,22,26,27,32,35,36,38-40,42].

A small part of the studies included in this review also reported differences in therapy given for breast cancer in *BRCA1* and *BRCA2* mutation carriers compared to 'non-carriers' (table above). About half of the studies [3,21,22,25,43] observed that a higher percentage of *BRCA1* mutation carriers received chemotherapy compared to non-carriers (none of the studies reported that a lower percentage of *BRCA1* mutation carriers received chemotherapy). Furthermore, most of the studies [3,18,21,24,25,32,34,36,38] (83.3%) reported that a lower percentage of hormone therapy was given to *BRCA1* mutation carriers compared to 'non-carriers'. Such differences were not seen when looking at studies reporting differences in treatment given to *BRCA2* mutation carriers compared to 'non-carriers' (table above) [21,22,32,36,38].

B. Effect of adjustment for tumour characteristics and/or treatment on Hazard ratios.

To examine the effect of adjustment for confounders on the prognosis of *BRCA1* and *BRCA2* mutation carriers, we compared pairs of unadjusted HR (HRunadjusted) and adjusted HR (HRadjusted). Because of the low numbers of HR pairs per outcome and the large differences in confounders/mediating variables adjusted for, we could not stratify for these factors in the analysis.

Twenty-three unadjusted plus adjusted HR pairs have been reported for the relation between *BRCA1* carriership and survival [3,4,13,16,18,24,25,27,32,34,38]. In 13 HR pairs [3,4,13,16,18,24,27,32,38] a worse unadjusted survival for *BRCA1* mutation carriers compared to 'non-carriers' was reported (HRunadjusted>1); after adjustment for confounders/mediating variables, in nine pairs [13,16,18,24,27,38] (75%) the HR became weaker (but still in the same direction) or changed to the direction of a better prognosis (HRadjusted<1). For the other four HR pairs [3,4,27,32] (31%), the adjusted estimates were equal (difference HRs<0.1) (n=1 [27]) or became stronger (n=3 [3,4,32]). Furthermore, there were nine HR pairs [25,32,34] with a better unadjusted survival for *BRCA1* mutation carriers compared to 'non-carriers' (HRunadjusted<1). In the majority of these pairs the effect was still in the same direction after adjustment; stronger in three pairs [25] (33%), equal (difference HRs<0.1) in two pairs [32,34] (22%) and weaker in two pairs [34] (22%). In two pairs [32] (22%) the effect changed to a worse prognosis (HRadjusted>1) (Table 4).

Ten unadjusted plus adjusted HR pairs have been reported for the relation between *BRCA2* carriership and survival [27,29,38-42]. In seven HR pairs [27,29,38,39,41] a worse unadjusted survival for *BRCA2* mutation carriers compared to 'non-carriers' was observed (HRunadjusted>1); six of these [27,29,38,39,41] (86%) reported a weaker effect in the same direction or even to the direction of a better prognosis after adjustment. Only in one [27] (14%) of these HR pairs the adjusted estimate was stronger. For the three other HR pairs [40,42] with a better unadjusted survival for *BRCA2* mutation carriers (HRunadjusted<1), one [40] showed a stronger effect after adjustment; in the two others [40,42] the effect was equal (difference HRs<0.1) (Table 4).

References

1. Johannsson OT, Ranstam J, Borg A, Olsson H. Survival of BRCA1 breast and ovarian cancer patients: a population-based study from southern Sweden. J Clin Oncol. 1998;16: 397-404.
2. Plakhins G, Irmejs A, Gardovskis A, Subatniece S, Liepniece-Karele I, et al. Underestimated survival predictions of the prognostic tools Adjuvant! Online and PREDICT in BRCA1-associated breast cancer patients. Fam Cancer. 2013.
3. Huzarski T, Byrski T, Gronwald J, Gorski B, Domagala P, et al. Ten-Year Survival in Patients With BRCA1-Negative and BRCA1-Positive Breast Cancer. J Clin Oncol. 2013.
4. Verhoog LC, Brekelmans CT, Seynaeve C, van den Bosch LM, Dahmen G, et al. Survival and tumour characteristics of breast-cancer patients with germline mutations of BRCA1. Lancet. 1998;351: 316-321.
5. Wagner TM, Moslinger RA, Muhr D, Langbauer G, Hirtenlehner K, et al. BRCA1-related breast cancer in Austrian breast and ovarian cancer families: specific BRCA1 mutations and pathological characteristics. Int J Cancer. 1998;77: 354-360.
6. Gaffney DK, Brohet RM, Lewis CM, Holden JA, Buys SS, et al. Response to radiation therapy and prognosis in breast cancer patients with BRCA1 and BRCA2 mutations. Radiother Oncol. 1998;47: 129-136.
7. Ansquer Y, Gautier C, Fourquet A, Asselain B, Stoppa-Lyonnet D. Survival in early-onset BRCA1 breast-cancer patients. Institut Curie Breast Cancer Group. Lancet. 1998;352: 541.
8. Foulkes WD, Wong N, Brunet JS, Begin LR, Zhang JC, et al. Germ-line BRCA1 mutation is an adverse prognostic factor in Ashkenazi Jewish women with breast cancer. Clin Cancer Res. 1997;3: 2465-2469.
9. Lee JS, Wacholder S, Struewing JP, McAdams M, Pee D, et al. Survival after breast cancer in Ashkenazi Jewish BRCA1 and BRCA2 mutation carriers. J Natl Cancer Inst. 1999;91: 259-263.
10. Robson M, Levin D, Federici M, Satagopan J, Bogolminy F, et al. Breast conservation therapy for invasive breast cancer in Ashkenazi women with BRCA gene founder mutations. J Natl Cancer Inst. 1999;91: 2112-2117.
11. Hamann U, Sinn HP. Survival and tumor characteristics of German hereditary breast cancer patients. Breast Cancer Res Treat. 2000;59: 185-192.
12. Chappuis PO, Kapusta L, Begin LR, Wong N, Brunet JS, et al. Germline BRCA1/2 mutations and p27(Kip1) protein levels independently predict outcome after breast cancer. J Clin Oncol. 2000;18: 4045-4052.
13. Stoppa-Lyonnet D, Ansquer Y, Dreyfus H, Gautier C, Gauthier-Villars M, et al. Familial invasive breast cancers: worse outcome related to BRCA1 mutations. J Clin Oncol. 2000;18: 4053-4059.
14. Eerola H, Vahteristo P, Sarantaus L, Kyyronen P, Pyrhonen S, et al. Survival of breast cancer patients in BRCA1, BRCA2, and non-BRCA1/2 breast cancer families: a relative survival analysis from Finland. Int J Cancer. 2001;93: 368-372.
15. Einbeigi Z, Bergman A, Kindblom LG, Martinsson T, Meis-Kindblom JM, et al. A founder mutation of the BRCA1 gene in Western Sweden associated with a high incidence of breast and ovarian cancer. Eur J Cancer. 2001;37: 1904-1909.
16. Goode EL, Dunning AM, Kuschel B, Healey CS, Day NE, et al. Effect of germ-line genetic variation on breast cancer survival in a population-based study. Cancer Res. 2002;62: 3052-3057.
17. Moller P, Borg A, Evans DG, Haites N, Reis MM, et al. Survival in prospectively ascertained familial breast cancer: analysis of a series stratified by tumour characteristics, BRCA mutations and oophorectomy. Int J Cancer. 2002;101: 555-559.
18. Goffin JR, Chappuis PO, Begin LR, Wong N, Brunet JS, et al. Impact of germline BRCA1 mutations and overexpression of p53 on prognosis and response to treatment following breast carcinoma: 10-year follow up data. Cancer. 2003;97: 527-536.
19. Goffin JR, Straume O, Chappuis PO, Brunet JS, Begin LR, et al. Glomeruloid microvascular proliferation is associated with p53 expression, germline BRCA1 mutations and an adverse outcome following breast cancer. Br J Cancer. 2003;89: 1031-1034.
20. Eccles D, Simmonds P, Goddard J, Coultas M, Hodgson S, et al. Familial breast cancer: an investigation into the outcome of treatment for early stage disease. Fam Cancer. 2001;1: 65-72.
21. Robson ME, Chappuis PO, Satagopan J, Wong N, Boyd J, et al. A combined analysis of outcome following breast cancer: differences in survival based on BRCA1/BRCA2 mutation status and administration of adjuvant treatment. Breast Cancer Res. 2004;6: R8-R17.
22. El Tamer M, Russo D, Troxel A, Bernardino LP, Mazziotta R, et al. Survival and recurrence after breast cancer in BRCA1/2 mutation carriers. Ann Surg Oncol. 2004;11: 157-164.
23. Seynaeve C, Verhoog LC, van de Bosch LM, van Geel AN, Menke-Pluymers M, et al. Ipsilateral breast tumour recurrence in hereditary breast cancer following breast-conserving therapy. Eur J Cancer. 2004;40: 1150-1158.
24. Chappuis PO, Donato E, Goffin JR, Wong N, Begin LR, et al. Cyclin E expression in breast cancer: predicting germline BRCA1 mutations, prognosis and response to treatment. Ann Oncol. 2005;16: 735-742.

25. Bonadona V, Dussart-Moser S, Voirin N, Sinilnikova OM, Mignotte H, et al. Prognosis of early-onset breast cancer based on BRCA1/2 mutation status in a French population-based cohort and review. Breast Cancer Res Treat. 2007;101: 233-245.
26. Moller P, Evans DG, Reis MM, Gregory H, Anderson E, et al. Surveillance for familial breast cancer: Differences in outcome according to BRCA mutation status. Int J Cancer. 2007;121: 1017-1020.
27. Rennert G, Bisland-Naggan S, Barnett-Griness O, Bar-Joseph N, Zhang S, et al. Clinical outcomes of breast cancer in carriers of BRCA1 and BRCA2 mutations. N Engl J Med. 2007;357: 115-123.
28. Vinodkumar B, Syamala V, Abraham EK, Balakrishnan R, Ankathil R. Germline BRCA1 mutation and survival analysis in familial breast cancer patients in Kerala; South India. J Exp Clin Cancer Res. 2007;26: 329-336.
29. Heikkinen T, Karkkainen H, Aaltonen K, Milne RL, Heikkila P, et al. The breast cancer susceptibility mutation PALB2 1592delT is associated with an aggressive tumor phenotype. Clin Cancer Res. 2009;15: 3214-3222.
30. Chiappetta G, Ottaiano A, Vuttariello E, Monaco M, Galdiero F, et al. HMGA1 protein expression in familial breast carcinoma patients. Eur J Cancer. 2010;46: 332-339.
31. Cortesi L, Masini C, Cirilli C, Medici V, Marchi I, et al. Favourable ten-year overall survival in a Caucasian population with high probability of hereditary breast cancer. BMC Cancer. 2010;10: 90.
32. Brekelmans CT, Tilanus-Linthorst MM, Seynaeve C, vd Ouweland A, Menke-Pluymers MB, et al. Tumour characteristics, survival and prognostic factors of hereditary breast cancer from BRCA2-, BRCA1- and non-BRCA1/2 families as compared to sporadic breast cancer cases. Eur J Cancer. 2007;43: 867-876.
33. Hagen AI, Tretli S, Maehle L, Apold J, Veda N, et al. Survival in Norwegian BRCA1 mutation carriers with breast cancer. Hered Cancer Clin Pract. 2009;7: 7.
34. Lee LJ, Alexander B, Schnitt SJ, Comander A, Gallagher B, et al. Clinical outcome of triple negative breast cancer in BRCA1 mutation carriers and noncarriers. Cancer. 2011;117: 3093-3100.
35. Xu J, Wang B, Zhang Y, Li R, Wang Y, et al. Clinical implications for BRCA gene mutation in breast cancer. Mol Biol Rep. 2012;39: 3097-3102.
36. Arun B, Bayraktar S, Liu DD, Gutierrez Barrera AM, Atchley D, et al. Response to neoadjuvant systemic therapy for breast cancer in BRCA mutation carriers and noncarriers: a single-institution experience. J Clin Oncol. 2011;29: 3739-3746.
37. Plakhins G, Irmejs A, Gardovskis A, Subatniece S, Rozite S, et al. Genotype-phenotype correlations among BRCA1 4153delA and 5382insC mutation carriers from Latvia. BMC Med Genet. 2011;12: 147.
38. Goodwin PJ, Phillips KA, West DW, Ennis M, Hopper JL, et al. Breast cancer prognosis in BRCA1 and BRCA2 mutation carriers: an International Prospective Breast Cancer Family Registry population-based cohort study. J Clin Oncol. 2012;30: 19-26.
39. Tryggvadottir L, Olafsdottir EJ, Olafsdottir GH, Sigurdsson H, Johannsson OT, et al. Tumour diploidy and survival in breast cancer patients with BRCA2 mutations. Breast Cancer Res Treat. 2013;140: 375-384.
40. Verhoog LC, Brekelmans CT, Seynaeve C, Dahmen G, van Geel AN, et al. Survival in hereditary breast cancer associated with germline mutations of BRCA2. J Clin Oncol. 1999;17: 3396-3402.
41. Loman N, Johannsson O, Bendahl P, Dahl N, Einbeigi Z, et al. Prognosis and clinical presentation of BRCA2-associated breast cancer. Eur J Cancer. 2000;36: 1365-1373.
42. Budroni M, Cesaraccio R, Coviello V, Sechi O, Pirino D, et al. Role of BRCA2 mutation status on overall survival among breast cancer patients from Sardinia. BMC Cancer. 2009;9: 62.
43. Soumittra N, Meenakumari B, Parija T, Sridevi V, Nancy KN, et al. Molecular genetics analysis of hereditary breast and ovarian cancer patients in India. Hered Cancer Clin Pract. 2009;7: 13.
