# Supplementary material for: Worse Breast Cancer Prognosis of BRCA1/BRCA2 Mutation Carriers: What's the Evidence? A Systematic Review with Meta-Analysis
Source: PLoS One. 2015 Mar 27;10(3):e0120189. doi: 10.1371/journal.pone.0120189 (PMC4376645; doi:10.1371/journal.pone.0120189)
Supplement: S11 Supporting Information — (PDF) [file pone.0120189.s011.pdf]

S11 Supporting Information. Figures showing the association between of the percentage of selection bias (panels A and B), misclassification bias (panel C) confounding/accounting for mediating variables (panel D) present in the study and the heterogeneity of results.

Panels a, b and c: results defined as the 5-year overall survival difference for *BRCA1* mutation carriers compared to 'non-carriers'; panel d: results defined as the adjusted hazard ratio for overall survival for *BRCA1* mutation carriers compared to 'non-carriers'. The results are stratified for the type of study (panel a), the percentage of incident cases (panel b), for the testing of the 'non-carrier' comparison group for *BRCA1* mutations (panel c) and for the factors used for adjustment of the hazard ratio (panel d).
CGC based studies with ext. ref. = CGC based studies with external reference group; CGC based studies with int. ref. = CGC based studies with internal reference group.

A.

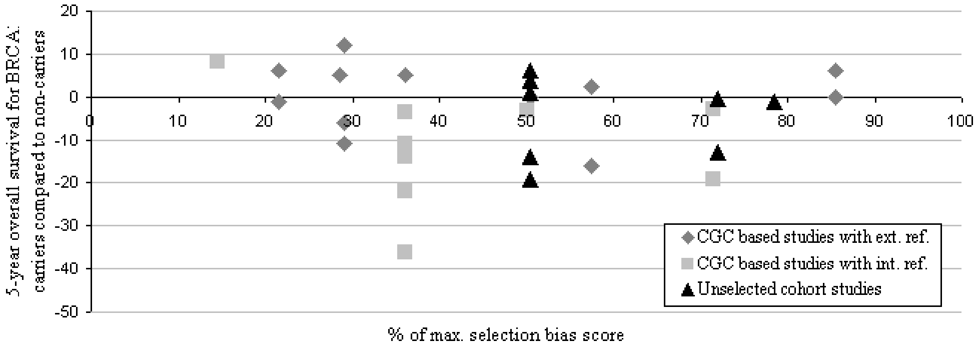

B.

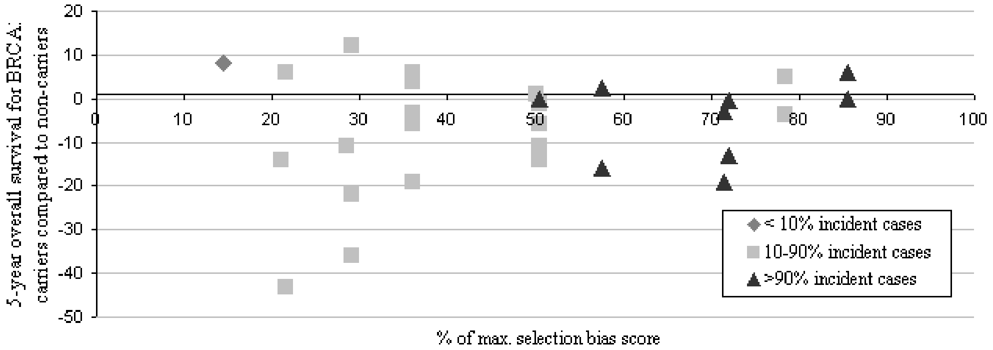

C.

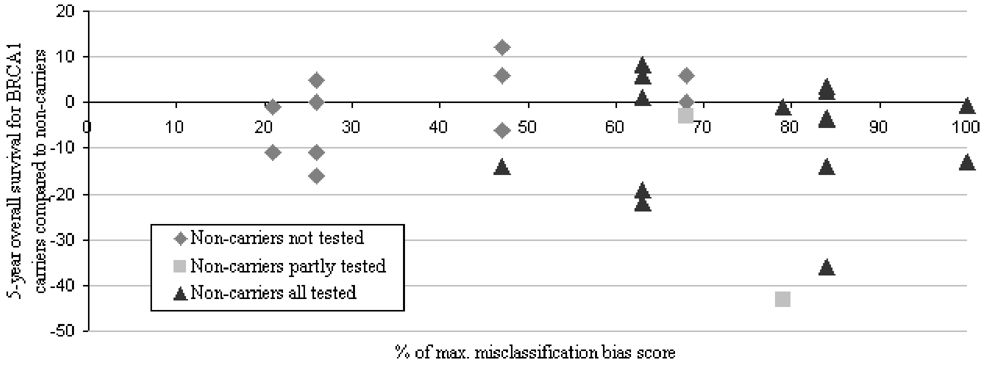

D.

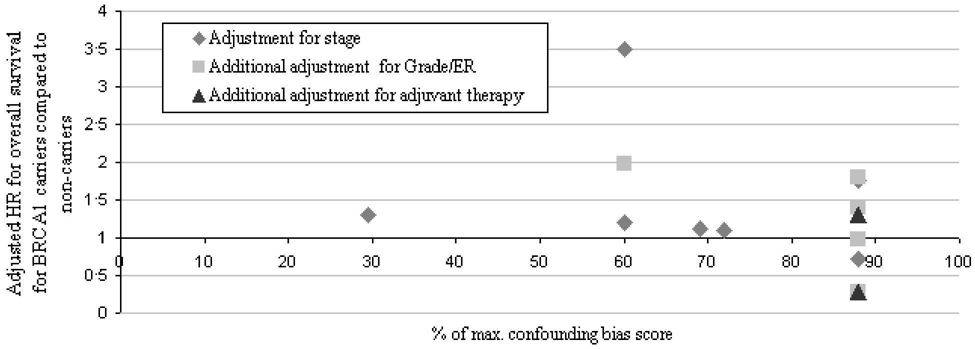

Adjusted HR for overall survival for BRCA1 carriers compared to non-carriers
Adjustment for stage
Additional adjustment for Grade/ER
Additional adjustment for adjuvant therapy
% of max. confounding bias score
