# Supplementary material for: Worse Breast Cancer Prognosis of BRCA1/BRCA2 Mutation Carriers: What's the Evidence? A Systematic Review with Meta-Analysis
Source: PLoS One. 2015 Mar 27;10(3):e0120189. doi: 10.1371/journal.pone.0120189 (PMC4376645; doi:10.1371/journal.pone.0120189)

S12 Supporting Information. Funnel plot showing the number of *BRCA1* mutation carriers included in the study related to the results defined as the 5-year overall survival difference for *BRCA1* mutation carriers compared to 'non-carriers'.

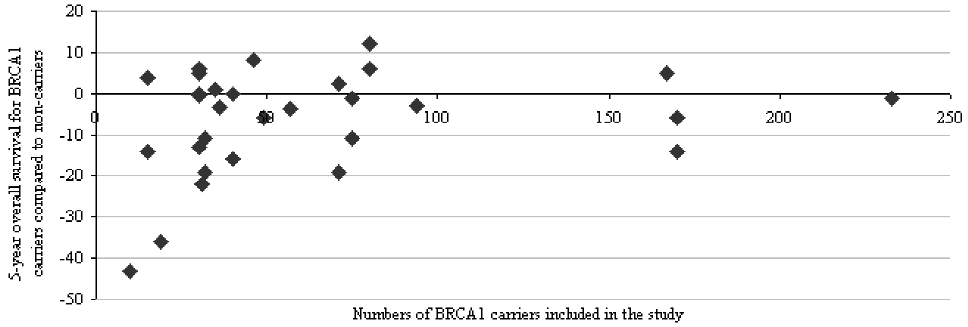

Supplement: S12 Supporting Information — (PDF) [file pone.0120189.s012.pdf]
